# Verification of preparations of (1*H*-indol-3-yl)methyl electrophiles and development of their microflow rapid generation and substitution

**DOI:** 10.1038/s42004-023-00837-1

**Published:** 2023-03-04

**Authors:** Hisashi Masui, Sena Kanda, Shinichiro Fuse

**Affiliations:** Department of Basic Medicinal Sciences, Graduate School of Pharmaceutical Sciences, Nagoya University, Furo-cho, Chikusa-ku, Nagoya, 464-8601 Japan

**Keywords:** Flow chemistry, Synthetic chemistry methodology, Organocatalysis

## Abstract

Although highly reactive (1*H*-indol-3-yl)methyl electrophiles such as (1*H*-indol-3-yl)methyl halides are potential precursors for the synthesis of various indole derivatives, some researchers have reported difficulties in their preparation due to concomitant undesired dimerization/oligomerization. Nevertheless, there have been some reports on the preparation of (1*H*-indol-3-yl)methyl halides. To resolve this contradiction, all the previously reported preparations of (1*H*-indol-3-yl)methyl halides were examined. However, we could not reproduce any of these preparations, and we revised several structures of indole derivatives. Here we show the rapid (0.02 s) and mild (25 °C) generation of an (1*H*-indol-3-yl)methyl electrophile that enables the rapid (0.1 s) and mild (25 °C) nucleophilic substitution in a microflow reactor. Eighteen unprotected indole analogues can be successfully synthesized using the developed microflow nucleophilic substitution with various nucleophiles.

## Abstract (web summary)

(1*H*-indol-3-yl)methyl electrophiles are potential precursors for the synthesis of indole derivatives, however, their preparation remains challenging and only a few syntheses have been reported so far. Here, the authors examine previously reported preparations, clarify inconsistencies within the literature, and develop a highly versatile nucleophilic substitution at the indole 3’-position using microflow technologies.

## Introduction

**Fig. 1:**
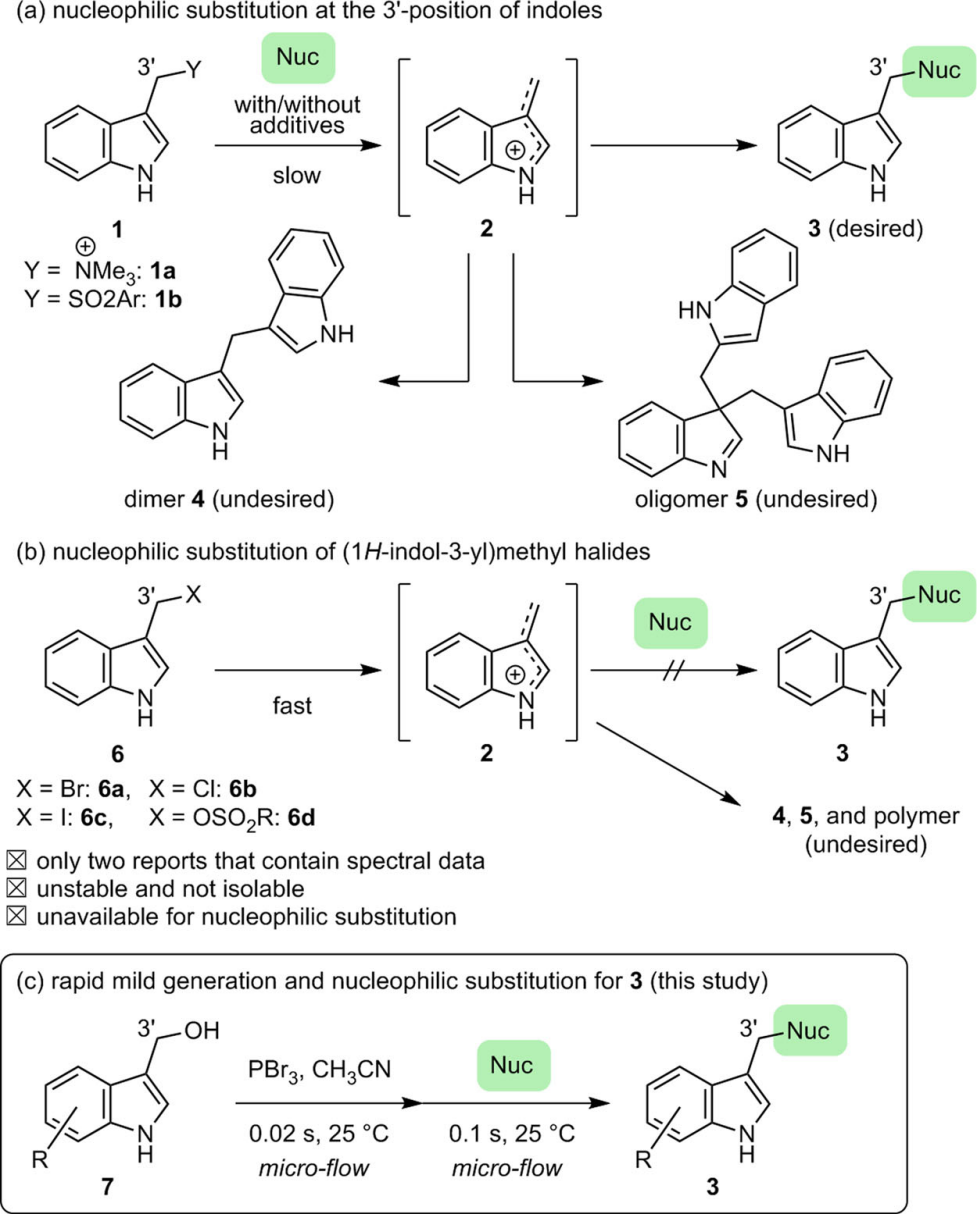
Nucleophilic substitutions at the 3’-position of substituted indoles. **a** Nucleophilic substitution using methylated gramine **1a** and aryl sulfone **1b**. **b** Nucleophilic substitution using (1*H*-indol-3-yl)methyl halide **6**. **c** This study: rapid mild generation and nucleophilic substitution in a microflow reactor.

Indole has been recognized as a privileged structure, ranking 13th among the most frequently used 351 ring systems found in marketed drugs^1,2^. The substituted indoles are useful not only as medicines but also as agrochemicals and functional materials^3–5^. A number of substituted indoles have been synthesized via nucleophilic substitutions at the α-position of the indole. The moderately reactive (1*H*-indol-3-yl)methyl electrophiles **1**, such as methylated gramine **1a** (Y = ^+^NMe_3_)^6,7^ and aryl sulfone **1b** (Y = SO_2_Ar)^8,9^ with or without additives (Fig. 1a) have been used for the nucleophilic substitutions at the 3’-position. However, this approach suffers from the requirement of high temperatures and long reaction times, as well as limited substrate scope^6–9^. In addition, highly electrophilic vinyl iminium intermediate **2** is gradually generated; therefore, the resultant coexistence of substrate **1** and electrophile **2** leads to undesired dimerization and/or oligomerization^6–13^. Although the rapid generation of **2** from highly reactive (1*H*-indol-3-yl)methyl electrophiles **6** containing good leaving groups (Y = halogen, OSO_2_R) can potentially avoid the undesired dimerization/oligomerization, they have been rarely used in the substitution reactions at the 3’-position of the electron-rich indoles (Fig. 1b). The preparation of the highly reactive **6** is difficult due to concomitant undesired dimerization and/or oligomerization^14^. In fact, Eryshev et al. reported that (1*H*-indol-3-yl)methyl bromide (**6a**) could not be prepared via the bromination of indole-3-methanol using PBr_3_, most likely due to the instability of alkyl bromide **6a**^15^. Cook et al. also reported an unsuccessful attempt toward the preparation of **6a** via the bromination of 3-methyl-1*H*-indole using azobis(isobutyronitrile) and *N*-bromosuccinimide^16^. Rhee et al. also reported difficulty in isolating unprotected indole **6** with a good leaving group at the 3’-position^17^. Moreover, even in situ generation of **6** and its use in the following nucleophilic substitution without purification did not afford desired **3**^17^. They concluded that the preparation of (1*H*-indol-3-yl)methyl electrophile **6** is difficult unless **6** contains the electron-withdrawing group^17^.

Despite these reports, SciFinder search revealed five papers that reported the synthesis of (1*H*-indol-3-yl)methyl electrophile **6** (Table 1)^15,18–21^. Two among these also report spectral data for **6**^19,21^. However, based on the previous reports for similar compounds, some discrepancies were identified between the reported and expected data. Three of the five studies involved elemental analysis^15^, TLC analysis^18^, or no analytical data^20^. Besides the aforementioned five studies, five other studies reported nucleophilic substitution of **6**, although the preparation procedure for **6** was not described^22–26^. As far as we could ascertain, the preparation of highly electrophilic (1*H*-indol-3-yl)methyl iodide (**6c**) and sulfonate **6d** have not been reported. Based on SciFinder search, the alkyl bromide **6a** is commercially available in a limited number of countries. However, despite our efforts to import **6a** through trading companies, we were unable to find out a supplier. The alkyl halides **6b** and **6c**, and alkyl sulfonate **6d** are not commercially available. Hence, it is an important pursuit to resolve the abovementioned contradiction in the synthesis of **6a** and **6b**.

**Table 1:** Previous reports for the synthesis and the nucleophilic substitution of (1*H*-indol-3-yl)methyl halide **6**.

Contents	Halogen	Analytical data of 6	References
Synthesis of **6**	Br	Elemental analysis	^15^
		TLC	^18^
		^1^H NMR and IR	^19^
	Cl	None	^20^
		^1^H NMR, ^13^C NMR, and HRMS	^21^
Nucleophilic substitution of **6** (no procedure for preparation of **6**)	Br	None	^22–24^
	Cl	None	^25,26^

Microflow technologies have garnered much attention owing to their advantages over conventional batch synthesis approaches^27–34^. In particular, the microflow reactor allows precise control of the reaction time and temperature, thus enabling the use of highly reactive and unstable species^35–39^. We have developed various efficient synthetic approaches using microflow technologies based on the rapid generation and reaction of unstable and highly active species before side reactions occur^40–42^. We anticipated that we could achieve nucleophilic substitution at the 3’-position of indoles while suppressing dimerization/oligomerization by employing microflow technology.

Herein, we report the examination of all the previously reported preparations of (1*H*-indol-3-yl)methyl halides **6a** and **6b**, and the structural revision of two reported indole derivatives. We also developed a highly versatile nucleophilic substitution at the 3’-position of indoles using microflow technologies (Fig. 1c).

## Results and Discussion

### Synthesis of (1*H*-indol-3-yl)methyl halides

**Fig. 2:**
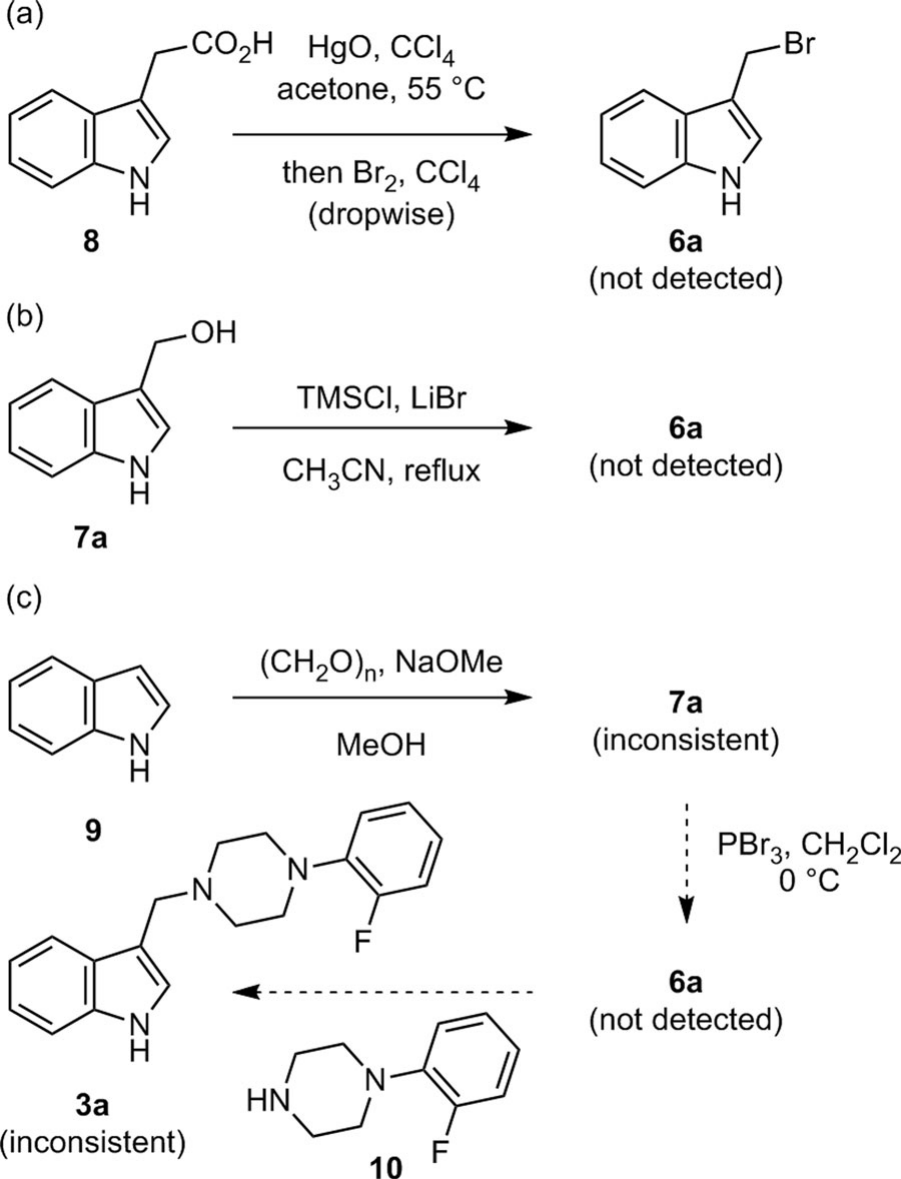
Examination of previously reported syntheses of (1*H*-indol-3-yl)methyl bromide (**6a**) and its derivatizations. **a** Preparation of **6a** via the Borodin–Hunsdiecker reaction reported by Eryshev et al. **b** Preparation of **6a** via bromination with TMSCl and LiBr reported by Scanlan et al. **c** Preparation of **6a** via bromination with PBr_3_ reported by Mekonnen Sanka et al.

We examined all three previously reported syntheses of **6a** and its analogues. The first report by Eryshev et al. includes the preparation of **6a** via the Borodin–Hunsdiecker reaction^15^ (Fig. 2a). The reported yield of the alkyl bromide **6a** was low (28%), and the structural confirmation was performed only by elemental analysis^15^. We examined the following reported procedure several times (for details, see Supplementary Information pages S5–S6). Red mercury (II) oxide was added to a solution of indole-3-acetic acid (**8**) in carbon tetrachloride and acetone. After being stirred at 55 °C for 10 min, bromine was added dropwise. As a result, a highly lachrymatory compound was generated, but the desired alkyl bromide **6a** was not detected by ^1^H NMR spectroscopy and a large amount of red precipitate was generated. A detailed structural analysis of the product could not be performed because it was an inseparable mixture of many products. We speculated that the desired **6a** formed in situ (it probably had lachrymatory nature), but the undesired polymerization of indole analogues underwent concomitantly that led to its precipitation. The second report by Scanlan et al. includes the synthesis of **6a** by bromination with TMSCl and LiBr^18^. They reported only TLC analysis for structural confirmation of **6a** (Fig. 2b). We examined the reported procedure several times (for details, see Supplementary Information pages S6–S7). However, the desired **6a** could not be detected using ^1^H NMR spectroscopy and a large amount of red precipitate was generated most likely due to the undesired oligomerization/polymerization. The third report by Mekonnen Sanka et al. ^19^ includes the synthesis of **6a** by the bromination of **7a** using PBr_3_ (Fig. 2c), which has been reported to be impossible by Eryshev et al.^15^. The ^1^H NMR spectrum for **7a** in their report was obviously different from those in another report^43^. Therefore, we purchased **7a** and measured its ^1^H NMR spectrum (for details, see Supplementary Information page S7 and Supplementary Figure 11 in Supplementary Information page S33). Our observed spectrum was not consistent with that reported by Mekonnen Sanka et al.^19^, whereas, our observed spectrum was well consistent with those in another previous report^43^. Mekonnen Sanka et al. also reported the nucleophilic substitution of alkyl bromide **6a** with an aryl piperazine **10** to obtain **3a**^19^. Although we synthesized **3a** using our developed method described later, the spectral data of **3a** were not consistent with those reported by Mekonnen Sanka et al.^19^, but were consistent with those reported by Akkoc et al.^44^. These results suggest that the structures **6a** and **7a** reported by Mekonnen Sanka et al. are most likely incorrect.

**Fig. 3:**
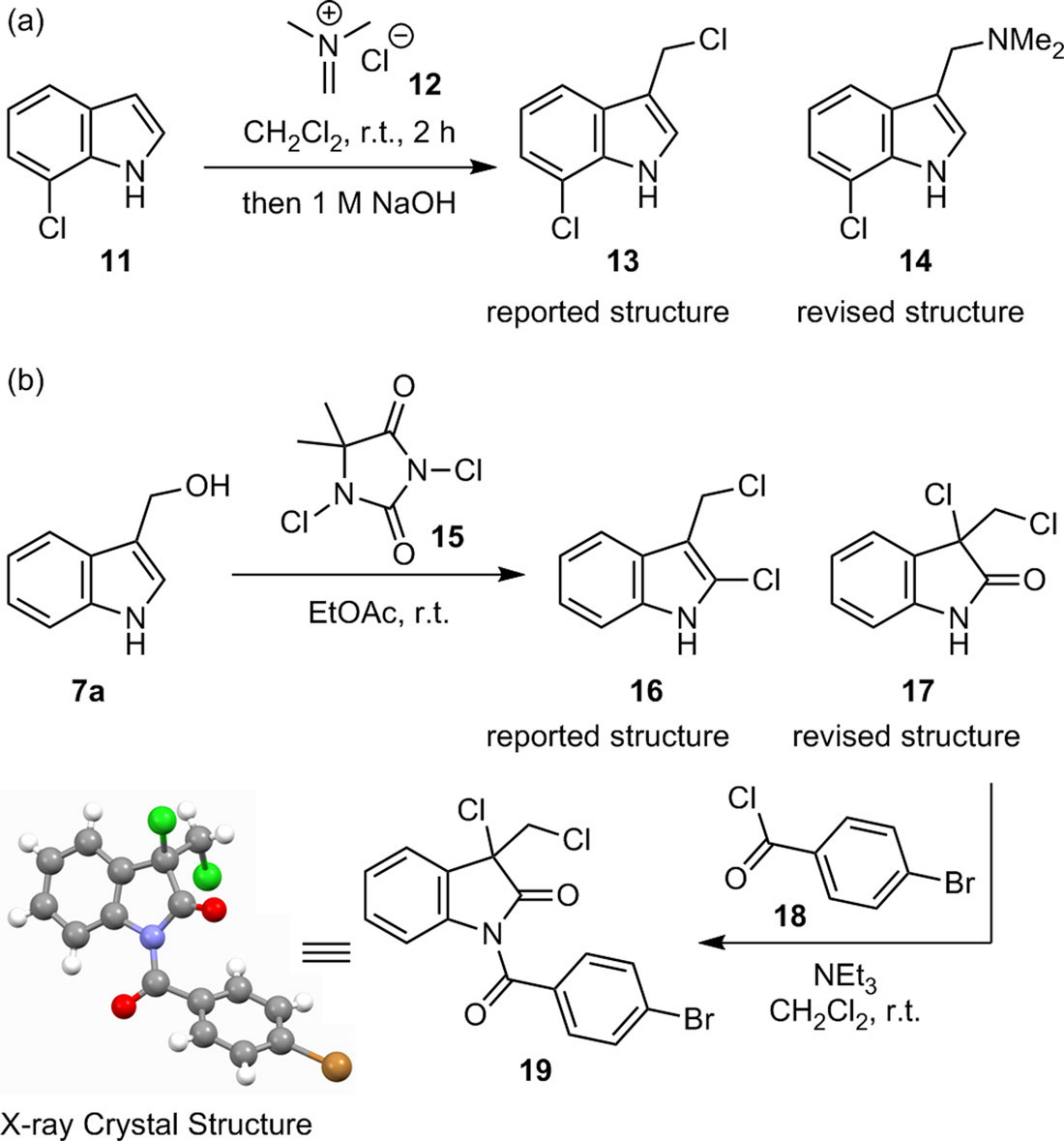
Examination of the reported syntheses of (1*H*-indol-3-yl)methyl chloride analogues. **a** Preparation of **13** using iminium chloride **12** reported by Degterev et al. **b** Preparation of **16** using **15** reported by Jiang et al.

Two papers have reported the syntheses of (1*H*-indol-3-yl)methyl chloride analogues. Degterev et al.^20^ reported that the reaction of 7-chloroindole (**11**) with *N*,*N*-dimethylmethyleneiminium chloride (**12**) afforded alkyl chloride **13**, although no spectral data for **13** was reported. In contrast, the reaction of an indole analogue with *N*,*N*-dimethylmethyleneiminium salt generally provides a gramine analogue^45^. In fact, Faul et al. reported that the same reaction of **11** and **12** afforded **14** and not **13**^46^. We examined the reaction according to the procedure reported by Degterev et al. and obtained a gramine analogue **14**. However, alkyl chloride **13** could not be detected (Fig. 3a). The spectral data of our obtained **14** were consistent with those reported by Faul et al.^46^. The synthesis of **16** via chlorination of **7a** using **15** (Fig. 3b) was reported by Jiang et al.^21^. We carried out the reaction according to the procedure reported by Jiang et al. The reaction proceeded well, and the spectral data of the obtained compound was consistent with those reported by Jiang et al.^21^. However, the structural determination by Jiang et al. had some concerns, especially in the ^13^C NMR data. (1) A signal at 174.1 ppm was observed, corresponding to a carbonyl carbon, although the proposed structure of **16** does not have a carbonyl group. (2) Six signals in the aromatic region (140.8–111.1 ppm), and two signals in the aliphatic region (63.2 and 45.9 ppm) were observed, although the proposed structure **16** has eight aromatic and one aliphatic carbons. Thus, we converted the obtained product to the corresponding benzamide **19** using *p*-bromobenzoyl chloride (**18**), and its structure was unambiguously determined by X-ray crystallography^47^. The analysis indicated that benzamide **19** has an oxyindole structure. Thus, we conclude that the chemical structure of the product from the reaction between **7a** and **15** is not **16**, but **17**, which is consistent with the ^13^C NMR data.

Although we examined all the previously reported syntheses of (1*H*-indol-3-yl)methyl halides and their reactions, we could not reproduce the reported results and could not confirm the generation of (1*H*-indol-3-yl)methyl halides. Additionally, our examinations unexpectedly led to revisions of previously reported structures of indole derivatives. These results clearly indicate the importance of the development of reliable and practical nucleophilic substitution approaches using highly active (1*H*-indol-3-yl)methyl electrophile. To achieve this goal, we examined the in situ generation of the (1*H*-indol-3-yl)methyl electrophile via the halogenation/sulfonylation of stable and readily available indole-3-methanol (**7a**) and its subsequent nucleophilic substitution with NaN_3_. The reaction time and temperature were precisely controlled using microflow technologies^35–39^.

### Development of microflow nucleophilic substitution

First, we examined the activation reagents (Table 2, entries 1–11). When trivalent phosphorus reagents PBr_3_ and PCl_3_ were employed, the azidation proceeded smoothly in yields greater than 50% (entries 1 and 2). By contrast, none of the other reagents afforded satisfactory results (entries 3–11). Although quantitative data for the electrophilicity of the activating reagents are not available, we speculated that the electrophilicity of the reagents is important for obtaining good yields. On one hand, the use of highly reactive trivalent phosphorus electrophiles afforded relatively high yields (entries 1 and 2). On the other hand, the use of less reactive pentavalent phosphorus and carbon electrophiles, including POCl_3_, AcBr, AcCl, and Ac_2_O, resulted in the recovery of a large amount of alcohol **7a** (entries 3–6) with concomitant generation of insoluble solids (entries 4 and 6). The use of sulfur electrophiles with medium levels of reactivity led to the recovery of alcohol **7a** and/or the generation of insoluble solids probably due to undesired dimerization/oligomerization (entries 7–11). Then, we examined the activation time (Table 2, entries 1 and 12–14). Extension of activation time dramatically reduced the yield, along with the generation of insoluble solids (entry 1 vs. entries 12–14). When activation was carried out for 0.5 s, azide **3b** was obtained in only 20% yield and **7a** was not recovered (entry 14). We could not examine reaction times shorter than 0.02 s because neither the length nor the inner diameter of the reaction tube could be reduced further (for details, see Supplementary Information page S13). The use of a reduced quantity of PBr_3_ (0.35 equiv.) and activation time (0.02 s) improved the yield of **3b** (84%, entry 15). When the reaction was performed at 0 °C, the yield was somewhat low (74%) (entry 16). When the highly electrophilic intermediate is gradually generated at lower temperatures, the substrate coexists with the electrophile for a longer time and causes undesired oligomerization. The key to suppressing the side reaction is that the electrophile is rapidly generated at a higher temperature and immediately used for the next reaction (details, see Supplementary Information page S15). To suppress undesired intermolecular reactions, such as dimerization/oligomerization of (1*H*-indol-3-yl)methyl electrophile, diluted conditions (0.0500 M for a solution of **7a**) were examined (entry 17). The desired product **3b** was obtained in excellent yields with sufficient reproducibility (93 ± 2%). It should be noted that the desired product was not detected and the precipitates that appear to be dimer/oligomers were generated in three independent experiments under batch conditions (entry 18), although the reaction mixture was vigorously mixed (1000 rpm) during the experiment (caution, care should be taken when performing the reaction under batch conditions because the reaction is exothermic and rapidly generates dangerous gas such as HBr). These results clearly indicated the instability of the highly active (1*H*-indol-3-yl)methyl electrophile. The rapid (0.02 s) and mild (25 °C) in situ generation of the extremely reactive (1*H*-indol-3-yl)methyl electrophile enabled rapid (0.1 s) and mild (25 °C) nucleophilic substitution.

**Table 2:** Optimization for in situ generation of (1*H*-indol-3-yl)methyl electrophile from **7a** and its nucleophilic substitution with NaN_3_ in a microflow reactor.


Entry	Reagent	X (Equiv.)^a^	Time (s)	Yield (%)^b^	
				3b	7a
1	PBr_3_	0.500	0.02	77	Trace
2	PCl_3_	0.500	0.02	52	4
3	POCl_3_	0.500	0.02	n.d.	>99
4^c^	AcBr	1.50	0.02	4	61
5^c^	AcCl	1.50	0.02	n.d.	>99
6^c^	Ac_2_O	1.50	0.02	n.d.	67
7^c^	Tf_2_O	1.50	0.02	8	38
8^c^	MsCl	1.50	0.02	29	41
9^c^	TsCl	1.50	0.02	n.d.	n.d.
10	SOBr_2_	0.750	0.02	7	23
11	SOCl_2_	0.750	0.02	15	16
12	PBr_3_	0.500	0.05	60	n.d.
13	PBr_3_	0.500	0.1	54	n.d.
14	PBr_3_	0.500	0.5	20	n.d.
15	PBr_3_	0.350	0.02	84	n.d.
16^d^	PBr_3_	0.350	0.02	74	n.d.
17^e^	PBr_3_	0.350	0.02	93 ± 2^f^	n.d.
18^e^^,^^g^	PBr_3_	0.350	10	n.d.^f^	n.d.

^a^The reagent quantities were changed based on the reaction mechanism. Theoretically, 1 equiv. of PBr_3_, PCl_3_, or POCl_3_ can convert 3 equiv. of alcohol to the alkyl halide, 1 equiv. of SOBr_2_, or SOCl_2_ can convert 2 equiv. of alcohol, and 1 equiv. of AcBr, AcCl, Ac_2_O, Tf_2_O, MsCl, or TsCl can convert equimolar quantities of alcohol.

^b^Yields were determined by ^1^H NMR analysis using 1,1,2-trichloroethane as an internal standard.

^c^NEt_3_ was added with a solution of **7a** instead of a solution of NaN_3_.

^d^The reaction was carried out at 0 °C.

^e^0.0500 M solution of **7a** was used.

^f^Three independent experiments were performed.

^g^Reaction mixture was magnetically stirred (1000 rpm) under batch conditions.

**Fig. 4:**
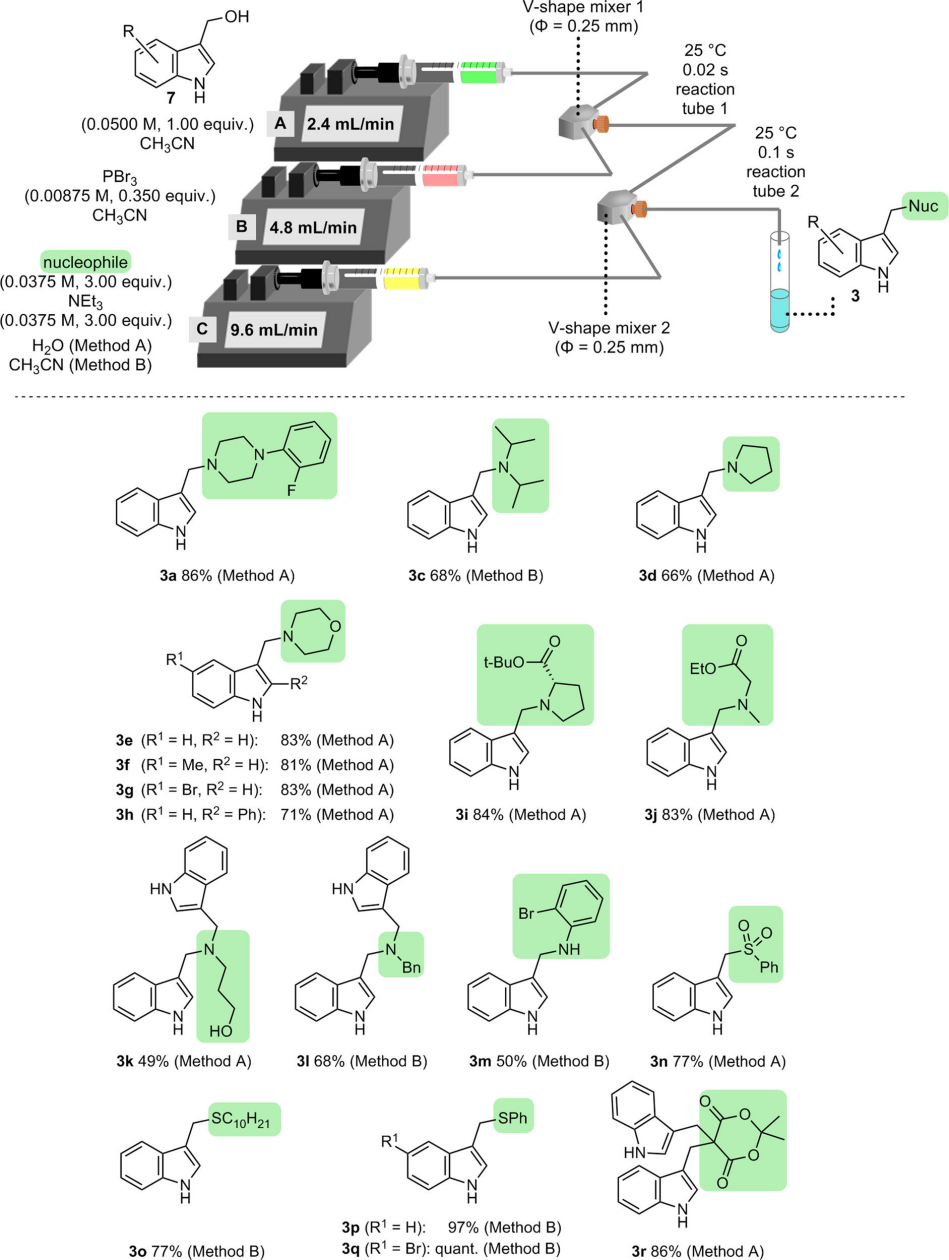
Scope of the developed microflow nucleophilic substitution. Unless stated otherwise, a solution of substrate **7** (0.0500 M, 1.00 equiv.) in CH_3_CN, a solution of PBr_3_ (0.00875 M, 0.350 equiv.) in CH_3_CN, and a solution of nucleophile (0.0375 M, 3.00 equiv.) and NEt_3_ (0.0375 M, 3.00 equiv.) were introduced at 25 °C with syringe pumps A, B, and C, respectively. The nucleophile is described in green. Hydrophilic nucleophiles were used in the form of aqueous solutions (Method A), whereas hydrophobic nucleophiles were used in CH_3_CN solutions (Method B).

The substrate scope of the developed approach was examined (Fig. 4), after optimizing the base used, amount of PBr_3_, and temperature (for details, see Supplementary Information pages S14–S17). Hydrophilic nucleophiles were used in the form of aqueous solutions (Method A), whereas hydrophobic nucleophiles were used in CH_3_CN solutions (Method B). The use of secondary amines as nucleophiles afforded the desired tertiary amines **3a** and **3c**–**3e** in good yields (66–86% yields). Compound **3a** was successfully synthesized in high yield (86%). The products **3f** and **3g** containing an electron-donating and an electron-withdrawing group, respectively, at the 5-position of the indole ring were obtained in high yields (81% and 83%, respectively). The product **3h** with a bulky phenyl group at the 2-position of the indole ring was obtained without a significant decrease in the yield (71%). The use of amino-acid-derived secondary amines as *N*-nucleophiles afforded products **3i** and **3j** high yields (84% and 83%, respectively). When primary alkylamines were used as nucleophiles, tertiary amines **3k** and **3l** were obtained in 49% and 68% yields, respectively, via double indolylmethylation. When 2-bromoaniline with low nucleophilicity was employed, single indolylmethylation occurred to afford **3m** in 50% yield. The use of *S*-nucleophiles, including sodium benzenesulfonate, alkyl thiol, and aryl thiol, afforded the desired products **3n**–**3q** in good-to-excellent yields (77%–quant.). The use of Meldrum’s acid as *C*-nucleophile afforded the double indolylmethylated product **3o** in good yield (86%). The indole analogues **3a**–**3r** were soluble in the commonly used organic solvent such as ethyl acetate, dichloromethane, acetonitrile, and chloroform. As described above, nucleophilic substitutions of **1** (Fig. 1a) require high temperatures and long reaction times and involve undesired dimerization/oligomerization. By contrast, the developed approach enables rapid nucleophilic substitutions with a variety of *N*-, *S*-, and *C*-nucleophiles.

**Fig. 5:**
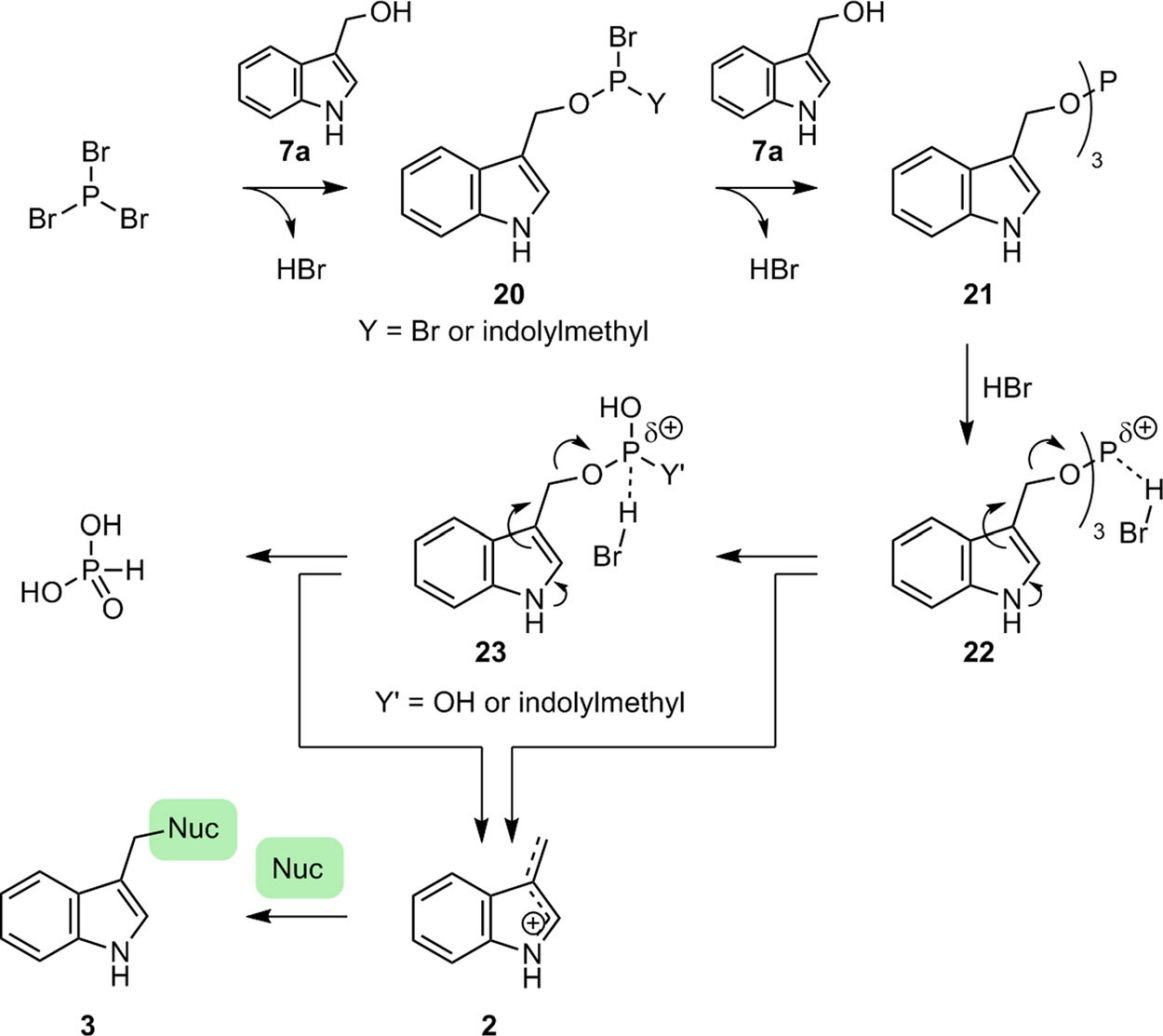
Plausible mechanism. Plausible mechanism for rapid and mild generation and nucleophilic substitution of extremely active (1*H*-indol-3-yl)methyl electrophile **2**.

A plausible reaction mechanism is shown in Fig. 5. It was reported that coupling between PBr_3_ and alcohol **7a** is fast and the corresponding phosphite **21** is generated through intermediate **20**^48–50^. Gerrard reported that very fast proton trapping by **21** is key for enhancing the electrophilicity of the phosphorus center in **21**^48^. It is conceivable that the elimination of the extremely electrophilic (1*H*-indol-3-yl)methyl cation species **2** from **22** occurs rapidly. Previous studies have indicated that the second and third reactions are slower than the first reaction from **22**^48^. However, these second and third elimination reactions of **2** via **23** may be facilitated by the electron-donating ability of the indole ring. Reportedly, proton trapping by the intermediate **20**, which leads to the electrophilic activation of the phosphorus center in **20**, is rather slow^48^. However, we could not exclude the possibility of generating **2** via the activation of **20**. In fact, Hudson reported that dealkylations of ROPBr_2_ and (RO)_2_PBr like **20** that afford **2** are also possible^50^. We speculated that the entire process shown below is rapid and the rapid generation of **2** in a microflow reactor avoided any undesired dimerization/oligomerization. This is a significant advantage over the reported approach based on the gradual generation of **2** from **1** (Fig. 1a). We attempted to detect the generation of **2** by in-line IR and to compare in-line IR spectra with their predictions by DFT calculations. However, the elucidation of the reaction intermediate was difficult because the characteristic IR absorption of indole was easily changed due to the influence of the association state^51^. We also tried to detect the generation of **2** by reactions in an NMR tube; however, the efforts were futile by its instability (details, see Supplementary Information page S18–S22).

In conclusion, we examined all the previously reported syntheses of (1*H*-indol-3-yl)methyl halide **6**. We could not reproduce the reported syntheses and revised several reported structures of indole analogues. To develop a reliable and practical synthetic approach for nucleophilic substitutions at the α-position of the indole ring, we examined the rapid (0.02 s) and mild (25 °C) generation of the highly reactive (1*H*-indol-3-yl)methyl electrophile that enabled the rapid (0.1 s) and mild (25 °C) nucleophilic substitution. Eighteen unprotected indole analogues were successfully synthesized using the developed microflow nucleophilic substitution with various *N*-, *S*-, and *C*-nucleophiles. Extending the residence time of the electrophile (from 0.02 to 0.5 s) dramatically decreased the yield from 77 to 20%. Moreover, comparable batch conditions resulted in a 0% yield. These results clearly indicate the instability of the highly active (1*H*-indol-3-yl)methyl electrophile. Microflow technology realized the in situ preparation and use of such a highly unstable species for nucleophilic substitutions. This study offers a solution for a general and important problem in nucleophilic substitution at the α-position of electron-rich aromatic rings.

## Methods

### General techniques

See Supplementary Information (page S3).

### Synthesis of indole analogues

See Supplementary Information (pages S5–S10).

### Optimization of nucleophilic substitution

See Supplementary Information (pages S10–S18).

### Typical procedure and compound characterization data

See Supplementary Information (pages S22–S32).

### NMR chart

See Supplementary Figures 11–56.

## Supplementary information

Supplementary Information

Description of Additional Supplementary Files

Supplementary Data

## Data Availability

The authors declare that the data supporting the findings of this study are available within the paper and its Supplementary Information files. All other data are available from the corresponding author upon reasonable request. The X-ray crystallographic coordinates for structure **19** reported in this study have been deposited at the Cambridge Crystallographic Data Centre (CCDC), under deposition number CCDC-2201060. The data can be obtained free of charge from The Cambridge Crystallographic Data Centre via www.ccdc.cam.ac.uk/data_request/cif (See Supplementary Data).
