# Supplementary material for: Verification of preparations of (1H-indol-3-yl)methyl electrophiles and development of their microflow rapid generation and substitution
Source: Commun Chem. 2023 Mar 4;6:47. doi: 10.1038/s42004-023-00837-1 (PMC9985609; doi:10.1038/s42004-023-00837-1)
Supplement: Supplementary file 1 — Supplementary Information [file 42004_2023_837_MOESM1_ESM.pdf]

Supplementary Information

Verification of preparations of (1*H*-indol-3-yl)methyl electrophiles and development of their micro-flow rapid generation and substitution

Hisashi Masui,* Sena Kanda, and Shinichiro Fuse*

Department of Basic Medicinal Sciences
Graduate School of Pharmaceutical Sciences, Nagoya University
Furo-cho, Chikusa-ku, Nagoya, 464-8601 Japan

E-mail: masui@ps.nagoya-u.ac.jp
fuse@ps.nagoya-u.ac.jp

Table of Contents

Supplementary Methods S3

1. General techniques S3

2. Micro-flow reactor setup S3

3. Synthesis of indole analogues using batch reactor S5

3.1. Procedure of the Borodin–Hunsdiecker reaction for the synthesis of **6a** S5

3.2. Procedure of the bromination of alcohol **7a** for the synthesis of **6a** S6

3.3. ^{1}H NMR spectrum of **7a** S7

3.4. Procedure for the synthesis of **14**, **17**, and **19** S7

4. Optimization of nucleophilic substitution using micro-flow reactor S10

4.1. Examination of solvents S10

4.2. Examination of activation reagents S11

4.3. Examination of reaction times for activation S13

4.4. Examination of amounts of PBr_3 S14

4.5. Examination of temperatures S15

4.6. Examination of bases S16

4.7. Examination of concentrations S17

5. Procedure for synthesis of azide **3b** using a batch reactor S18

6. Examination of the generation of **2** in NMR tube S18

7. In-line IR analysis of the generation of (1*H*-indol-3-yl)methyl electrophile S19

8. Typical procedure for a micro-flow nucleophilic substitution: method A S22

9. Typical procedure for a micro-flow nucleophilic substitution: method B S23

10. NMR chart S33

Supplementary References S57

Supplementary Methods

1. General techniques

NMR spectra were recorded on a JEOL-ECS400 (400 MHz for ^{1}H, 100 MHz for ^{13}C) or JEOL-ECZ400 (400 MHz for ^{1}H, 100 MHz for ^{13}C, 376 MHz for ^{19}F) instrument in the indicated solvent. Chemical shifts were reported in units of parts per million (ppm) relative to the signal (0.00 ppm) for internal tetramethylsilane for solutions in $CDCl_3$ (7.26 ppm for ^{1}H, 77.1 ppm for ^{13}C) or DMSO-d_6 (2.50 ppm for ^{1}H, 39.5 ppm for ^{13}C). Multiplicities were reported by using the following abbreviations: s; singlet, d; doublet, t; triplet, m; multiplet, br; broad, *J*; coupling constants in Hertz (Hz). Infrared (IR) spectra were recorded on a JASCO FT/IR-4100 Fourier Transform Infrared Spectrophotometer. Only the strongest and/or structurally important peaks were reported as the IR data given in cm^{-1}. High resolution mass spectra (HRMS) were obtained on a Bruker Daltonics Compact in electrospray ionization (ESI) method. Column chromatography was performed on Silica Gel PSQ 60B purchased from Fuji Silysia Chemical LTD. Reactions were monitored by thin-layer chromatography carried out on 0.25 mm E. Merck silica gel plates (60F-254) with UV light, visualized by *p*-anisaldehyde, ceric sulfate solution, ninhydrin solution, 10% ethanolic phosphomolybdic acid. THF was dried by a Glass Contour Solvent dispensing system (Nikko Hansen & Co., Ltd.). CH_3CN was dried by molecular sieves 4A. Indole-3-methanol (**7a**) was purchased from TCI (Japan).

Micro-flow reactor setup

Stainless steel V-shape mixer was purchased from Sanko Seiki Co., Ltd. (inner diameter: 0.250 mm). The front and side view of V-shape mixer is shown in **Supplementary Figure 1**. Teflon® tubes (inner diameter: 0.800 or 0.500 or 0.250 mm) were purchased from Senshu Scientific Co., Ltd. PEEK fittings, PEEK unions, stainless steel tubes, stainless steel fittings, and stainless steel unions (inner diameter: 0.800 mm) were purchased from GL Science Inc. Solutions were introduced to a micro-flow system with syringe pumps (Harvard PHD ULTRA) equipped gastight syringes (SGE 10 mL). The gastight syringes and the Teflon tubes were connected with joints purchased from Flon Industry Co., Ltd. In-line IR analysis was performed using Mettler-Toledo React IR™ 15 Micro-flow cell.

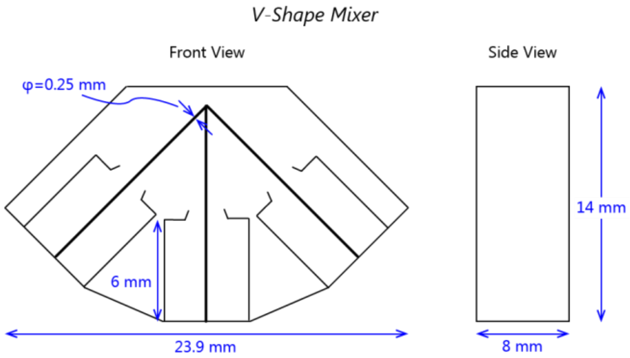

Supplementary Figure 1. V-shape mixer used in this study.

The employed micro-flow system was shown in **Supplementary Figure 2**. The gastight syringes and the V-shape mixer 1 and the V-shape mixer 2 were connected with the Teflon® tubes and stainless steel tubes (for controlling the temperature of solutions). The V-shape mixer 1 and the V-shape mixer 2 were connected with the reaction tube 1 (Teflon® tube). The V-shape mixer 2 was connected with the reaction tube 2 (Teflon® tube). The mixers and reaction tubes were immersed in water bath. The anhydrous solvent used in the reaction was passed through the reaction tube preliminary.

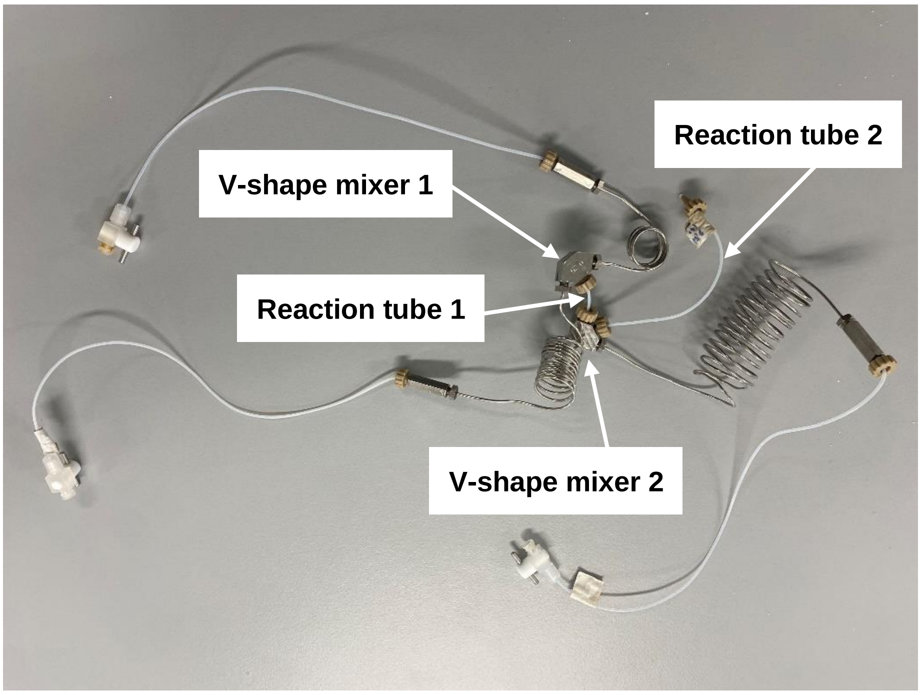

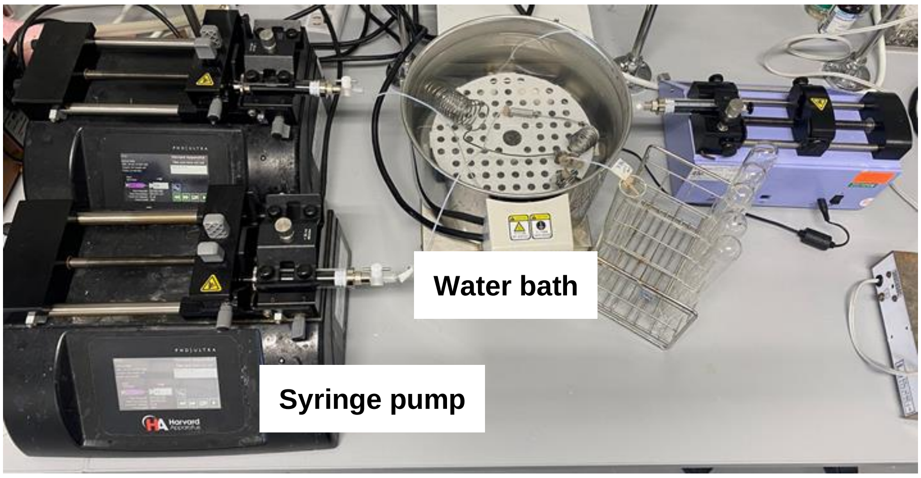

Supplementary Figure 2. Micro-flow reactor setup.

3. Synthesis of indole analogues using batch reactor

3.1. Procedure of the Borodin–Hunsdiecker reaction for the synthesis of 6a

1st examination based on the procedure reported by Eryshev et al.[1] except for work up method

To a solution of indole-3-acetic acid (**8**) (1.75 g, 10.0 mmol, 1.00 equiv.) in carbon tetrachloride (6.00 mL) and acetone (4.50 mL) was added portionwise red mercury (II) oxide (4.87 g, 22.5 mmol, 2.25 equiv.) under an argon atmosphere at room temperature in the dark. After being stirred at 55 °C for 10 min, to the reaction mixture was added dropwise bromine (0.773 mL, 15.0 mmol, 1.50 equiv.) over 30 min via syringe pump. After being stirred at 55 °C for 10 min, a highly lachrymatory compound and a dark red precipitate were generated. The precipitate was removed by filtration and the filtrate was diluted with Et_2O. Then, a dark red precipitate was generated again. The precipitate was removed by filtration and the filtrate was concentrated *in vacuo* without aqueous work up. The obtained residue was analyzed by ^{1}H NMR spectroscopy, however, the desired alkyl bromide was not detected.

2nd examination based on the procedure reported by Eryshev et al.[1]

To a solution of indole-3-acetic acid (**8**) (350 mg, 2.00 mmol, 1.00 equiv.) in carbon tetrachloride (1.00 mL) and acetone (0.900 mL) was added portionwise red mercury (II) oxide (975 mg, 4.50 mmol, 2.25 equiv.) under an argon atmosphere at room temperature in the dark. After being stirred at 55 °C for 10 min, to the reaction mixture was added bromine (0.155 mL, 3.00 mmol, 1.50 equiv.) dropwise over 30 min via syringe. After being stirred at 55 °C for 10 min, a highly lachrymatory compound and a dark red precipitate were generated. The precipitate was removed by filtration and the filtrate was diluted with Et_2O. Then, a dark red precipitate was generated again. The precipitate was removed by filtration and the filtrate

was washed with water, 60% sodium bisulfite solution, and water. The organic layer was dried over Na_2SO_4, filtered, and concentrated *in vacuo*. The obtained residue was analyzed by ^1H NMR spectroscopy, however, the desired alkyl bromide was not detected.

3rd examination based on the modified procedure of Eryshev et al.[1]

To a solution of indole-3-acetic acid (**8**) (500 mg, 2.85 mmol, 1.00 equiv.) and potassium hydroxide (188 mg, 2.85 mmol, 1.00 equiv.) in water (8.55 mL) was added a solution of silver (I) nitrate (485 mg, 1.98 mmol, 1.00 equiv.) in water (5.70 mL) at room temperature. After being stirred at the same temperature for 15 min, the precipitate was collected by filtration, washed with water, and dried *in vacuo*. The obtained silver salt was used for the next reaction without further purification.

To a suspension of the silver salt (100 mg, 0.350 mmol, 1.00 equiv.) in carbon tetrachloride (2.00 mL) and acetone (1.50 mL) was added dropwise bromine (27.4 μL, 0.530 mmol, 1.50 equiv.) over 30 min via syringe under an argon atmosphere at room temperature. After being stirred at 55 °C for 10 min, a dark red precipitate was generated. The precipitate was removed by filtration and the filtrate was washed with 10% $NaHCO_3$ aq. solution, dry over Na_2SO_4, filtered, and concentrated *in vacuo*. The obtained residue was analyzed by ^1H NMR spectroscopy, however, the desired alkyl bromide was not detected and indole-3-acetic acid (**8**) was detected.

4th examination based on the modified procedure of Eryshev et al.[1]

To a suspension of the above-mentioned silver salt (100 mg, 0.350 mmol, 1.00 equiv.) in carbon tetrachloride (2.45 mL) was added dropwise bromine (33.0 μL, 0.640 mmol, 1.83 equiv.) over 30 min via syringe under an argon atmosphere at room temperature. After being stirred at reflux temperature for 1 h, a dark red precipitate was generated. The precipitate was removed by filtration and the filtrate was washed with 10% $NaHCO_3$ aq. solution, dry over Na_2SO_4, filtered, and concentrated *in vacuo*. The obtained residue was analyzed by ^1H NMR spectroscopy, however, the desired alkyl bromide was not detected.

3.2. Procedure of the bromination of alcohol 7a for the synthesis of 6a

1st to 3rd examinations based on the procedure reported by Scanlan et al.[2]

To a suspension of lithium bromide (347 mg, 4.00 mmol, 2.00 equiv.) in CH_3CN (5.00 mL) were added TMSCl (0.635 mL, 5.00 mmol, 2.50 equiv.) and a solution of indole-3-methanol (**7a**) (294 mg, 2.00 mmol, 1.00 equiv.) in CH_3CN (5.00 mL) under an argon atmosphere at room temperature. After being stirred at reflux temperature for 2 h, a large amount of a dark red precipitate was generated. The precipitate was removed by filtration and the filtrate was

concentrated *in vacuo*. The crude red oil was obtained (1st exam: 14.5 mg; 2nd exam: 18.6 mg; 3rd exam: 32.0 mg; It should be noted that amounts of obtained crude oil were rather small probably due to generation of substantial amount of polymers). The obtained residues were analyzed by ^{1}H NMR spectroscopy, however, the desired alkyl bromide was not detected.

4th examination based on the modified procedure of Scanlan et al.[2]
To a solution of TMSBr (48.5 μL, 0.374 mmol, 1.10 equiv.) in CH_3CN (3.40 mL) was added a solution of indole-3-methanol (**7a**) (50.0 mg, 0.340 mmol, 1.00 equiv.) in CH_3CN (3.40 mL) under an argon atmosphere at room temperature. After being stirred at reflux temperature for 2 h, a dark red precipitate was generated. The precipitate was removed by filtration and the filtrate was concentrated *in vacuo*. The obtained residue was analyzed by ^{1}H NMR spectroscopy, however, the desired alkyl bromide was not detected.

3.3. ^{1}H NMR spectrum of 7a

^{1}H NMR spectrum reported by Babu G et al.[3]
^{1}H NMR (400 MHz, $CDCl_3$): δ 7.75-7.16 (m, 3H), 7.62 (d, 1H), 6.74 (s, 1H), 4.58 (s, 2H).

^{1}H NMR spectrum of other previous report.[4]
^{1}H NMR (300 MHz, $CDCl_3$): δ 8.14 (brs, 1H), 7.77 (d, *J* = 8.1 Hz, 1H), 7.41 (d, *J* = 7.8 Hz, 1H), 7.29-7.16 (m, 3H), 4.92 (d, *J* = 3.9 Hz, 2H).

Our observed ^{1}H NMR spectrum of purchased alcohol **7a**
^{1}H NMR (400 MHz, $CDCl_3$): δ 8.09 (brs, 1H), 7.75 (d, *J* = 8.0 Hz, 1H), 7.39 (d, *J* = 7.6 Hz, 1H), 7.24-7.16 (m, 3H), 4.90 (d, *J* = 5.2 Hz, 2H), 1.45-1.43 (m, 1H).

Our observed ^{1}H NMR spectrum of purchased alcohol **7a** was not consistent with that reported by Babu G et al.[3], whereas was well consistent with that in other previous report.[4]

3.4. Procedure for the synthesis of 14, 17, and 19

1-(7-Chloro-1*H*-indol-3-yl)-*N,N*-dimethylmethanamine (14)

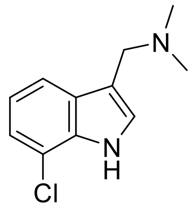

To a solution of 7-chloro-1*H*-indole (**11**) (200 mg, 1.32 mmol, 1.00 equiv.) in dichloromethane (3.96 mL) was added *N,N*-dimethylmethyleneiminium chloride (**12**) (185 mg, 1.98 mmol, 1.50 equiv.) under an argon atmosphere at room temperature. After being stirred at the same temperature for 2 h, the reaction was quenched with 1 M NaOH aq. and the aqueous layer was extracted with two portions of ethyl acetate. The combined organic layer was washed with brine, dried over Na_2SO_4, filtered, and concentrated *in vacuo*. The residue was purified by column chromatography on silica gel (ethyl acetate) to give 1-(7-chloro-1*H*-indol-3-yl)-*N,N*-dimethylmethanamine (**14**) (232 mg, 1.11 mmol, 84%) as a white solid.

mp 127–132 °C, IR (neat): 2945, 2818, 2776, 1438, 1342, 1202, 1086, 784, 730 cm^{-1}; ^1H NMR (400 MHz, $CDCl_3$): δ 8.51 (brs, 1H), 7.60 (d, *J* = 7.8 Hz, 1H), 7.20 (s, 1H), 7.18 (d, *J* = 7.3 Hz, 1H), 7.06 (dd, *J* = 7.3, 7.8 Hz, 1H), 3.65 (s, 2H), 2.30 (s, 6H); ^{13}C NMR (100 MHz, $CDCl_3$): δ 133.4, 129.1, 126.9, 122.0, 121.2, 117.2, 53.1, 43.4; HRMS (ESI): calcd for $[C_{19}H_{20}FN_3+Na]^+$ 231.0665, found 231.0665.
Spectral data were well consistent with those in the previous report.[5]

3-Chloro-3-(chloromethyl)indolin-2-one (17)

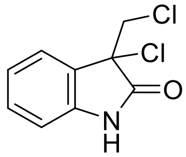

To a solution of indole-3-methanol (**7a**) (29.4 mg, 0.200 mmol, 1.00 equiv) in ethyl acetate (8.00 mL) was added 1,3-dichloro-5,5-dimethylhydantoin (**15**) (43.3 mg, 0.220 mmol, 1.10 equiv) under an argon atmosphere at room temperature. After being stirred at the same temperature for 10 min, the reaction mixture was concentrated *in vacuo*. The residue was purified by column chromatography (hexane : ethyl acetate = 3 : 1) to give 3-chloro-3-(chloromethyl)indolin-2-one (**17**) (11.4 mg, 0.0480 mmol, 24%) as a brown solid.

mp 128–130 °C, IR (neat): 3207, 1731, 1619, 1472, 1331, 1220, 1178, 749, 676 cm^{-1}; ^1H NMR (400 MHz, $CDCl_3$): δ 8.14 (d, *J* = 7.8 Hz, 1H), 7.43 (d, *J* = 7.8 Hz, 1H), 7.37-7.33 (m, 1H), 7.17-7.14 (m, 1H), 6.95 (d, *J* = 7.8 Hz, 1H), 4.17 (d, *J* = 11.0 Hz, 1H), 4.07 (d, *J* = 11.0 Hz, 1H); ^{13}C NMR (100 MHz, $CDCl_3$): δ 173.6, 140.8, 131.2, 127.1, 124.9, 123.9, 111.0, 63.2, 46.0; HRMS (ESI): calcd for $[C_9H_7Cl_2NO+Na]^+$ 237.9797, found 237.9804.

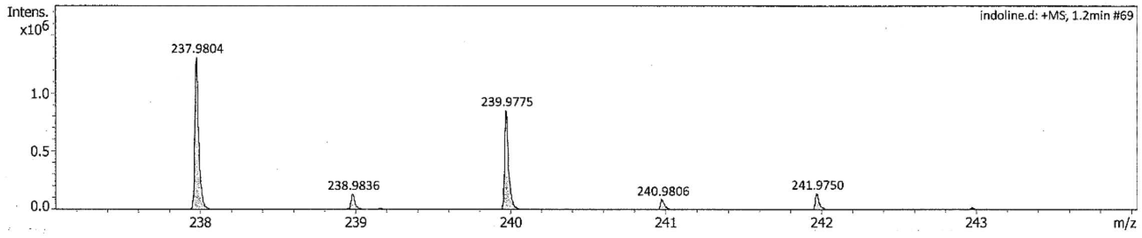

Supplementary Figure 3. HRMS chart of **17**.

1-(4-Bromobenzoyl)-3-chloro-3-(chloromethyl)indolin-2-one (19)

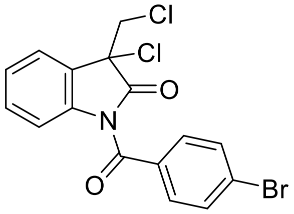

To a solution of 3-chloro-3-(chloromethyl)indolin-2-one (**17**) (43.2 mg, 0.200 mmol, 1.00 equiv.) in dichloromethane (2.00 mL) were added NEt_3 (55.8 μL, 0.400 mmol, 2.00 equiv.) and *p*-bromobenzoyl chloride (**18**) (27.3 μL, 0.200 mmol, 1.00 equiv.) under an argon atmosphere at room temperature. After being stirred at the same temperature for 17 h, the reaction was quenched with sat. $NaHCO_3$ aq. and the aqueous layer was extracted with three portions of dichloromethane. The combined organic layer was washed with brine, dried over Na_2SO_4, filtered, and concentrated *in vacuo*. The residue was purified by PTLC (hexane : ethyl acetate = 10 : 1) to give 1-(4-bromobenzoyl)-3-chloro-3-(chloromethyl)indolin-2-one (**19**) (45.4 mg, 0.108 mmol, 54%) as a white amorphous solid.

mp 153–155 °C, IR (neat): 1770, 1698, 1588, 1468, 1339, 1287, 1159, 756 cm^{-1}; ^1H NMR (400 MHz, $CDCl_3$): δ 7.88 (d, *J* = 7.8 Hz, 1H), 7.65 (s, 4H), 7.53-7.49 (m, 2H), 7.37-7.33 (m, 1H), 4.24 (d, *J* = 11.0 Hz, 1H), 4.13 (d, *J* = 11.0 Hz, 1H); ^{13}C NMR (100 MHz, $CDCl_3$): δ 171.3, 167.7, 140.3, 132.2, 131.9, 131.7, 131.1, 128.7, 126.2, 125.9, 124.4, 115.6, 63.3, 46.0; HRMS (ESI): calcd for $[C_{16}H_{10}BrCl_2NO_2+Na]^+$ 419.9164, found 419.9174.

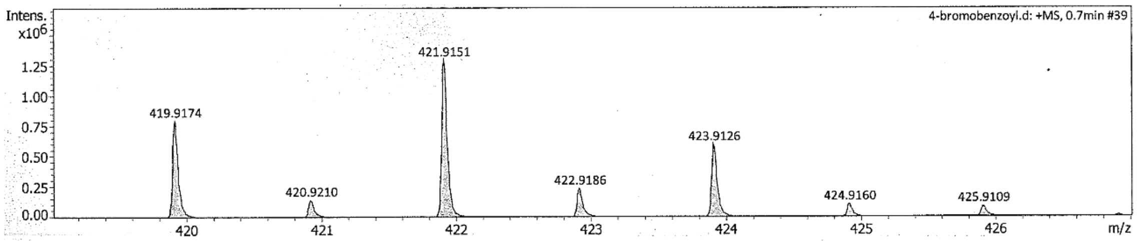

Supplementary Figure 4. HRMS chart of **19**.

Single X-ray diffraction data were collected on a Rigaku R-AXIS Rapid diffractometer with graphite monochromatized Cu-Ka radiation (λ = 1.54187 Å). Empirical formula: $C_{16}H_{10}BrCl_2NO_2$, Formula weight: 399.07, Temperature: -150.0 °C, Crystal color: colorless, Crystal habit: platelet, Crystal size: 0.300 x 0.300 x 0.100 mm^3, Crystal system: triclinic, Space group: P-1 (#2), Unit cell dimensions: a = 9.2717(5) Å, b = 11.0960(6) Å, c = 14.8766(8) Å, Volume: 1478.08(14) $Å^3$, Z value: 4, Density (calculated): 1.793 g/cm^3, Absorption coefficient: 71.762 cm^{-1}, F(000): 792.00, Reflections collected: 14842, Independent reflections: 5219 [R(int) = 0.1027, Max. and min. transmission: 0.488 and 0.135, Goodness-of-fit on F^2: 1.007, R indices [I>2sigma(I)]: 0.0630, R indices (all data): R = 0.0803, wR_2 = 0.1768, Largest diff. peak and hole: 1.02 and -1.72 e.$Å^{-3}$. CCDC-2201060 contains the supplementary crystallographic data for this paper. These data can be obtained free of charge from The Cambridge Crystallographic Data Centre via www.ccdc.cam.ac.uk/data_request/cif.

4. Optimization of nucleophilic substitution using micro-flow reactor

4.1. Examination of solvents

Table S-1. Examination of solvents in micro-flow nucleophilic substitution.

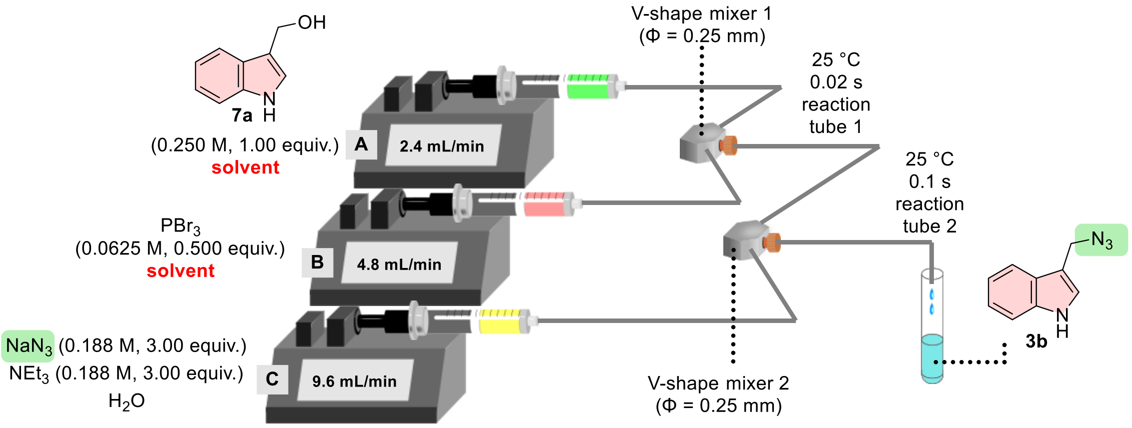

entry	solvent	yield [%][a]	
		**3b**	**7a**
1	DMSO	11	27
2	DMF	29	8
3	acetone	73	5
4	THF	75	trace
**5**	**CH_3CN**	**77**	**trace**

[a] Yields were determined by ^{1}H NMR analysis using 1,1,2-trichloroethane as an internal standard.

A solution of indole-3-methanol (**7a**) (0.250 M, 1.00 equiv.) in **solvent** (flow rate: 2.40 mL/min) and a solution of PBr_3 (0.0625 M, 0.500 equiv.) in **solvent** (flow rate: 4.80 mL/min) were introduced to V-shape mixer 1 at 25 °C with the syringe pumps. The resultant mixture was passed through reaction tube 1 (inner diameter: 0.250 mm, length: 48.9 mm, volume: 2.40 μL, reaction time: 0.02 s) at 25 °C. The resultant mixture and a solution of NaN_3 (0.188 M, 3.00 equiv.) and NEt_3 (0.188 M, 3.00 equiv.) in water (flow rate: 9.60 mL/min) were introduced to V-shape mixer 2 at 25 °C with the syringe pumps. The resultant mixture was passed through the reaction tube 2 (inner diameter: 0.500 mm, length: 143 mm, volume: 28.0 μL, reaction time: 0.1 s) at 25 °C. After being eluted for *ca.* 10 s to reach a steady state, the resultant mixture was poured into a test tube containing sat. $NaHCO_3$ aq. (5 mL) for 45 s at room temperature. The reaction mixture was extracted with a solution of dichloromethane and methanol (10 : 1) (5 mL) three times. The organic layer was washed with brine (20 mL), dried over Na_2SO_4, filtered, and evaporated *in vacuo*. Yields were determined by ^{1}H NMR analysis using 1,1,2-trichloroethane as an internal standard.

4.2. Examination of activation reagents

Table S-2. Examination of activation reagents in micro-flow nucleophilic substitution.

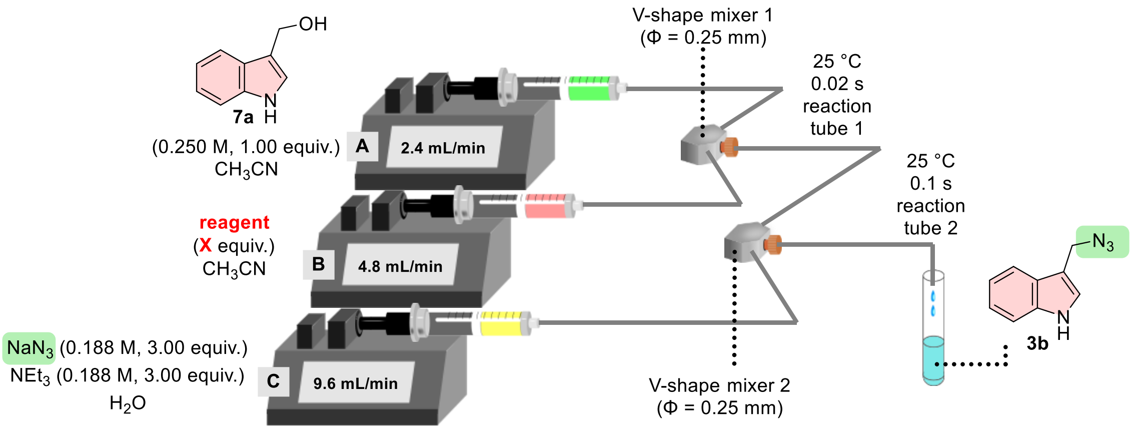

entry	reagent	X [equiv.][a]	yield [%][b] 3b	7a
**1**	**PBr_3**	**0.500**	**77**	**trace**
2	PCl_3	0.500	52	4
3	$POCl_3$	0.500	0	100
4[c]	AcBr	1.50	4	61
5[c]	AcCl	1.50	0	100

6[c]	Ac_2O	1.50	0	67
7[c]	Tf_2O	1.50	8	38
8[c]	MsCl	1.50	29	41
9[c]	TsCl	1.50	0	0
10	$SOBr_2$	0.750	7	23
11	$SOCl_2$	0.750	15	16

[a] The amount of reagents were changed base on reaction mechanisms. Theoretically, 1 equiv. of PBr_3, PCl_3, and $POCl_3$ can convert 3 equiv. of the alcohol to the alkyl halide, 1 equiv. of $SOBr_2$ and $SOCl_2$ can convert 2 equiv. of the alcohol, and 1 equiv. of Tf_2O, MsCl, TsCl, AcBr, AcCl, and Ac_2O can convert equimolar amount of alcohol. [b] Yields were determined by ^1H NMR analysis using 1,1,2-trichloroethane as an internal standard. [c] NEt_3 was injected via the syringe pump A instead of via the syringe pump C.

A solution of indole-3-methanol (**7a**) (0.250 M, 1.00 equiv.) in CH_3CN (flow rate: 2.40 mL/min) and a solution of **reagent** (**X** equiv.) in CH_3CN (flow rate: 4.80 mL/min) were introduced to V-shape mixer 1 at 25 °C with the syringe pumps. The resultant mixture was passed through reaction tube 1 (inner diameter: 0.250 mm, length: 48.9 mm, volume: 2.40 μL, reaction time: 0.02 s) at 25 °C. The resultant mixture and a solution of NaN_3 (0.188 M, 3.00 equiv.) and NEt_3 (0.188 M, 3.00 equiv.) in water (flow rate: 9.60 mL/min) were introduced to V-shape mixer 2 at 25 °C with the syringe pumps. The resultant mixture was passed through the reaction tube 2 (inner diameter: 0.500 mm, length: 143 mm, volume: 28.0 μL, reaction time: 0.1 s) at 25 °C. After being eluted for *ca*. 10 s to reach a steady state, the resultant mixture was poured into a test tube containing sat. $NaHCO_3$ aq. (5 mL) for 45 s at room temperature. The reaction mixture was extracted with a solution of dichloromethane and methanol (10 : 1) (5 mL) three times. The organic layer was washed with brine (20 mL), dried over Na_2SO_4, filtered, and evaporated *in vacuo*. Yields were determined by ^1H NMR analysis using 1,1,2-trichloroethane as an internal standard.

4.3. Examination of reaction times for activation

Table S-3. Examination of reaction times for activation in micro-flow nucleophilic substitution.

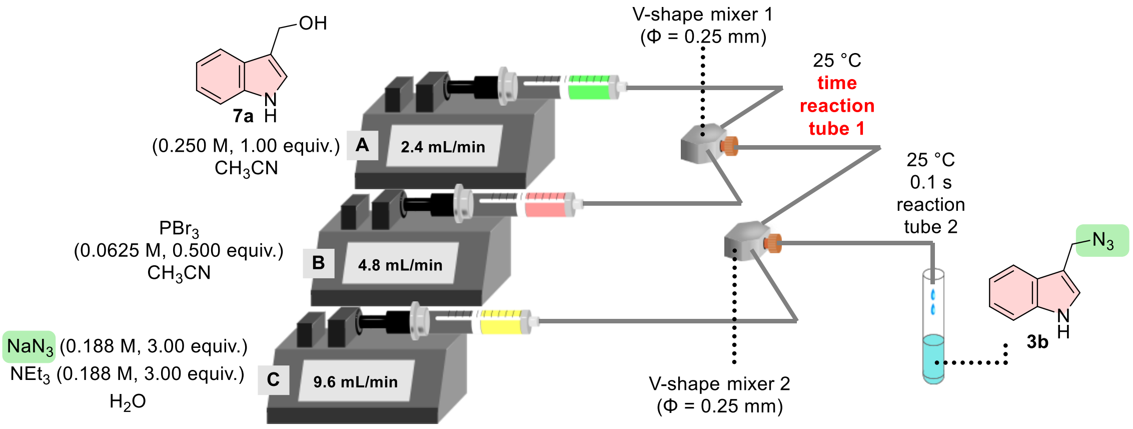

entry	time [s]	reaction tube 1 inner diameter (mm)	reaction tube 1 length (mm)	yield [%][a] 3b	yield [%][a] 7a
**1**	**0.02**	**0.250**[b]	**48.9**[b]	**77**	**trace**
2	0.05	0.250	122	60	0
3	0.1	0.500	61.1	54	0
4	0.5	0.500	306	20	0

[a] Yields were determined by ^{1}H NMR analysis using 1,1,2-trichloroethane as an internal standard. [b] This is the minimum inner diameter and length for connecting V-shape mixer 1 and V-shape mixer 2 for carrying out an experiment.

A solution of indole-3-methanol (**7a**) (0.250 M, 1.00 equiv.) in CH_3CN (flow rate: 2.40 mL/min) and a solution of PBr_3 (0.0625 M, 0.500 equiv.) in CH_3CN (flow rate: 4.80 mL/min) were introduced to V-shape mixer 1 at 25 °C with the syringe pumps. The resultant mixture was passed through **reaction tube 1** at 25 °C. The resultant mixture and a solution of NaN_3 (0.188 M, 3.00 equiv.) and NEt_3 (0.188 M, 3.00 equiv.) in water (flow rate: 9.60 mL/min) were introduced to V-shape mixer 2 at 25 °C with the syringe pumps. The resultant mixture was passed through the reaction tube 2 (inner diameter: 0.500 mm, length: 143 mm, volume: 28.0 μL, reaction time: 0.1 s) at 25 °C. After being eluted for *ca*. 10 s to reach a steady state, the resultant mixture was poured into a test tube containing sat. $NaHCO_3$ aq. (5 mL) for 45 s at room temperature. The reaction mixture was extracted with a solution of dichloromethane and methanol (10 : 1) (5 mL) three times. The organic layer was washed with brine (20 mL), dried over Na_2SO_4, filtered, and evaporated *in vacuo*. Yields were determined by ^{1}H NMR

analysis using 1,1,2-trichloroethane as an internal standard.

4.4. Examination of amounts of PBr_3

Table S-4. Examination of amounts of PBr_3 in micro-flow nucleophilic substitution.

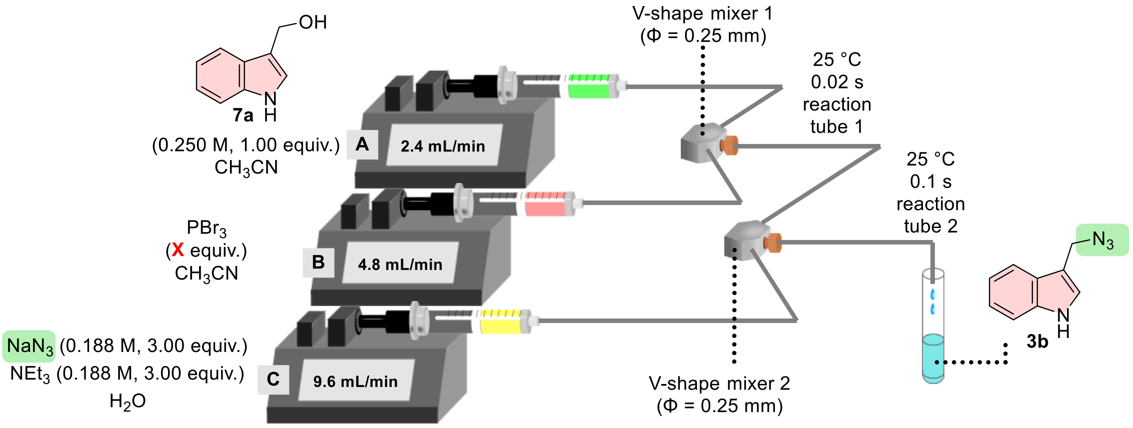

entry	X [equiv.]	yield [%][a] 3b	7a
1	0.500	77	trace
**2**	**0.350**	**84**	**0**

[a] Yields were determined by ^1H NMR analysis using 1,1,2-trichloroethane as an internal standard.

A solution of indole-3-methanol (**7a**) (0.250 M, 1.00 equiv.) in CH_3CN (flow rate: 2.40 mL/min) and a solution of PBr_3 (**X** equiv.) in CH_3CN (flow rate: 4.80 mL/min) were introduced to V-shape mixer 1 at 25 °C with the syringe pumps. The resultant mixture was passed through reaction tube 1 (inner diameter: 0.250 mm, length: 48.9 mm, volume: 2.40 μL, reaction time: 0.02 s) at 25 °C. The resultant mixture and a solution of NaN_3 (0.188 M, 3.00 equiv.) and NEt_3 (0.188 M, 3.00 equiv.) in water (flow rate: 9.60 mL/min) were introduced to V-shape mixer 2 at 25 °C with the syringe pumps. The resultant mixture was passed through the reaction tube 2 (inner diameter: 0.500 mm, length: 143 mm, volume: 28.0 μL, reaction time: 0.1 s) at 25 °C. After being eluted for *ca*. 10 s to reach a steady state, the resultant mixture was poured into a test tube containing sat. $NaHCO_3$ aq. (5 mL) for 45 s at room temperature. The reaction mixture was extracted with a solution of dichloromethane and methanol (10 : 1) (5 mL) three times. The organic layer was washed with brine (20 mL), dried over Na_2SO_4, filtered, and evaporated *in vacuo*. Yields were determined by ^1H NMR analysis using 1,1,2-trichloroethane as an internal standard.

4.5. Examination of temperatures

Table S-5. Examination of temperatures in micro-flow nucleophilic substitution.

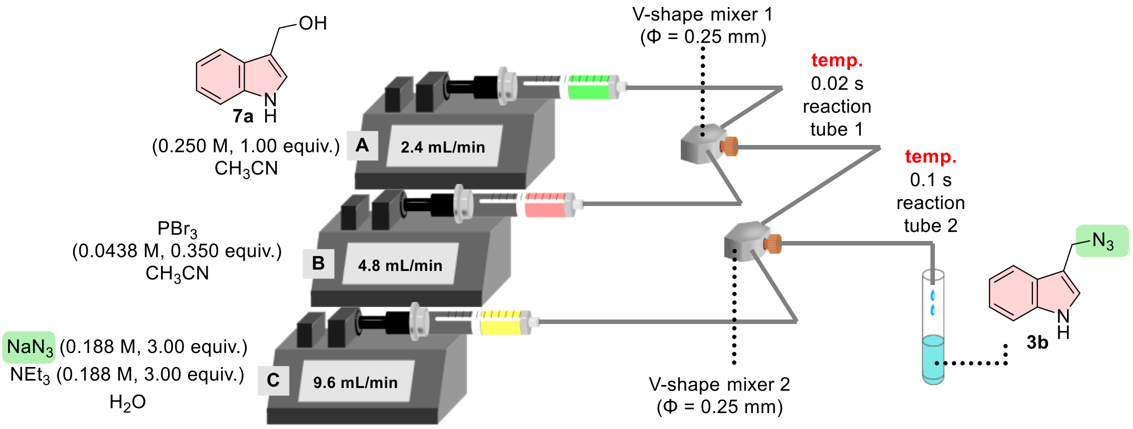

entry	temp. Y [°C]	yield [%][a] 3b	7a
1	0	74	0
**2**	**25**	**84**	**0**
3	40	clogged	clogged

[a] Yields were determined by ^{1}H NMR analysis using 1,1,2-trichloroethane as an internal standard.

A solution of indole-3-methanol (**7a**) (0.250 M, 1.00 equiv.) in CH_3CN (flow rate: 2.40 mL/min) and a solution of PBr_3 (0.0438 M, 0.350 equiv.) in CH_3CN (flow rate: 4.80 mL/min) were introduced to V-shape mixer 1 at **Y** °C with the syringe pumps. The resultant mixture was passed through reaction tube 1 (inner diameter: 0.250 mm, length: 48.9 mm, volume: 2.40 μL, reaction time: 0.02 s) at **Y** °C. The resultant mixture and a solution of NaN_3 (0.188 M, 3.00 equiv.) and NEt_3 (0.188 M, 3.00 equiv.) in water (flow rate: 9.60 mL/min) were introduced to V-shape mixer 2 at **Y** °C with the syringe pumps. The resultant mixture was passed through the reaction tube 2 (inner diameter: 0.500 mm, length: 143 mm, volume: 28.0 μL, reaction time: 0.1 s) at **Y** °C. After being eluted for *ca*. 10 s to reach a steady state, the resultant mixture was poured into a test tube containing sat. $NaHCO_3$ aq. (5 mL) for 45 s at room temperature. The reaction mixture was extracted with a solution of dichloromethane and methanol (10 : 1) (5 mL) three times. The organic layer was washed with brine (20 mL), dried over Na_2SO_4, filtered, and evaporated *in vacuo*. Yields were determined by ^{1}H NMR analysis using 1,1,2-trichloroethane as an internal standard.

4.6. Examination of bases

Table S-6. Examination of bases in micro-flow nucleophilic substitution.

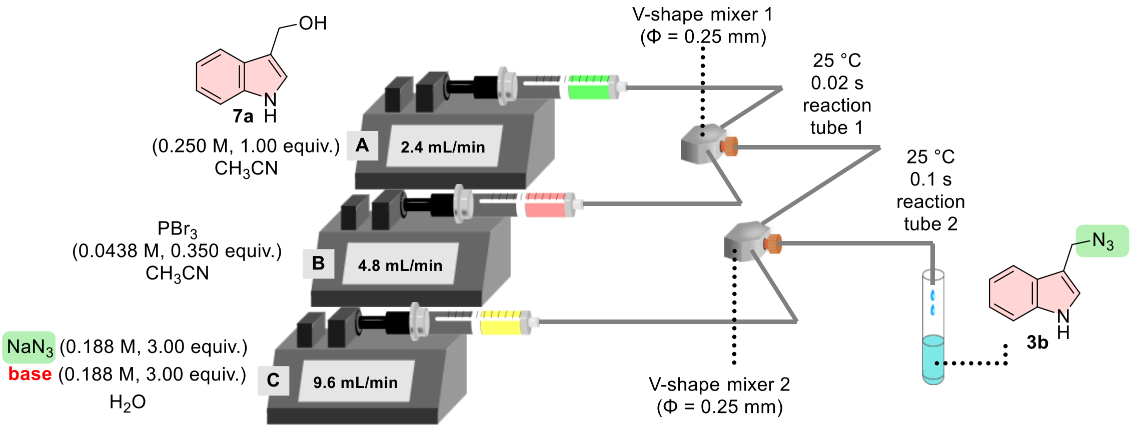

entry	**base** (pK_aH[a])	yield [%][b]	
		**3b**	**7a**
1	NaOH (15.7)	43	4
2	DBU (12.0)	2	1
**3**	**NEt_3 (10.8)**	**84**	**0**
4	DMAP (9.7)	0	0
5	DMBA (8.9)	4	50
6	NMM (7.4)	45	0
7	NMI (7.0)	41	0
8	2,6-lutidine (6.7)	84	0
9	pyridine (5.2)	73	2
10	NEt_3 + pyridine	57	4

[a] The pK_a of conjugated acids in H_2O. [b] Yields were determined by ^1H NMR analysis using 1,1,2-trichloroethane as an internal standard.

A solution of indole-3-methanol (**7a**) (0.250 M, 1.00 equiv.) in CH_3CN (flow rate: 2.40 mL/min) and a solution of PBr_3 (0.0438 M, 0.350 equiv.) in CH_3CN (flow rate: 4.80 mL/min) were

introduced to V-shape mixer 1 at 25 °C with the syringe pumps. The resultant mixture was passed through reaction tube 1 (inner diameter: 0.250 mm, length: 48.9 mm, volume: 2.40 μL, reaction time: 0.02 s) at 25 °C. The resultant mixture and a solution of NaN_3 (0.188 M, 3.00 equiv.) and **base** (0.188 M, 3.00 equiv.) in water (flow rate: 9.60 mL/min) were introduced to V-shape mixer 2 at 25 °C with the syringe pumps. The resultant mixture was passed through the reaction tube 2 (inner diameter: 0.500 mm, length: 143 mm, volume: 28.0 μL, reaction time: 0.1 s) at 25 °C. After being eluted for *ca*. 10 s to reach a steady state, the resultant mixture was poured into a test tube containing sat. $NaHCO_3$ aq. (5 mL) for 45 s at room temperature. The reaction mixture was extracted with a solution of dichloromethane and methanol (10 : 1) (5 mL) three times. The organic layer was washed with brine (20 mL), dried over Na_2SO_4, filtered, and evaporated *in vacuo*. Yields were determined by ^{1}H NMR analysis using 1,1,2-trichloroethane as an internal standard.

4.7. Examination of concentrations

Table S-7. Examination of concentrations in micro-flow nucleophilic substitution.

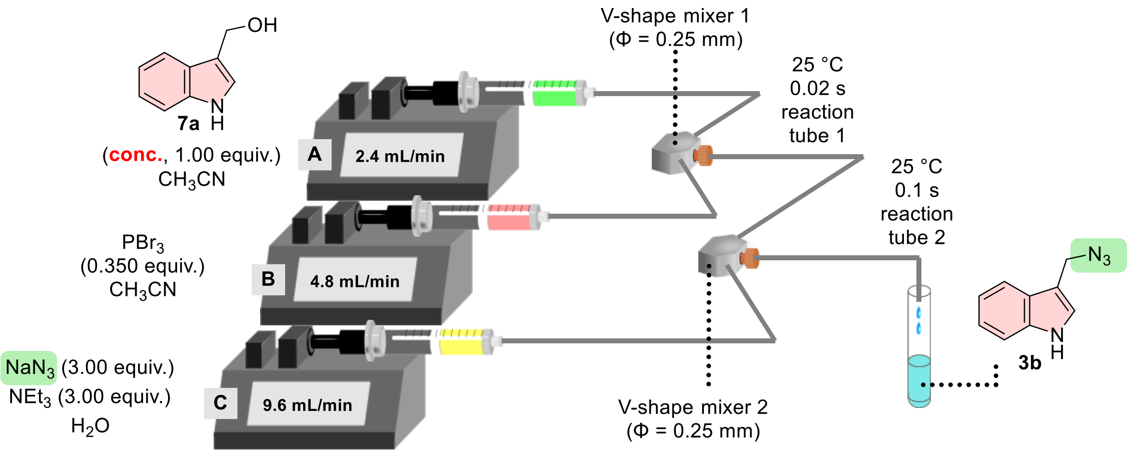

entry	conc. [M]	yield [%][a] 3b	7a
1	0.250	84	0
**2**	**0.0500**	**91, 94, 95**[b]	**0**

[a] Yields were determined by ^{1}H NMR analysis using 1,1,2-trichloroethane as an internal standard. [b] Three independent experiments were caried out.

A solution of indole-3-methanol (**7a**) (1.00 equiv.) in CH_3CN (flow rate: 2.40 mL/min) and a solution of PBr_3 (0.350 equiv.) in CH_3CN (flow rate: 4.80 mL/min) were introduced to V-shape

mixer 1 at 25 °C with the syringe pumps. The resultant mixture was passed through reaction tube 1 (inner diameter: 0.250 mm, length: 48.9 mm, volume: 2.40 μL, reaction time: 0.02 s) at 25 °C. The resultant mixture and a solution of NaN_3 (3.00 equiv.) and NEt_3 (3.00 equiv.) in water (flow rate: 9.60 mL/min) were introduced to V-shape mixer 2 at 25 °C with the syringe pumps. The resultant mixture was passed through the reaction tube 2 (inner diameter: 0.500 mm, length: 143 mm, volume: 28.0 μL, reaction time: 0.1 s) at 25 °C. After being eluted for *ca*. 10 s to reach a steady state, the resultant mixture was poured into a test tube containing sat. $NaHCO_3$ aq. (5 mL) for 45 s at room temperature. The reaction mixture was extracted with a solution of dichloromethane and methanol (10 : 1) (5 mL) three times. The organic layer was washed with brine (20 mL), dried over Na_2SO_4, filtered, and evaporated *in vacuo*. Yields were determined by ^1H NMR analysis using 1,1,2-trichloroethane as an internal standard.

5. Procedure for synthesis of azide 3b using a batch reactor

(Quantities of compounds, solvents and temperature were identical to those of flow condition.) To a vigorously stirred (magnetic stirrer, 1,000 rpm) solution of indole-3-methanol (**7a**) (0.0500 M, 1.00 equiv.) in CH_3CN (1.20 mL), a solution of PBr_3 (0.00875 M, 0.350 equiv.) in CH_3CN (2.40 mL) was added in one portion at 25 °C under argon atmosphere (*caution*: HBr evolved). After being stirred for 10 s at the same temperature, resultant mixture was added in one portion at 25 °C to a vigorously stirred (magnetic stirrer, 1,000 rpm) solution of NaN_3 (0.0375 M, 3.00 equiv.) and NEt_3 (0.0375 M, 3.00 equiv.) in water (4.80 mL). After being stirred for 10 s at the same temperature, sat. $NaHCO_3$ aq. (5 mL) was added in one portion at 25 °C (Under the flow conditions, nucleophilic substitution was performed in 0.02 and 0.1 s. However, under the batch conditions, it was impossible to operate the reaction within 0.5 s. Thus, the reaction time was extended to 10 s).

The reaction mixture was extracted with ethyl acetate (5 mL) three times. The organic layer was washed with brine (20 mL), dried over Na_2SO_4, filtered, and evaporated *in vacuo*. Yields were determined by ^1H NMR analysis using 1,1,2-trichloroethane as an internal standard. Three independent experiments were carried out.

1st run: 0% yield, 2nd run: 0% yield, 3rd run: 0% yield.

6. Examination of the generation of 2 in NMR tube

To a solution of indole-3-methanol (**7a**) (5.20 mg, 0.0353 mmol, 1.00 equiv.) in CD_3CN (0.700 mL) was added PBr_3 (1.10 μL, 0.0124 mmol, 0.350 equiv.) at room temparature. The resultant mixture was immediately analyzed by ^1H NMR, and the measurement was completed in 6 min 14 s after the addition of PBr_3. However, a complex mixture of compounds was observed.

7. In-line IR analysis of the generation of (1*H*-indol-3-yl)methyl electrophile

The schematic figure of the in-line IR system was shown in **Supplementary Figure 5**. The gastight syringes and V-shape mixer were connected with Teflon tube (inner diameter: 0.8 mm, length: 300 mm, volume: 151 μL). V-shape mixer and React IR™ 15 Micro Flow Cell were connected with reaction tube (Teflon® tube, inner diameter: 0.25 mm, length: 48.9 mm, volume: 2.40 μL).

First, in-line IR analysis of indole-3-methanol (**7a**) was performed. A solution of indole-3-methanol (**7a**) in CH_3CN (flow rate: 2.40 mL/min) and CH_3CN (flow rate: 4.80 mL/min) were introduced to V-shape mixer at 25 °C with the syringe pumps. The resultant solution of **7a** was passed through the reaction tube (inner diameter: 0.25 mm, length: 48.9 mm, volume: 2.40 μL) at 25 °C. After being eluted for *ca.* 10 s to reach a steady state, the solution of **7a** was analyzed by Mettler-Toledo React IR™ 15 Micro-flow cell.

Next, in-line IR analysis of the reaction mixture of **7a** and PBr_3 was performed. A solution of indole-3-methanol (**7a**) in CH_3CN (flow rate: 2.40 mL/min) and a solution of PBr_3 (0.350 equiv.) in CH_3CN (flow rate: 4.80 mL/min) were introduced to V-shape mixer at 25 °C with the syringe pumps. The resultant mixture was passed through a reaction tube (inner diameter: 0.25 mm, length: 48.9 mm, volume: 2.40 μL, reaction time: 0.02 s) at 25 °C. After being eluted for *ca.* 10 s to reach a steady state, the resultant mixture was analyzed by Mettler-Toledo React IR™ 15 Micro-flow cell.

In-line IR spectra (**Supplementary Figures 6** and **7**) were compared with predicted IR spectra of indole-3-methanol (**Supplementary Figure 8**), (1*H*-indol-3-yl)methyl bromide (**Supplementary Figure 9**), and 3-methylene-3*H*-indole (**Supplementary Figure 10**) by DFT calculations at the B3LYP/6-31G(d) level of theory using the Gaussian 16 program[6]. The observed IR spectrum of **7a** (**Supplementary Figure 6**) was not consistent with the predicted IR spectrum **7a** (**Supplementary Figure 8**) probably due to the association of indole. We could not assign observed IR absorbance of the reaction mixture of **7a** and PBr_3 (**Supplementary Figure 7**).

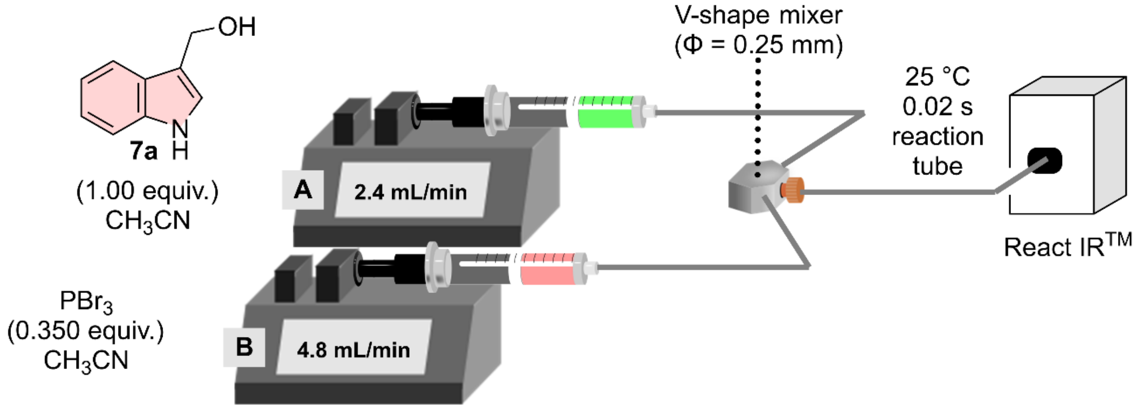

Supplementary Figure 5. Schematic figure of the in-line IR system.

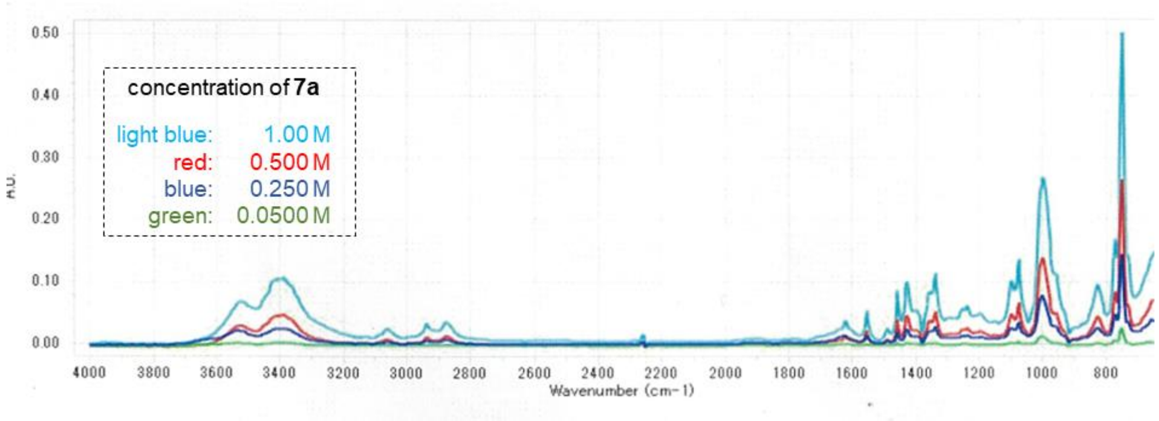

Supplementary Figure 6. In-line IR spectra of indole-3-methanol (**7a**).

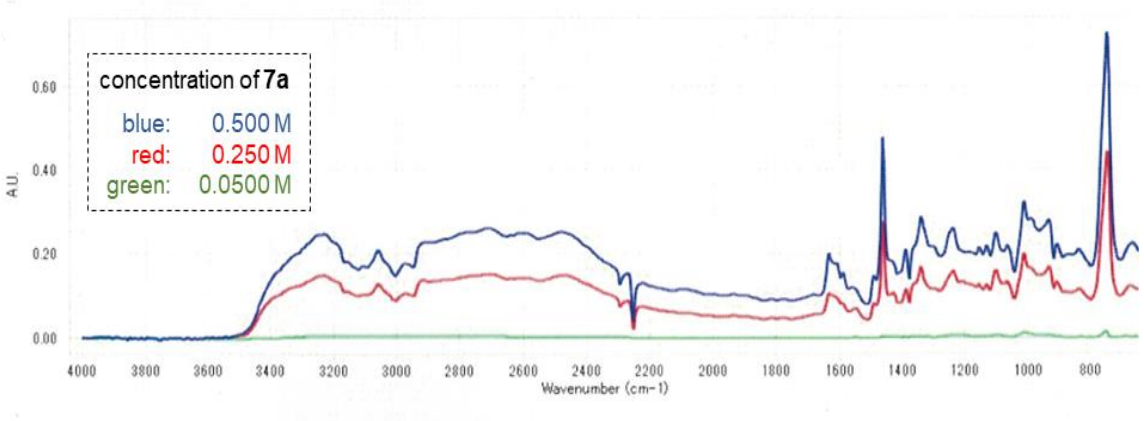

Supplementary Figure 7. In-line IR spectra of the reaction mixture of indole-3-methanol (**7a**) and PBr_3.

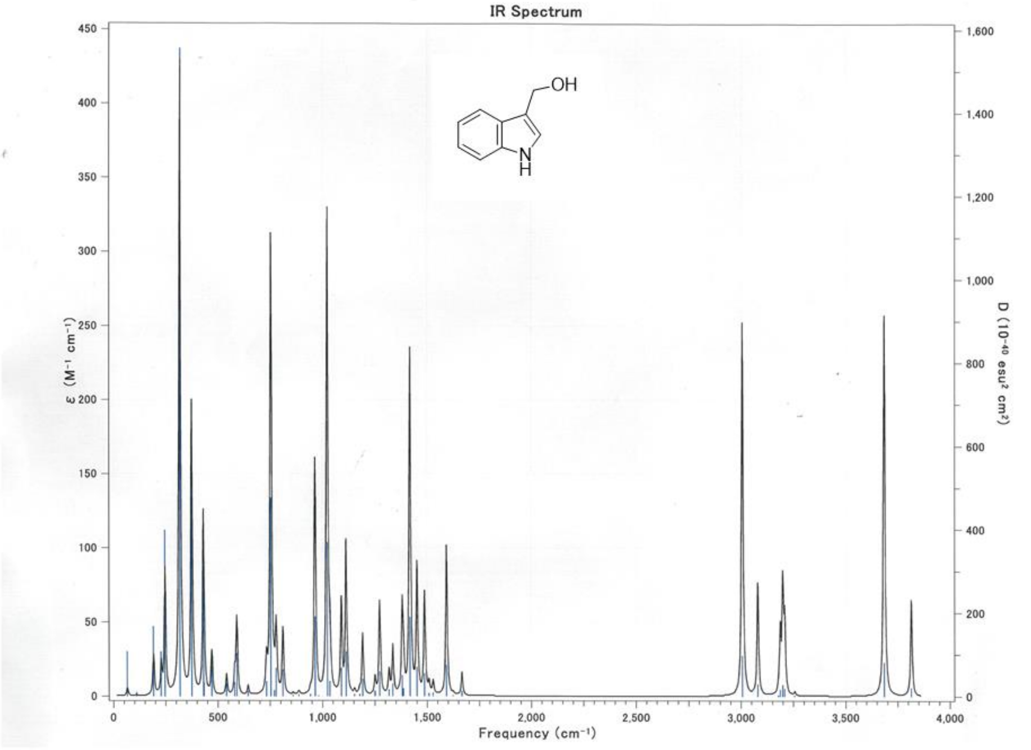

Supplementary Figure 8. Predicted IR spectra of indole-3-methanol (**7a**).

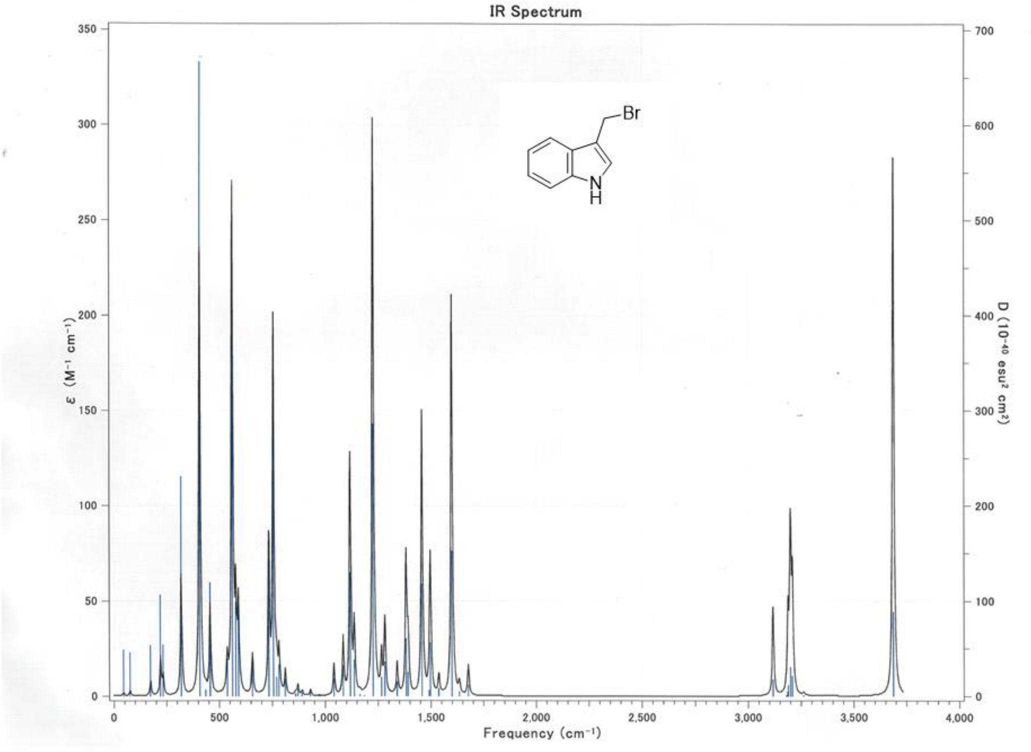

Supplementary Figure 9. Predicted IR spectra of (1*H*-indol-3-yl)methyl bromide (**6a**).

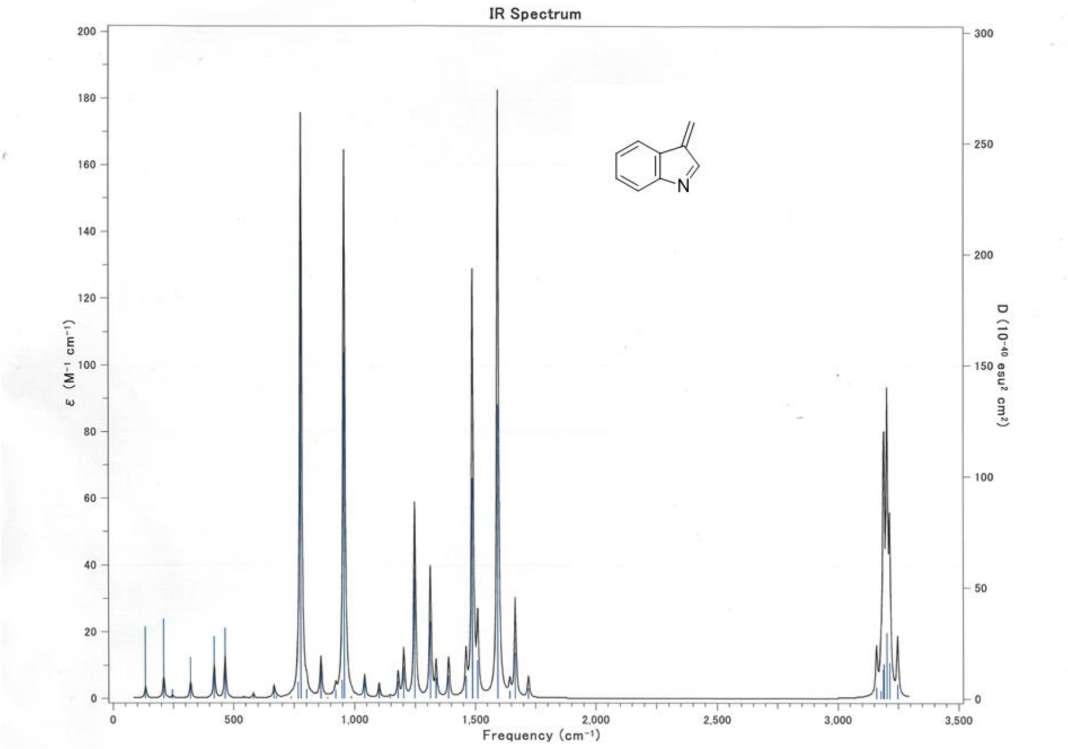

Supplementary Figure 10. Predicted IR spectra of 3-methylene-3*H*-indole.

8. Typical procedure for a micro-flow nucleophilic substitution: method A

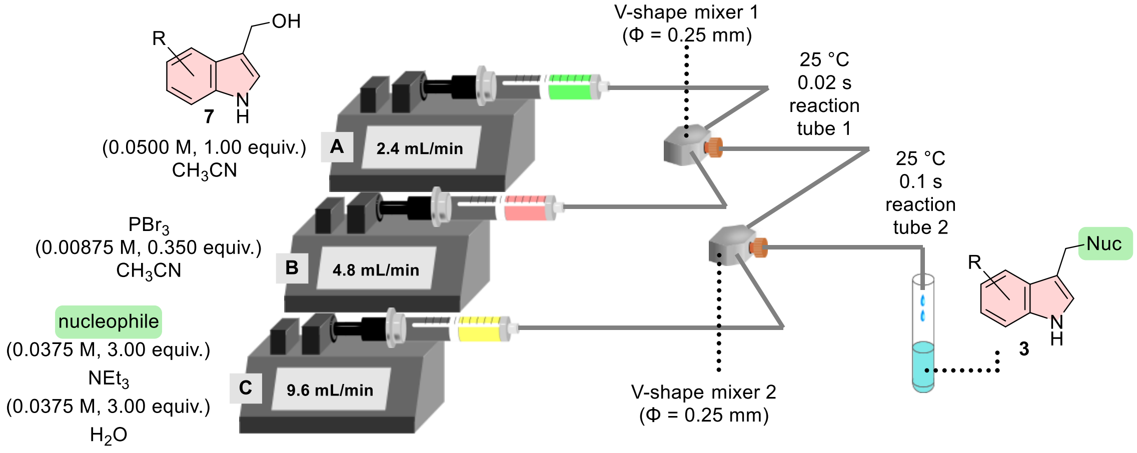

A solution of **alcohol 7** (0.0500 M, 1.00 equiv.) in CH_3CN (flow rate: 2.40 mL/min) and a solution of PBr_3 (0.00875 M, 0.350 equiv.) in CH_3CN (flow rate: 4.80 mL/min) were introduced to V-shape mixer 1 at 25 °C with the syringe pumps. The resultant mixture was passed

through reaction tube 1 (inner diameter: 0.250 mm, length: 48.9 mm, volume: 2.40 μL, reaction time: 0.02 s) at 25 °C. The resultant mixture and a solution of nucleophile (0.0375 M, 3.00 equiv.) and NEt_3 (0.0375 M, 3.00 equiv.) in water (flow rate: 9.60 mL/min) were introduced to V-shape mixer 2 at 25 °C with the syringe pumps. The resultant mixture was passed through the reaction tube 2 (inner diameter: 0.500 mm, length: 143 mm, volume: 28.0 μL, reaction time: 0.1 s) at 25 °C. After being eluted for *ca*. 10 s to reach a steady state, the resultant mixture was poured into a test tube containing sat. $NaHCO_3$ aq. (5 mL) for 45 s at room temperature. The reaction mixture was extracted with ethyl acetate (5 mL) three times. The organic layer was washed with brine (20 mL), dried over Na_2SO_4, filtered, and evaporated *in vacuo*. The residue was purified with recrystallization or PTLC to give **3**.

9. Typical procedure for a micro-flow nucleophilic substitution: method B

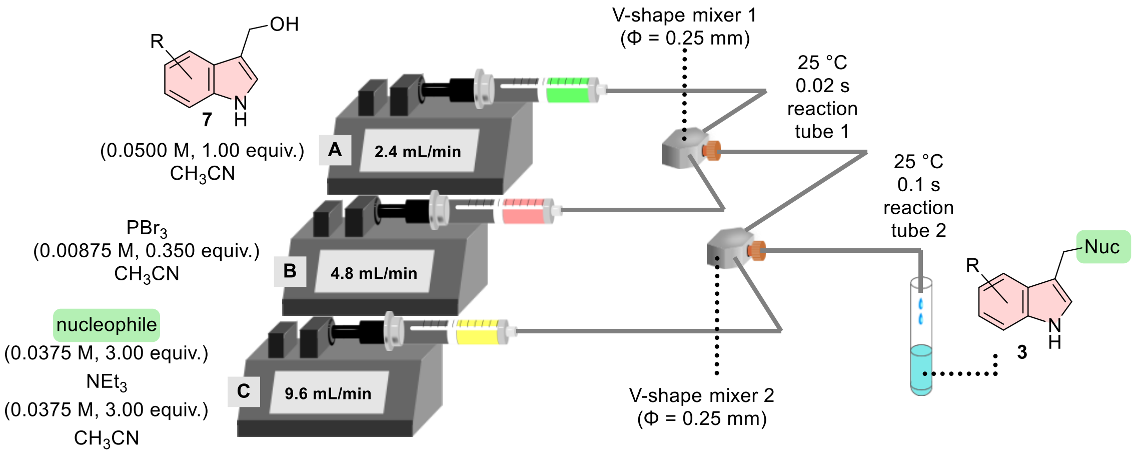

A solution of **alcohol 7** (0.0500 M, 1.00 equiv.) in CH_3CN (flow rate: 2.40 mL/min) and a solution of PBr_3 (0.00875 M, 0.350 equiv.) in CH_3CN (flow rate: 4.80 mL/min) were introduced to V-shape mixer 1 at 25 °C with the syringe pumps. The resultant mixture was passed through reaction tube 1 (inner diameter: 0.250 mm, length: 48.9 mm, volume: 2.40 μL, reaction time: 0.02 s) at 25 °C. The resultant mixture and a solution of nucleophile (0.0375 M, 3.00 equiv.) and NEt_3 (0.0375 M, 3.00 equiv.) in CH_3CN (flow rate: 9.60 mL/min) were introduced to V-shape mixer 2 at 25 °C with the syringe pumps. The resultant mixture was passed through the reaction tube 2 (inner diameter: 0.500 mm, length: 143 mm, volume: 28.0 μL, reaction time: 0.1 s) at 25 °C. After being eluted for *ca*. 10 s to reach a steady state, the resultant mixture was poured into a test tube containing sat. $NaHCO_3$ aq. (5 mL) for 45 s at room temperature. The reaction mixture was extracted with ethyl acetate (5 mL) three times. The organic layer was washed with brine (20 mL), dried over Na_2SO_4, filtered, and

evaporated *in vacuo*. The residue was purified with recrystallization or PTLC to give **3**.

3-((4-(2-Fluorophenyl)piperazin-1-yl)methyl)-1*H*-indole (3a)

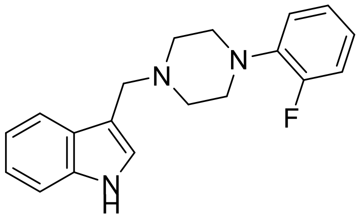

Reaction conditions: **method A**

Purification method: PTLC (hexane : ethyl acetate = 1 : 1, 1% NEt_3)

23.9 mg, 0.077 mmol, 86%

Yellow solid; mp 161–162 °C (lit. 164.6 °C), IR (neat): 3405, 2821, 1499, 1255, 1240, 1007, 747 cm^{-1}; ^1H NMR (400 MHz, $CDCl_3$): δ 8.10 (brs, 1H), 7.78 (d, *J* = 7.8 Hz, 1H), 7.37 (d, *J* = 8.2 Hz, 1H), 7.23-7.12 (m, 3H), 7.06-6.88 (m, 4H), 3.81 (s, 2H), 3.13-3.08 (m, 4H), 2.73-2.70 (m, 4H); ^{13}C NMR (100 MHz, $CDCl_3$): 155.9 (d, *J* = 245.0 Hz), 140.4 (d, *J* = 8.5 Hz), 136.4, 128.2, 124.6 (d, *J* = 2.9 Hz), 123.9, 122.4 (d, *J* = 7.6 Hz), 122.2, 119.7, 119.1, 116.2 (d, *J* = 21.0 Hz), 112.5, 111.2, 53.4 (d, *J* = 41.9 Hz), 50.8, 50.7; ^{19}F NMR (376 MHz, $CDCl_3$): δ -122.6; ^1H NMR (400 MHz, DMSO-d_6): δ 10.93 (brs, 1H), 7.65 (d, *J* = 7.8 Hz, 1H), 7.35 (d, *J* = 8.2 Hz, 1H), 7.25 (d, *J* = 2.3 Hz, 1H), 7.12-6.91 (m, 6H), 3.68 (s, 2H), 3.00-2.97 (m, 4H), 2.56 (m, 4H); ^{13}C NMR (100 MHz, DMSO-d_6): δ 154.9 (d, *J* = 243.1 Hz), 140.0 (d, *J* = 7.6 Hz), 136.3, 127.6, 124.8, 124.7, 122.1 (d, *J* = 7.7 Hz), 120.9, 119.2, 119.1, 118.4, 115.9 (d, *J* = 21.0 Hz), 111.3, 110.6, 53.1, 52.5, 50.2; HRMS (ESI): calcd for $[C_{19}H_{20}FN_3+Na]^+$ 332.1533, found 332.1540.

Spectral data of ^1H NMR (DMSO-d_6) was well consistent with that in the previous report.[7]

3-(Azidomethyl)-1*H*-indole (3b)

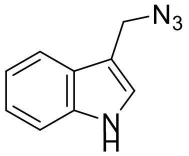

Reaction conditions: **method A**

Pure 3-(azidomethyl)-1*H*-indole (**3b**) was obtained only by simple aqueous work-up. The mixture of dichloromethane and methanol (10 : 1) was used for extraction instead of ethyl acetate.

14.7 mg, 0.0855 mmol, 95%

Brown oil; IR (neat): 3419, 2096, 1558, 1541, 1507, 1457, 744 cm^{-1}; ^1H NMR (400 MHz,

$CDCl_3$): δ 8.18 (brs, 1H), 7.69 (d, J = 8.2 Hz, 1H), 7.41 (d, J = 8.2 Hz, 1H), 7.30-7.17 (m, 3H), 4.54 (s, 2H); ^{13}C NMR (100 MHz, $CDCl_3$): δ 136.8, 126.7, 124.0, 122.9, 120.4, 119.0, 111.5, 110.5, 46.7; HRMS (ESI): calcd for $[C_9H_8N_4+Na]^+$ 195.0641, found 195.0642.

_N_-((1_H_-Indol-3-yl)methyl)-_N_-isopropylpropan-2-amine (3c)

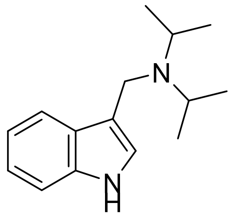

Reaction conditions: **method B**

Purification method: PTLC (ethyl acetate, 1% NEt_3)

14.2 mg, 0.0616 mmol, 68%

Pink oil; IR (neat): 3414, 2963, 2926, 1456, 1419, 1387, 1337, 1201, 1178, 1010, 740 cm^{-1}; ^1H NMR (400 MHz, $CDCl_3$): δ 8.09 (brs, 1H), 7.81 (d, J = 8.2 Hz, 1H), 7.34 (d, J = 8.2 Hz, 1H), 7.19-7.06 (m, 3H), 3.84 (d, J = 0.9 Hz, 2H), 3.12 (m, 2H), 1.06 (d, J = 6.4 Hz, 12H); ^{13}C NMR (100 MHz, $CDCl_3$): δ 136.8, 127.5, 122.9, 121.9, 120.1, 119.1, 117.2, 111.0, 47.3, 40.6, 20.7; HRMS (ESI): calcd for $[C_{15}H_{22}N_2+Na]^+$ 253.1675, found 253.1676.

3-(Pyrrolidin-1-ylmethyl)-1_H_-indole (3d)

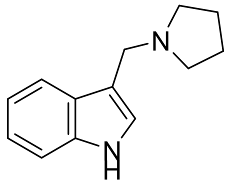

Reaction conditions: **method A**

Purification method: PTLC (methanol, 1% NEt_3)

11.9 mg, 0.0594 mmol, 66%

Yellow solid; mp 122–123 °C (lit. 123 °C), ^1H NMR (400 MHz, $CDCl_3$): δ 8.08 (brs, 1H), 7.72 (d, J = 8.2 Hz, 1H), 7.36 (d, J = 8.2 Hz, 1H), 7.22-7.11 (m, 3H), 3.84 (s, 2H), 2.61-2.56 (m, 4H), 1.82-1.73 (m, 4H); ^{13}C NMR (100 MHz, $CDCl_3$): δ 136.2, 127.9, 123.5, 122.0, 119.5, 119.3, 113.9, 111.2, 54.3, 50.5, 23.7; HRMS (ESI): calcd for $[C_{13}H_{16}N_2+Na]^+$ 223.1206, found 223.1206.

Spectral data of ^1H NMR ($CDCl_3$) was well consistent with that in the previous report.[8]

4-((1*H*-Indol-3-yl)methyl)morpholine (3e)

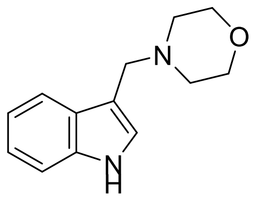

Reaction conditions: **method A**

Purification method: PTLC (ethyl acetate, 1% NEt_3)

16.1 mg, 0.0744 mmol, 83%

Yellow solid; mp 121–123 °C (lit. 119–120 °C), ^{1}H NMR (400 MHz, $CDCl_3$): δ 8.07 (brs, 1H), 7.76 (d, *J* = 7.8 Hz, 1H), 7.37 (d, *J* = 7.8 Hz, 1H), 7.21 (dd, *J* = 1.4, 7.8 Hz, 1H), 7.16-7.12 (m, 2H), 3.72-3.69 (m, 6H), 2.51 (t, *J* = 4.6 Hz, 4H); ^{13}C NMR (100 MHz, $CDCl_3$): δ 136.4, 128.0, 123.8, 122.2, 119.7, 119.6 112.4, 111.2, 67.3, 54.2, 53.7; HRMS (ESI): calcd for $[C_{13}H_{16}N_2+Na]^+$ 239.1155, found 239.1155.

Spectral data of ^{1}H NMR ($CDCl_3$) was well consistent with that in the previous report.[9]

4-((5-Methyl-1*H*-indol-3-yl)methyl)morpholine (3f)

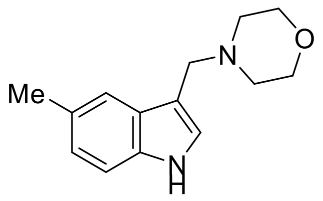

Reaction conditions: **method A**

Purification method: PTLC (ethyl acetate, 1% NEt_3)

16.7 mg, 0.0725 mmol, 81%

yellow oil; IR (neat): 2915, 2857, 2808, 1456, 1114, 1000, 862, 790 cm^{-1}; ^{1}H NMR (400 MHz, $CDCl_3$): δ 7.98 (brs, 1H), 7.52 (s, 1H), 7.29-7.25 (m, 1H), 7.11 (d, *J* = 1.4 Hz, 1H), 7.03 (dd, *J* = 1.4, 8.2 Hz, 1H), 3.71 (t, *J* = 4.6 Hz, 4H), 3.68 (s, 2H), 2.51, (t, *J* = 4.6 Hz, 4H), 2.47 (s, 3H); ^{13}C NMR (100 MHz, $CDCl_3$): δ 134.7, 128.9, 128.3, 124.0, 123.8, 119.2, 111.7, 110.9, 67.2, 54.1, 53.7, 23.7; HRMS (ESI): calcd for $[C_{14}H_{18}N_2O+Na]^+$ 253.1311, found 253.1311.

4-((5-Bromo-1*H*-indol-3-yl)methyl)morpholine (3g)

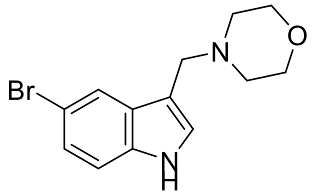

Reaction conditions: **method A**

Purification method: Recrystallization from ethylacetate/hexane

22.0 mg, 0.0745 mmol, 83%

Brown solid; mp 150–152 °C, IR (neat): 3734, 3649, 1558, 1541, 1507, 1457, 1456, 861, 791 cm^{-1}; ^{1}H NMR (400 MHz, $CDCl_3$): δ 8.09 (brs, 1H), 7.90 (d, *J* = 1.4 Hz, 1H), 7.30-7.22 (m, 2H), 7.14 (d, *J* = 1.4 Hz, 1H), 3.71 (t, *J* = 4.6 Hz, 4H), 3.65 (s, 2H), 2.48 (m, 4H); ^{13}C NMR (100 MHz, $CDCl_3$): δ 135.0, 129.7, 125.1, 124.9, 122.4, 113.0, 112.6, 112.2, 67.2, 54.0, 53.4; HRMS (ESI): calcd for $[C_{13}H_{15}BrN_2O+Na]^+$ 317.0260, found 317.0252.

4-((2-Phenyl-1*H*-indol-3-yl)methyl)morpholine (3h)

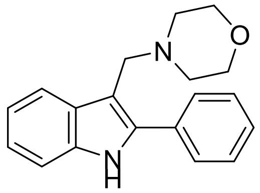

The substrate **6j** was synthesized according to previous report.[10]

Reaction conditions: **method A**

Purification method: PTLC (hexane : ethyl acetate = 3 : 1, 1% NEt_3)

18.7 mg, 0.0640 mmol, 71%

White solid; mp 149–152 °C, IR (neat): 1558, 1531, 1507, 1457, 1111, 741, 700 cm^{-1}; ^{1}H NMR (400 MHz, $CDCl_3$): δ 8.20 (brs, 1H), 7.82-7.79 (m, 3H), 7.49 (t *J* = 7.8 Hz, 2H), 7.40 (d *J* = 7.8 Hz, 2H), 7.22 (dd, *J* = 1.4, 7.8 Hz, 1H), 7.16 (dd, *J* = 1.4, 7.8 Hz, 1H), 3.71-3.69 (m, 6H), 2.55 (m, 4H); ^{13}C NMR (100 MHz, $CDCl_3$): δ 137.0, 125.6, 133.0, 130.2, 128.9, 128.5, 127.9, 122.4, 120.0, 119.8, 110.9, 109.6, 67.4, 53.6, 52.9; HRMS (ESI): calcd for $[C_{19}H_{20}N_2O+Na]^+$ 315.1468, found 315.1464.

tert-Butyl ((1H-indol-3-yl)methyl)-L-prolinate (3i)

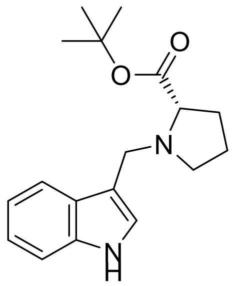

Reaction conditions: **method A**

Purification method: PTLC (hexane : ethyl acetate = 1 : 1, 1% NEt_3)

22.8 mg, 0.0759 mmol, 84%

Yellow solid; mp 107–109 °C, IR (neat): 1732, 1718, 1457, 1366, 1155, 741 cm^{-1}; $[\square]^{23}_{D}$ = -110.1 (c 0.50, CH_2Cl_2); ^1H NMR (400 MHz, $CDCl_3$): δ 8.06 (brs, 1H), 7.78 (d, *J* = 7.8 Hz, 1H), 7.35 (d, *J* = 7.8 Hz, 1H), 7.21-7.10 (m, 3H), 4.13 (d, *J* = 13.3 Hz, 1H), 3.84 (d, *J* = 13.3 Hz, 1H), 3.17-3.12 (m, 1H), 3.10-3.04 (m, 1H), 2.44 (dd, *J* = 8.7, 16.5 Hz, 1H), 2.09-2.00 (m, 1H), 1.96-1.79 (m, 2H), 1.75-1.65 (m, 1H), 1.46 (s, 9H); ^{13}C NMR (100 MHz, $CDCl_3$): δ 173.9, 136.3, 128.2, 124.0, 121.9, 119.6, 119.5, 112.7, 111.1, 80.6, 65.4, 53.3, 48.2, 29.5, 28.2, 23.0; HRMS (ESI): calcd for $[C_{18}H_{24}N_2O_2+Na]^+$ 323.1730, found 323.1730.

Ethyl N-((1H-indol-3-yl)methyl)-N-methylglycinate (3j)

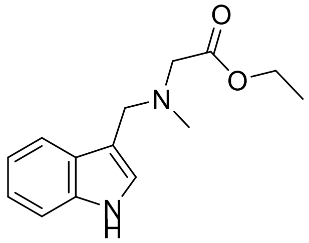

Reaction conditions: **method A**

Purification method: PTLC (hexane : ethyl acetate = 1 : 1, 1% NEt_3)

18.5 mg, 0.0750 mmol, 83%

White solid; mp 81–83 °C, IR (neat): 1734, 1717, 1558, 1541, 1507, 1457, 1191, 741 cm^{-1}; ^1H NMR (400 MHz, $CDCl_3$): δ 8.10 (brs, 1H), 7.76 (d, *J* = 7.8 Hz, 1H), 7.36 (d, *J* = 7.8 Hz, 1H), 7.22-7.11 (m, 3H), 4.17 (q, *J* = 7.3 Hz, 2H), 3.89 (s, 2H), 3.27 (s, 2H), 2.43 (s, 3H), 1.26 (t, *J* = 7.3 Hz, 3H); ^{13}C NMR (100 MHz, $CDCl_3$): δ 171.5, 136.4, 128.0, 124.1, 122.2, 119.8, 119.6, 112.7, 111.2, 60.5, 57.6, 51.9, 42.7, 14.4; HRMS (ESI): calcd for $[C_{14}H_{18}N_2O_2+Na]^+$ 269.1260, found 269.1260.

3-(Bis((1*H*-indol-3-yl)methyl)amino)propan-1-ol (3k)

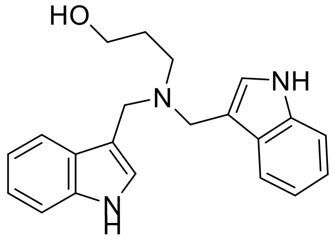

Reaction conditions: **method A**

Purification method: PTLC (ethyl acetate 1% NEt_3)

7.30 mg, 0.0219 mmol, 49%

Yellow oil; IR (neat): 1558, 1541, 1507, 1457, 1339, 741 cm^{-1}; ^1H NMR (400 MHz, $CDCl_3$): δ 8.24 (brs, 2H), 7.62 (d, *J* = 7.8 Hz, 2H), 7.37 (d, *J* = 7.8 Hz, 2H), 7.22-7.17 (m, 4H), 7.11 (dd, *J* = 0.9, 7.8 Hz, 2H), 3.89 (s, 4H), 3.66 (t, *J* = 5.5 Hz, 2H), 2.79 (t, *J* = 5.5 Hz, 2H), 1.82 (m, 2H); ^{13}C NMR (100 MHz, $CDCl_3$): δ 136.3, 128.0, 124.0, 122.2, 119.8, 119.2, 112.7, 111.3, 64.4, 53.7, 49.2, 28.0; HRMS (ESI): calcd for $[C_{21}H_{23}N_3O+Na]^+$ 356.1733, found 356.1733.

***N*,*N*-Bis((1*H*-indol-3-yl)methyl)-1-phenylmethanamine (3l)**

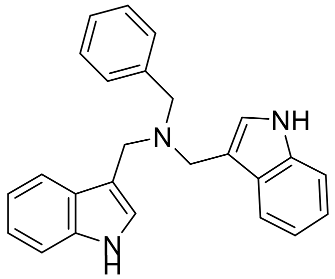

Reaction conditions: **method B**

Purification method: PTLC (hexane : ethyl acetate = 1 : 1, 1% NEt_3)

11.2 mg, 0.0306 mmol, 68%

Yellow oil; IR (neat): 3413, 1558, 1541, 1456, 1419, 1338, 741 cm^{-1}; ^1H NMR (400 MHz, $CDCl_3$): δ 8.00 (brs, 2H), 7.68 (d, *J* = 7.8 Hz, 2H), 7.40 (d, *J* = 7.8 Hz, 2H), 7.35-7.28 (m, 4H), 7.23-7.15 (m, 5H), 7.08 (dd, *J* = 0.9, 7.8 Hz, 2H), 3.81 (s, 4H), 3.64 (s, 2H); ^{13}C NMR (100 MHz, $CDCl_3$): δ 140.5, 136.6, 129.2, 128.2, 128.0, 126.7, 123.5, 122.0, 120.2, 119.4, 114.3, 111.0, 58.3, 49.4; HRMS (ESI): calcd for $[C_{25}H_{23}N_3+Na]^+$ 388.1784, found 388.1784.

_N_-((1_H_-Indol-3-yl)methyl)-2-bromoaniline (3m)

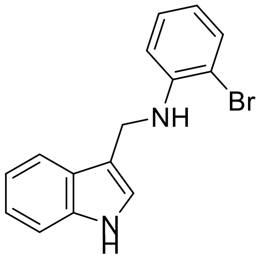

Reaction conditions: **method B**

Purification method: PTLC (hexane : ethyl acetate = 4 : 1, 1% NEt_3)

13.5 mg, 0.0448 mmol, 50%

Brown oil; IR (neat): 3412, 1597, 1507, 1456, 1497, 1018, 741 cm^{-1}; ^{1}H NMR (400 MHz, $CDCl_3$): δ 8.08 (brs, 1H), 7.69 (d, J = 8.2 Hz, 1H), 7.45-7.40 (m, 2H), 7.30-7.13 (m, 4H), 6.80 (dd, J = 1.4, 8.2 Hz, 1H), 6.59 (dd, J = 1.4, 8.2 Hz, 1H), 4.62 (brs, 1H), 4.54 (d, J = 4.6 Hz, 2H); ^{13}C NMR (100 MHz, $CDCl_3$): δ 145.2, 126.6, 132.5, 128.6, 126.7, 122.9, 122.6, 120.0, 119.1, 117.9, 113.4, 111.7, 111.5, 109.8, 40.2; HRMS (ESI): calcd for $[C_{15}H_{13}BrN_2+Na]^+$ 323.0154, found 323.0154.

3-((Phenylsulfonyl)methyl)-1_H_-indole (3n)

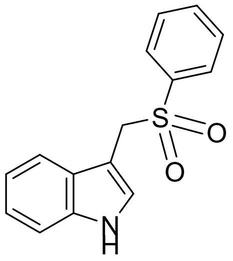

Reaction conditions: **method A**

Purification method: PTLC (hexane : ethyl acetate = 3 : 1, 1% NEt_3)

18.8 mg, 0.0693 mmol, 77%

Yellow solid; mp 159–161 °C (lit. 163–164 °C), ^{1}H NMR (400 MHz, $CDCl_3$): δ 8.24 (brs, 1H), 7.68 (dd, J = 1.8, 7.8 Hz, 2H), 7.56-7.52 (m, 1H), 7.40-7.22 (m, 4H), 7.18-7.14 (m, 1H), 7.09 (d, J = 1.8 Hz, 1H), 7.04-7.00 (m, 1H), 4.54 (s, 2H); ^{13}C NMR (100 MHz, $CDCl_3$): δ 138.4, 135.8, 133.6, 129.0, 128.7, 127.0, 126.0, 122.6, 120.5, 118.6, 111.4, 103.0, 54.6; HRMS (ESI): calcd for $[C_{15}H_{13}NO_2S+Na]^+$ 294.0559, found 294.0559.

Spectral data of ^{1}H NMR ($CDCl_3$) was well consistent with that in the previous report.[11]

3-((Decylthio)methyl)-1*H*-indole (3o)

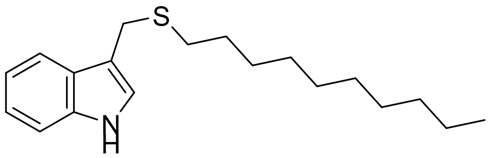

Reaction conditions: **method B**

Purification method: PTLC (hexane : ethyl acetate = 9 : 1)

18.5 mg, 0.0695 mmol, 77%

White solid; mp 59–61 °C, IR (neat): 3391, 2918, 2849, 1457, 741 cm^{-1}; ^1H NMR (400 MHz, $CDCl_3$): δ 7.99 (brs, 1H), 7.73 (d, *J* = 7.8 Hz, 1H), 7.36 (d, *J* = 7.8 Hz, 1H), 7.23-7.12 (m, 3H), 3.94 (d, *J* = 0.9 Hz, 2H), 2.47 (t, *J* = 7.3 Hz, 2H), 1.62-1.55 (m, 2H), 1.40-1.21 (m, 14H), 0.88 (t, *J* = 7.32 Hz, 3H); ^{13}C NMR (100 MHz, $CDCl_3$): δ 136.6, 127.0, 122.9, 122.4, 119.7, 119.4, 113.0, 111.3, 32.0, 31.9, 29.7, 29.7, 29.5, 29.5, 29.4, 29.1, 27.0, 22.8, 14.3; HRMS (ESI): calcd for $[C_{19}H_{29}NS+Na]^+$ 326.1913, found 326.1912.

3-((Phenylthio)methyl)-1*H*-indole (3p)

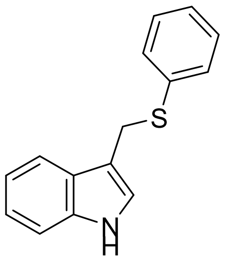

Reaction conditions: **method B**

Purification method: PTLC (hexane : ethyl acetate = 5 : 1, 1% NEt_3)

20.5 mg, 0.0857 mmol, 95%

White solid; mp 82–83 °C (lit. 80.5–81.5 °C), ^1H NMR (400 MHz, $CDCl_3$): δ 7.98 (brs, 1H), 7.71 (d, *J* = 8.2 Hz, 1H), 7.37-7.33 (m, 3H), 7.30-7.13 (m, 5H), 7.08 (d, *J* = 2.3 Hz, 1H), 4.36 (s, 2H); ^{13}C NMR (100 MHz, $CDCl_3$): δ 137.3, 136.4, 129.7, 128.9, 126.9, 126.1, 123.3, 122.5, 120.0, 119.2, 111.9, 111.4, 29.9; HRMS (ESI): calcd for $[C_{15}H_{13}NS+Na]^+$ 262.0661, found 262.0661.

Spectral data of ^1H NMR ($CDCl_3$) was well consistent with that in the previous report.[12]

5-Bromo-3-((phenylthiol)methyl)-1*H*-indole (3q)

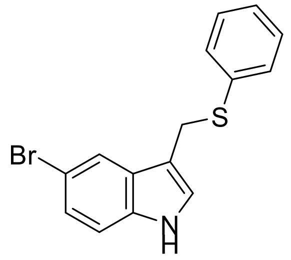

Reaction conditions: **method B**

Purification method: PTLC (hexane : ethyl acetate = 3 : 1, 1% NEt_3)

29.1mg, 0.0914 mmol, quant.

White solid; mp 102–104 °C, IR (neat): 3734, 3649, 1698, 1558, 1541, 1507, 1457 cm^{-1}; ^{1}H NMR (400 MHz, $CDCl_3$): δ 8.01 (brs, 1H), 7.80 (d, *J* = 1.4 Hz, 1H), 7.35-7.17 (m, 7H), 7.06 (d, *J* = 1.4 Hz, 1H), 4.28 (s, 2H); ^{13}C NMR (100 MHz, $CDCl_3$): δ 136.8, 135.0, 130.1, 129.0, 128.6, 126.4, 125.4, 124.4, 122.0, 113.2, 112.8, 111.8, 29.9; HRMS (ESI): calcd for $[C_{15}H_{12}BrNS+Na]^+$ 339.9766, found 339.9765.

5,5-Bis((1*H*-indol-3-yl)methyl)-2,2-dimethyl-1,3-dioxane-4,6-dione (3r)

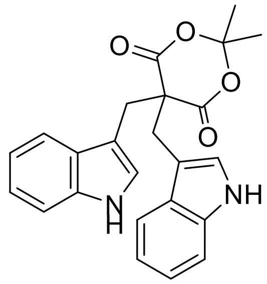

Reaction conditions: **method A**

Purification method: PTLC (hexane : ethyl acetate = 3 : 1, 1% NEt_3)

15.6 mg, 0.0388 mmol, 86%

Yellow solid; mp 197–199 °C, IR (neat): 3411, 1732, 1717, 1457, 1362, 1273, 743 cm^{-1}; ^{1}H NMR (400 MHz, $CDCl_3$): δ 8.08 (brs, 2H), 7.71 (dd, *J* = 1.8, 8.2 Hz, 2H), 7.30 (dd, *J* = 1.8, 8.2 Hz, 2H), 7.18-7.10 (m, 6H), 3.74 (s, 4H), 0.51 (s, 6H); ^{13}C NMR (100 MHz, $CDCl_3$): δ 170.2, 135.9, 126.8, 124.5, 122.5, 120.3, 120.0, 111.0, 110.1, 105.7, 59.6, 35.4, 28.6; HRMS (ESI): calcd for $[C_{24}H_{22}N_2O_4+Na]^+$ 425.1472, found 425.1472.

NMR chart

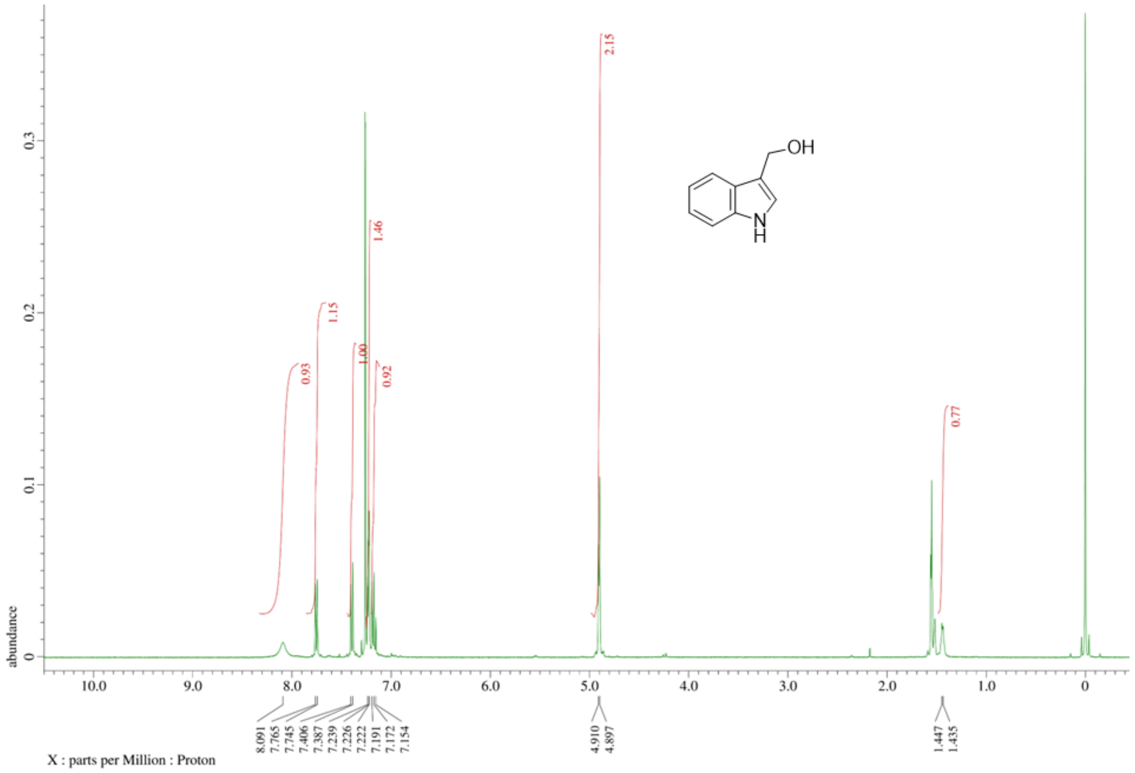

Supplementary Figure 11. Purchased alcohol **7a**, ^{1}H NMR (400 MHz, $CDCl_3$).

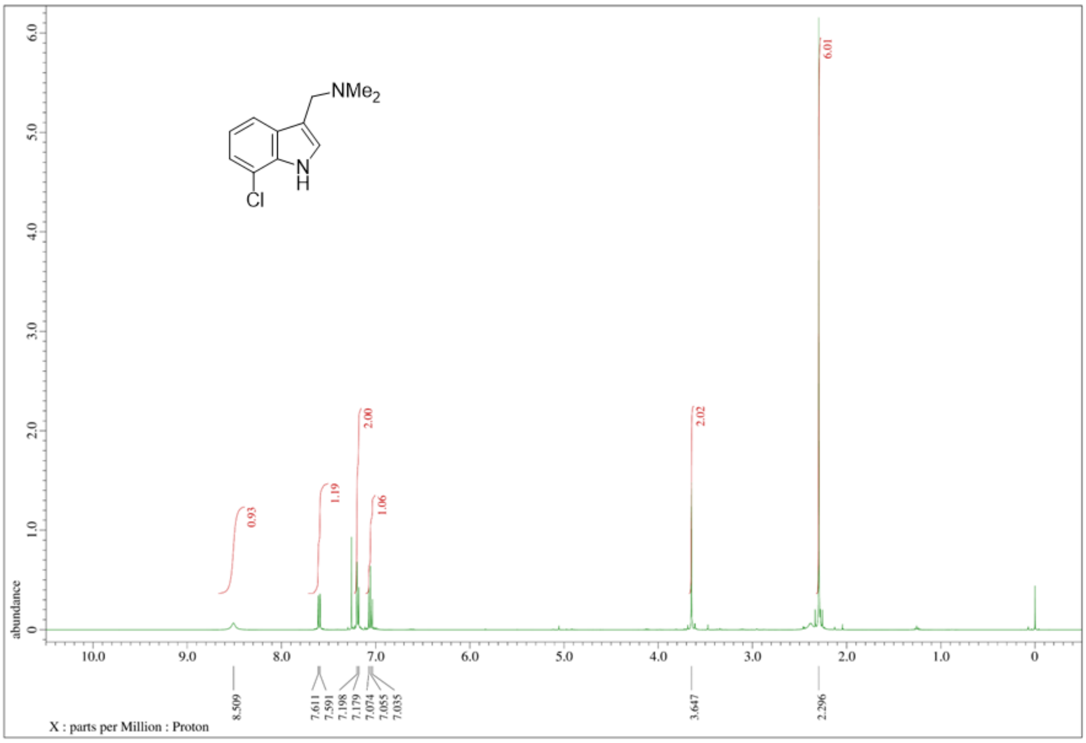

Supplementary Figure 12. Commpound **14**, ^{1}H NMR (400 MHz, $CDCl_3$).

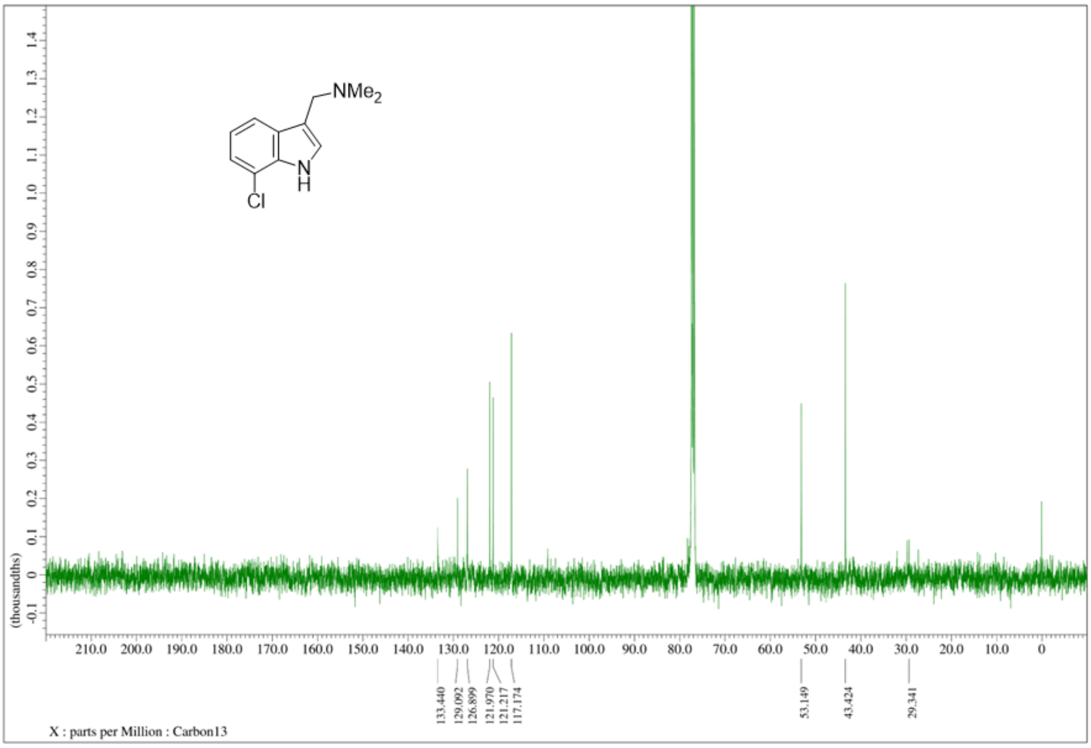

Supplementary Figure 13. Commpound **14**, ^{13}C NMR (100 MHz, $CDCl_3$).

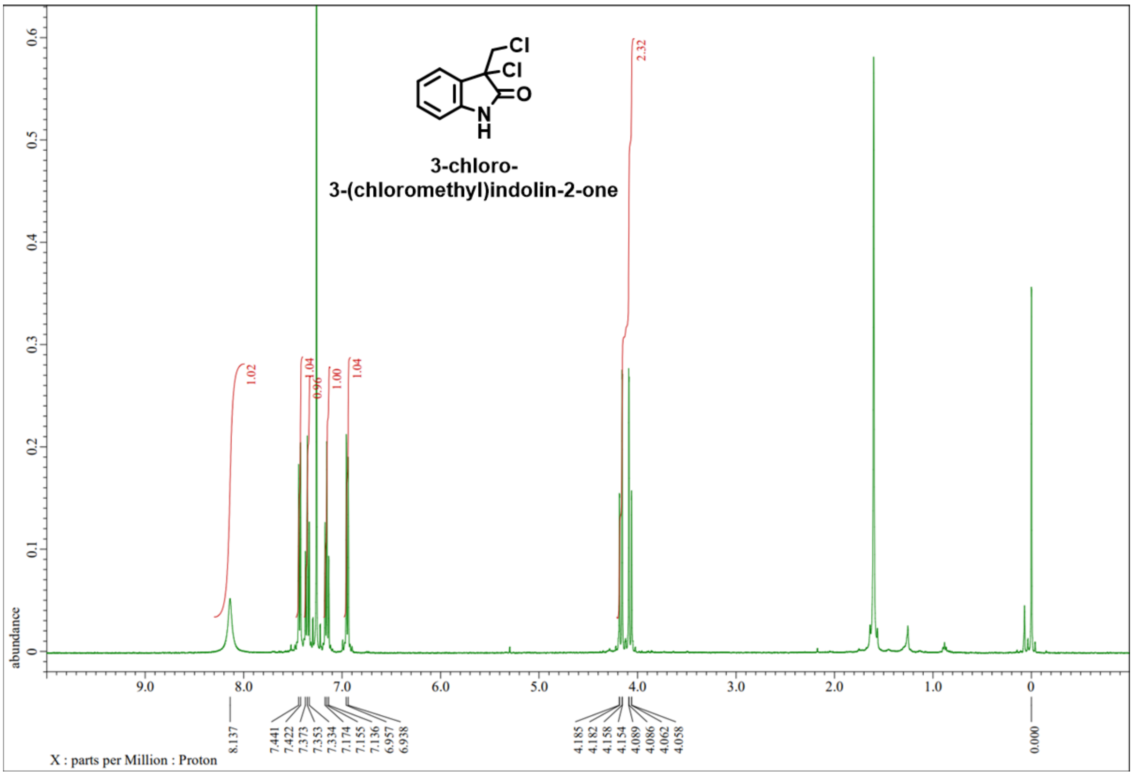

Supplementary Figure 14. Commpound **17**, ^{1}H NMR (400 MHz, $CDCl_3$).

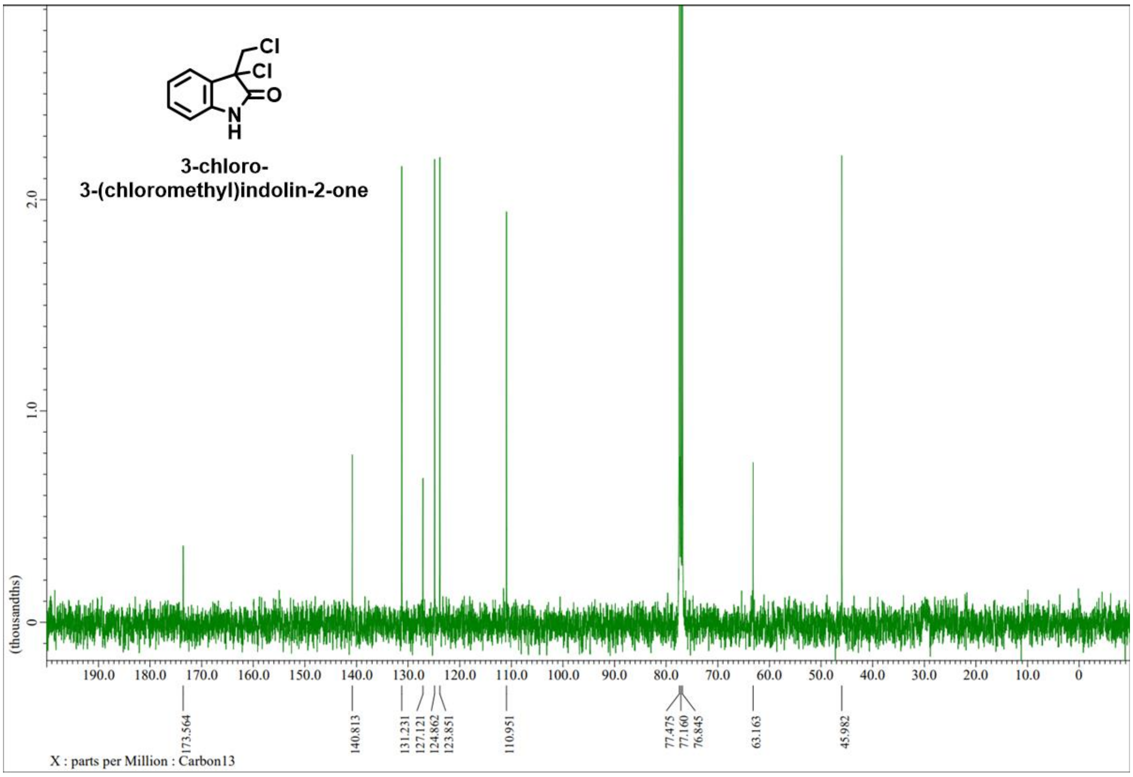

Supplementary Figure 15. Commpound **17**, ^{13}C NMR (100 MHz, $CDCl_3$).

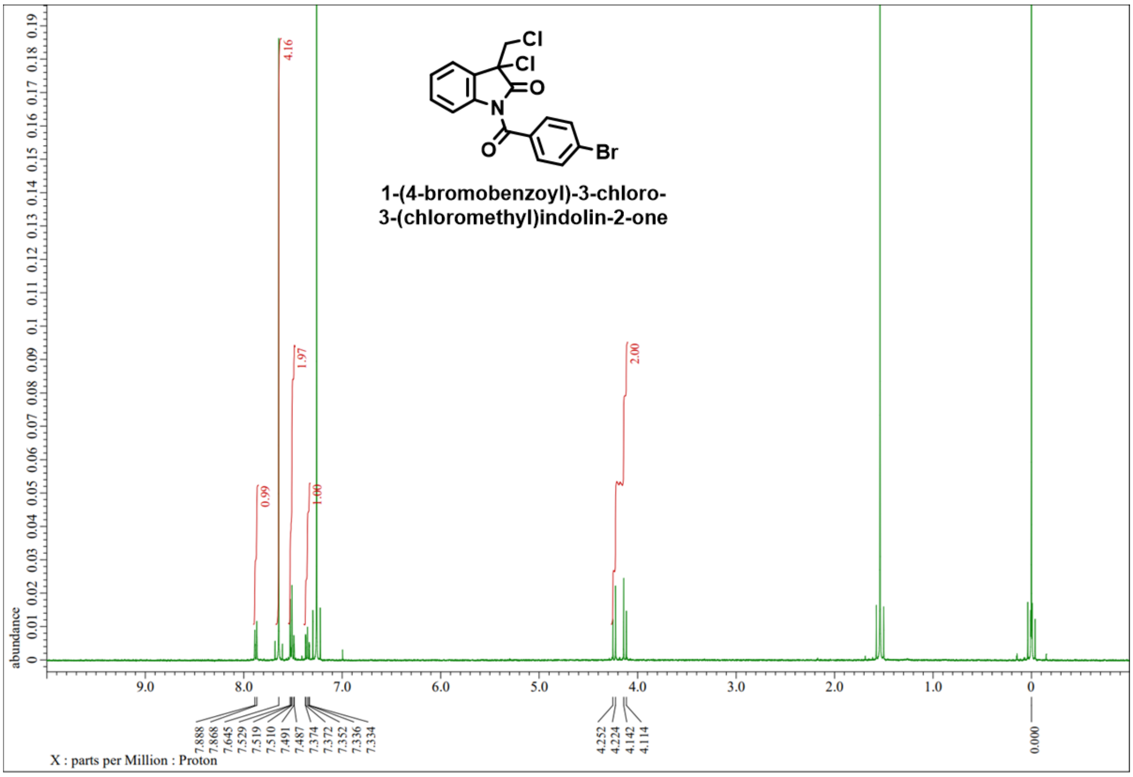

Supplementary Figure 16. Commpound **19**, ^{1}H NMR (400 MHz, $CDCl_3$).

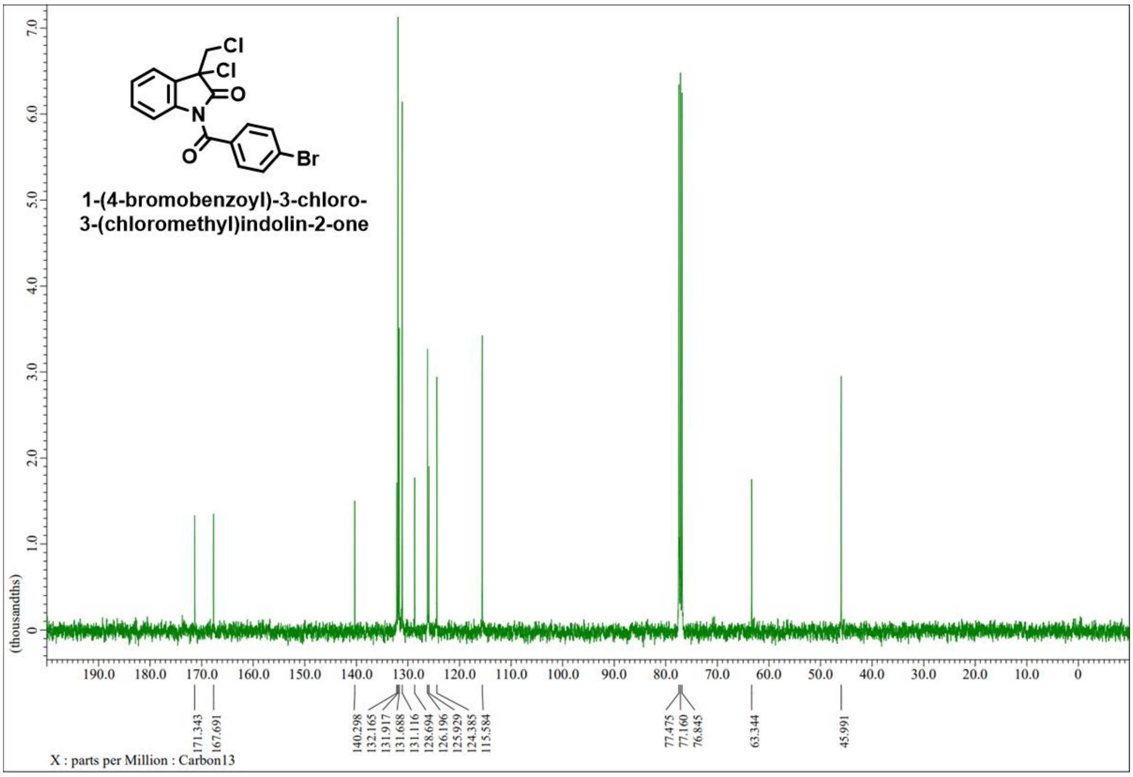

Supplementary Figure 17. Commpound **19**, ^{13}C NMR (100 MHz, $CDCl_3$).

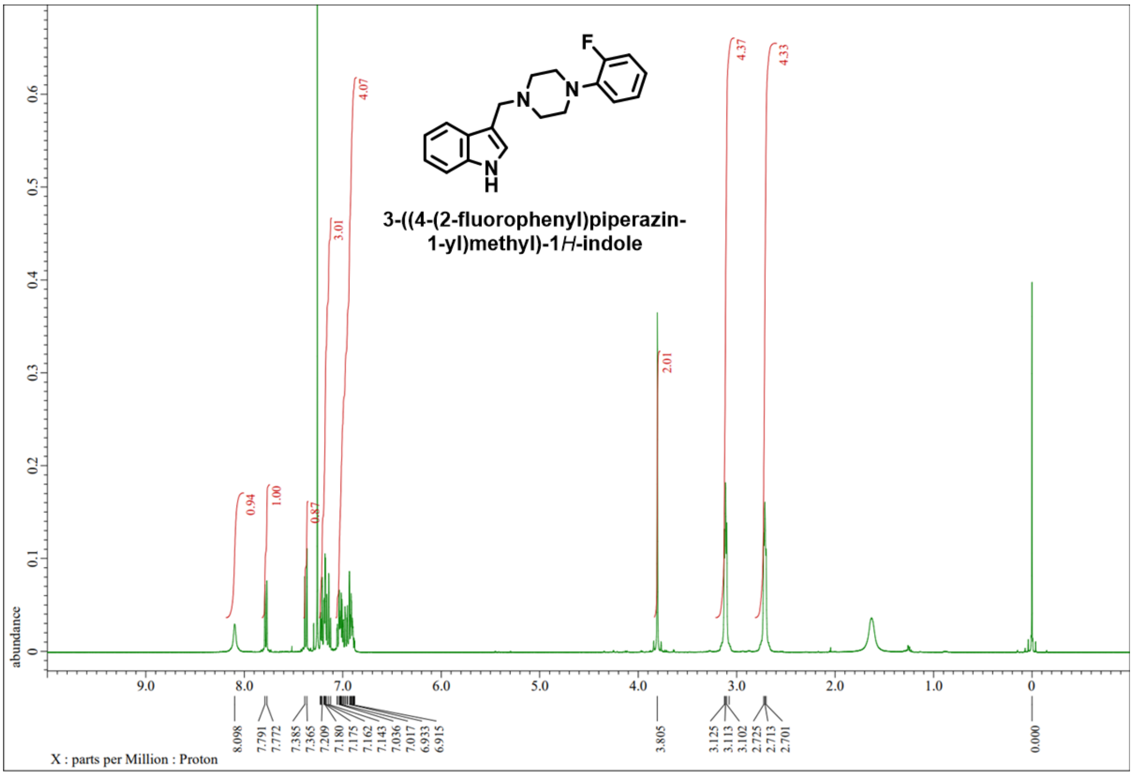

Supplementary Figure 18. Commpound **3a**, ^{1}H NMR (400 MHz, $CDCl_3$).

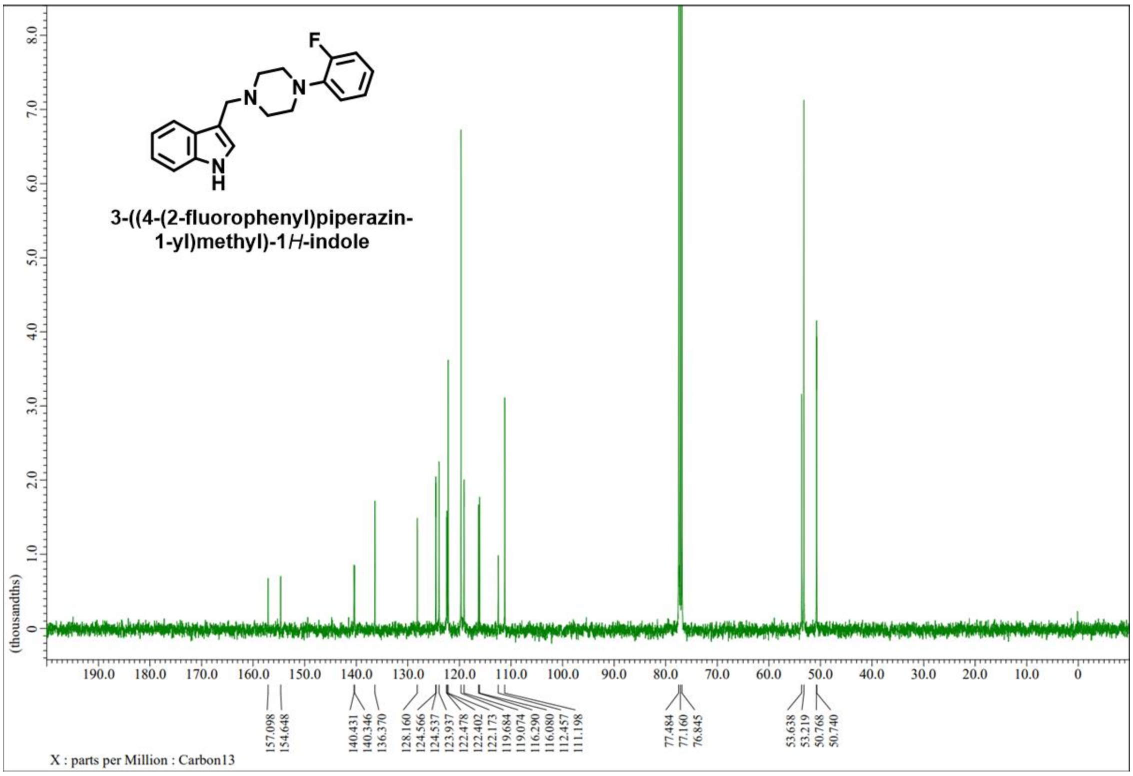

Supplementary Figure 19. Commpound **3a**, ^{13}C NMR (100 MHz, $CDCl_3$).

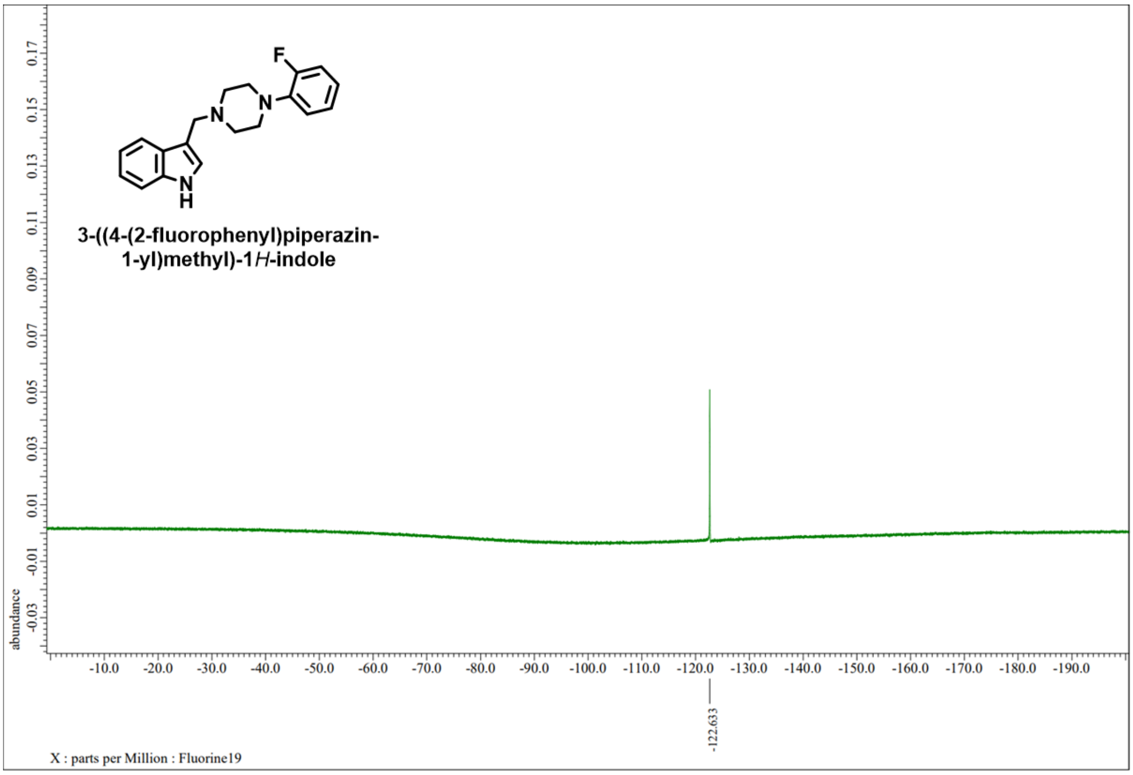

Supplementary Figure 20. Commpound **3a**, ^{19}F NMR (376 MHz, $CDCl_3$).

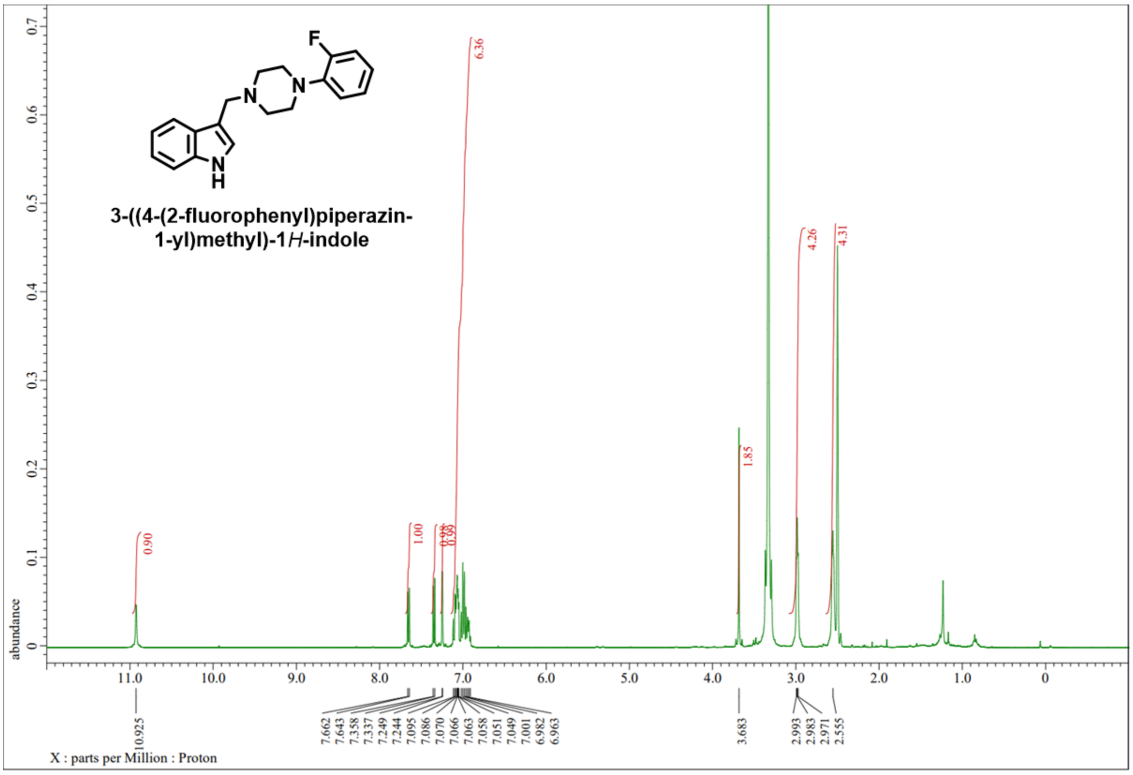

Supplementary Figure 21. Commpound **3a**, ^{1}H NMR (400 MHz, DMSO-d_6).

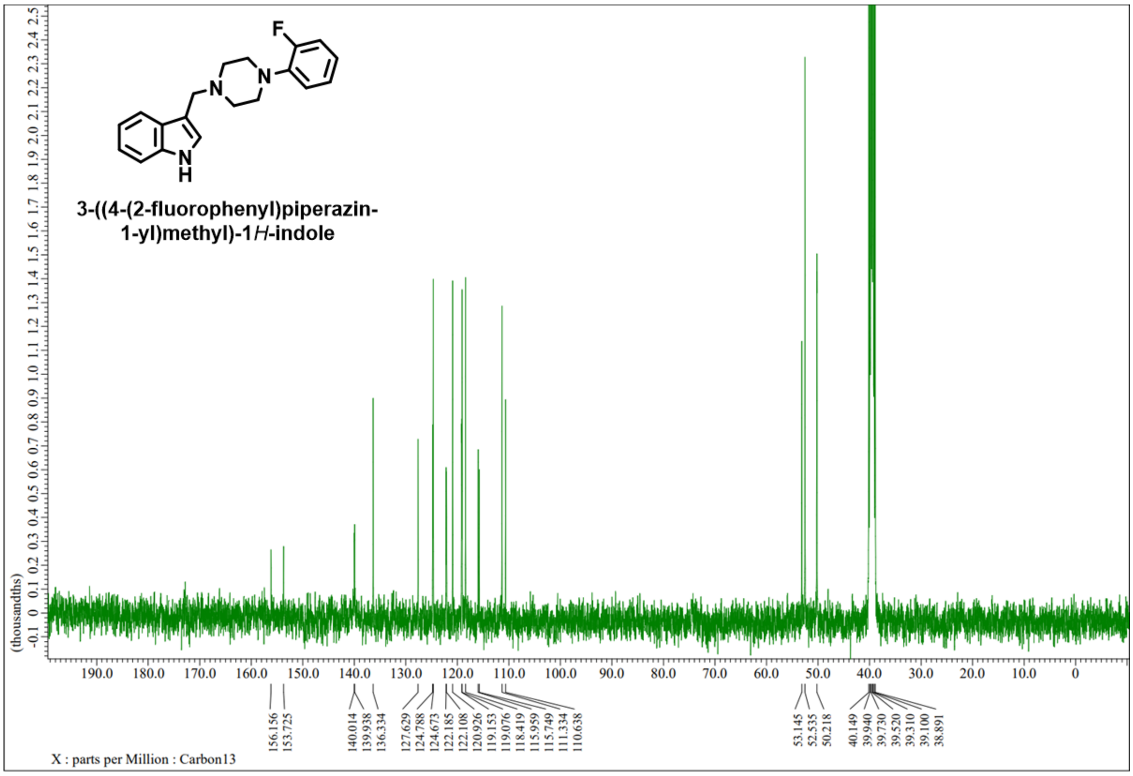

Supplementary Figure 22. Commpound **3a**, ^{13}C NMR (100 MHz, DMSO-d_6).

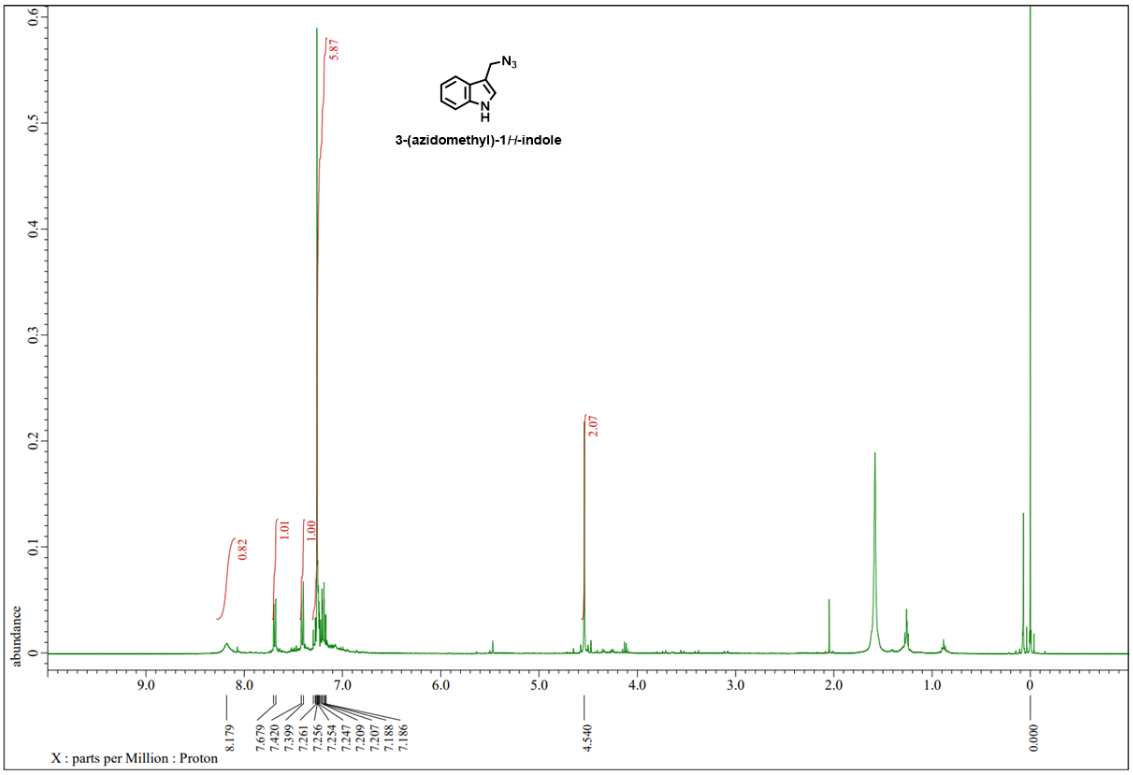

Supplementary Figure 23. Commpound **3b**, ^{1}H NMR (400 MHz, $CDCl_3$).

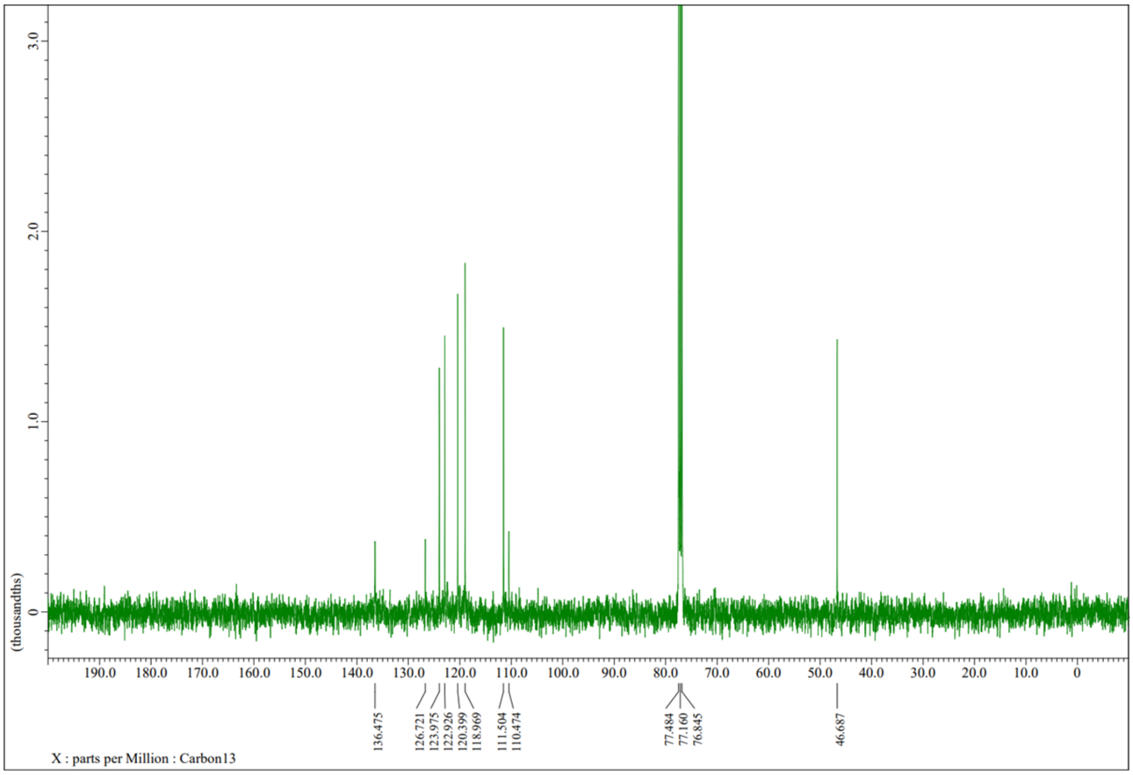

Supplementary Figure 24. Commpound **3b**, ^{13}C NMR (100 MHz, $CDCl_3$).

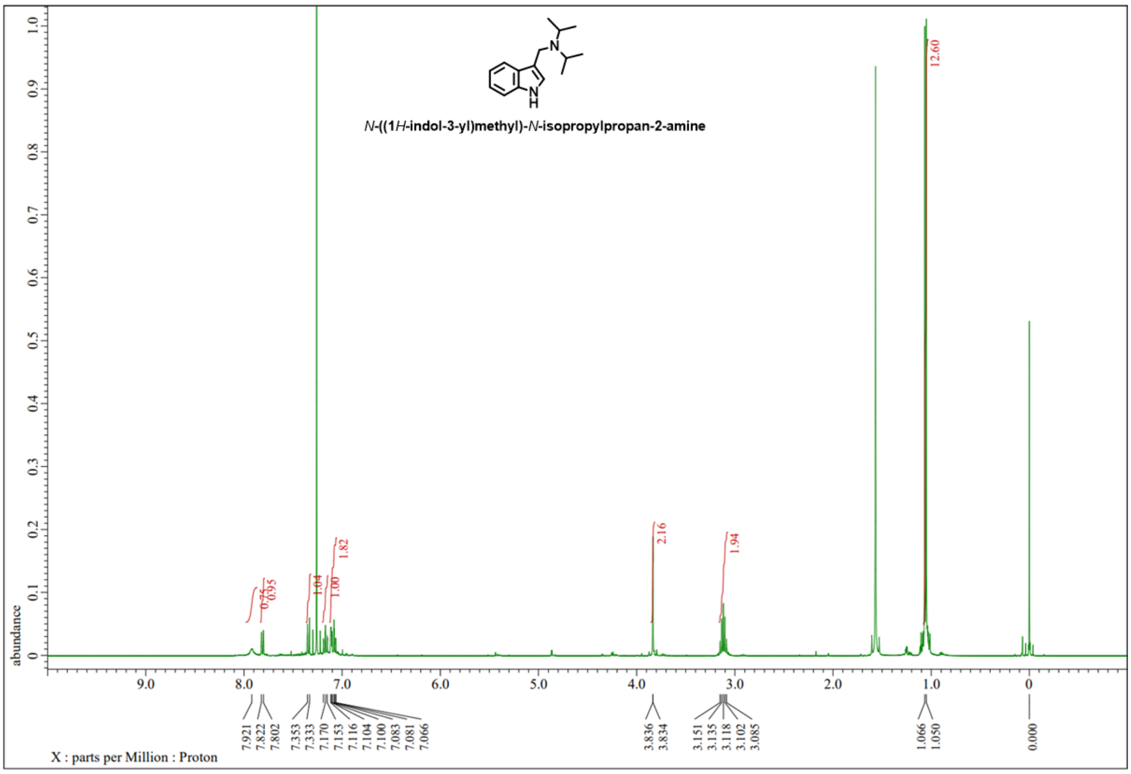

Supplementary Figure 25. Commpound **3c**, ^{1}H NMR (400 MHz, $CDCl_3$).

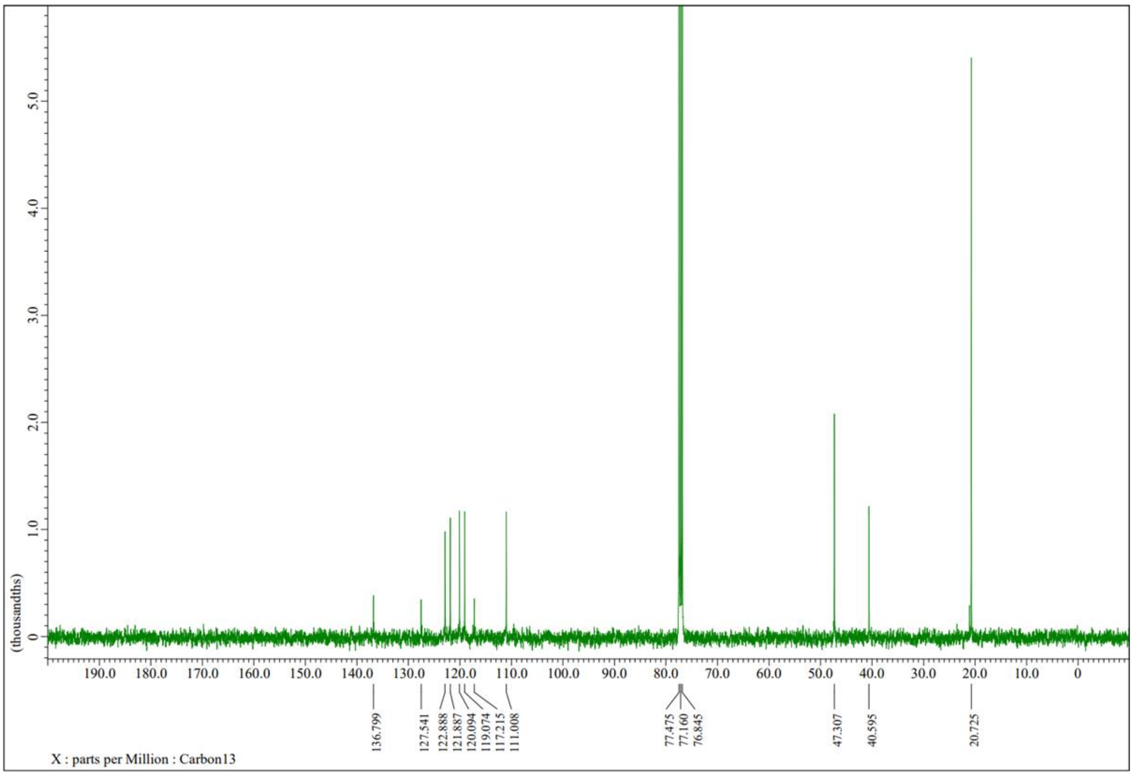

Supplementary Figure 26. Commpound **3c**, ^{13}C NMR (100 MHz, $CDCl_3$).

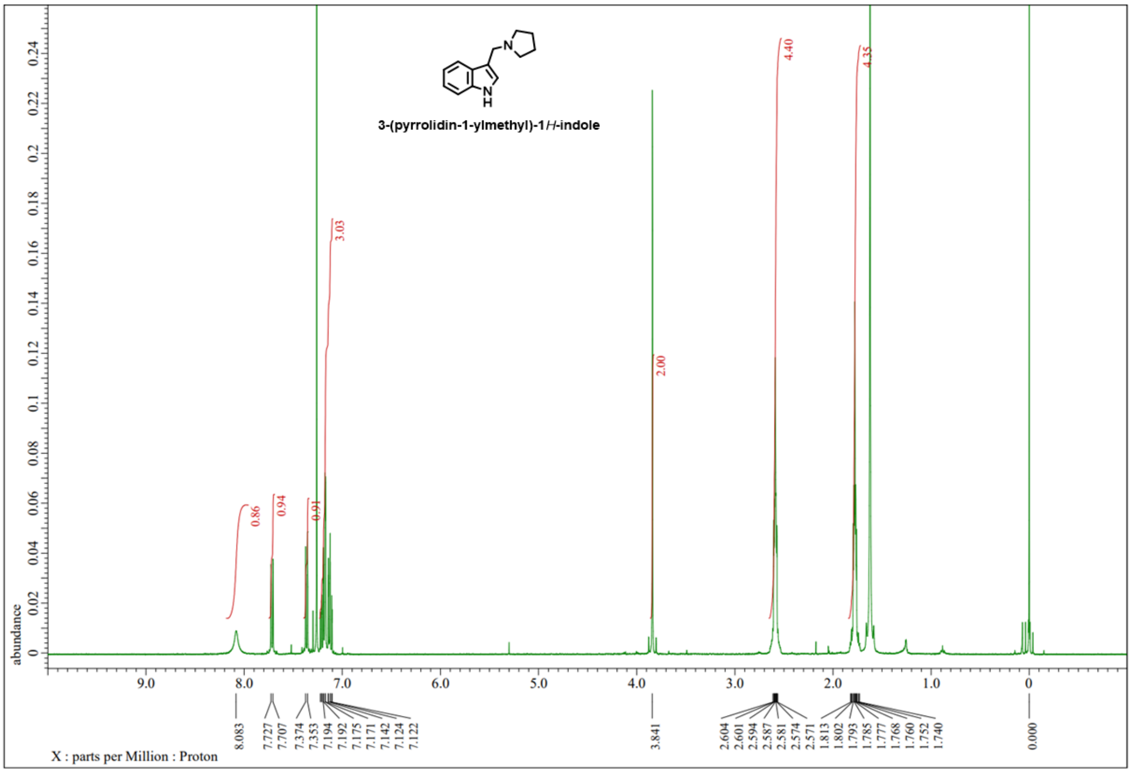

Supplementary Figure 27. Commpound **3d**, ^{1}H NMR (400 MHz, $CDCl_3$).

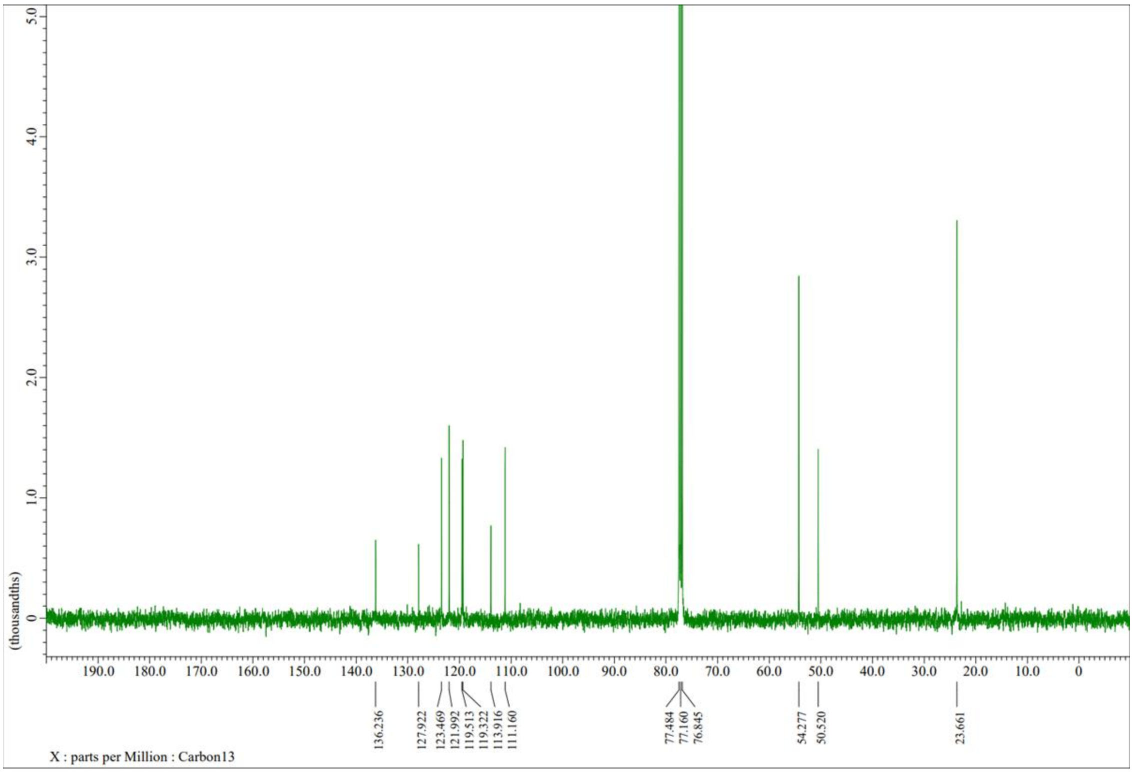

Supplementary Figure 28. Commpound **3d**, ^{13}C NMR (100 MHz, $CDCl_3$).

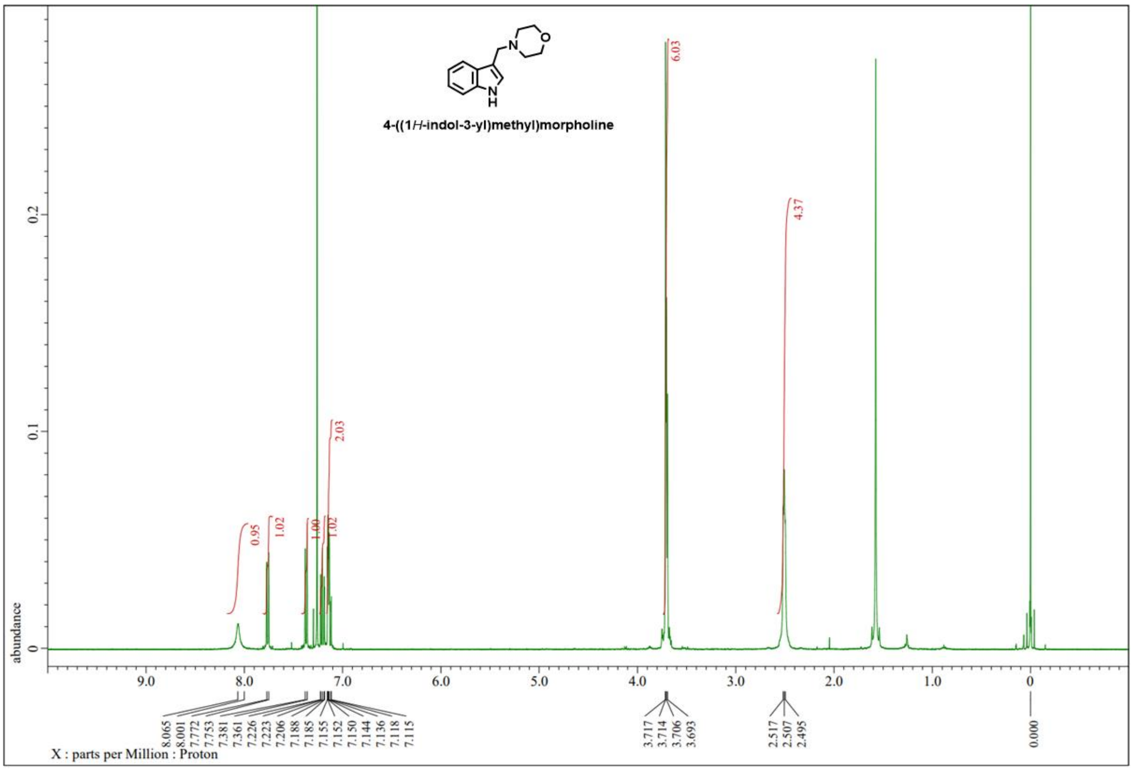

Supplementary Figure 29. Commpound **3e**, ^{1}H NMR (400 MHz, $CDCl_3$).

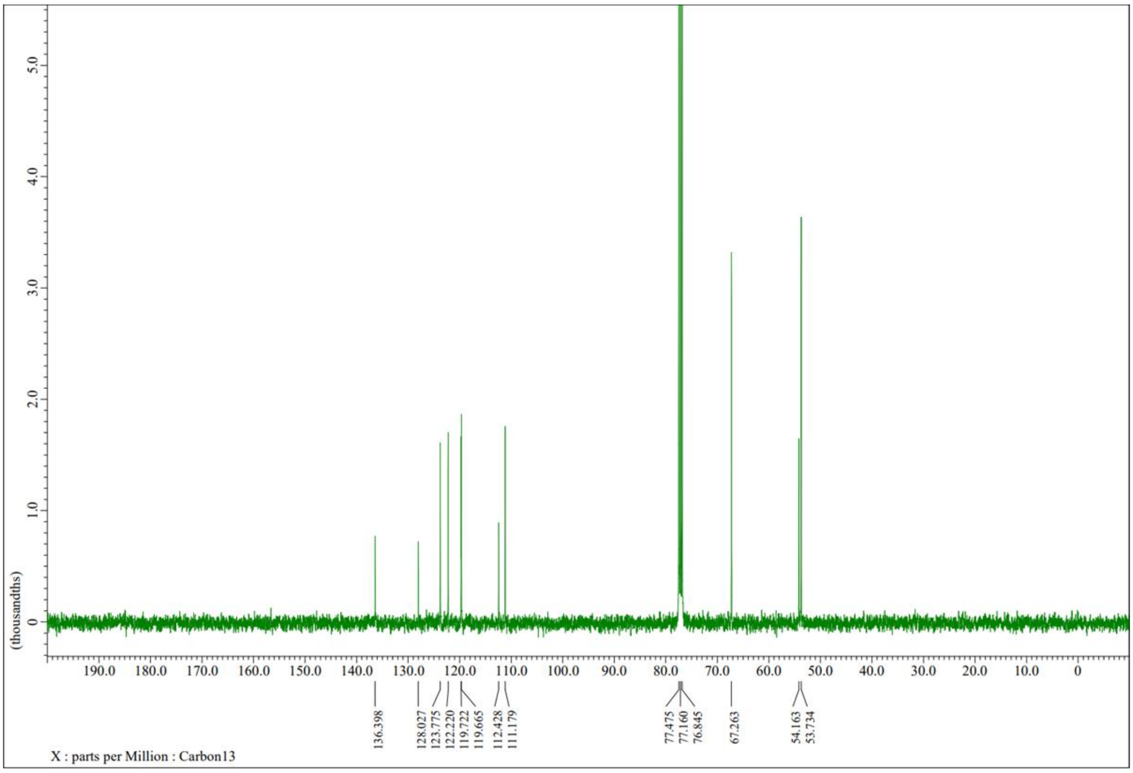

Supplementary Figure 30. Commpound **3e**, ^{13}C NMR (100 MHz, $CDCl_3$).

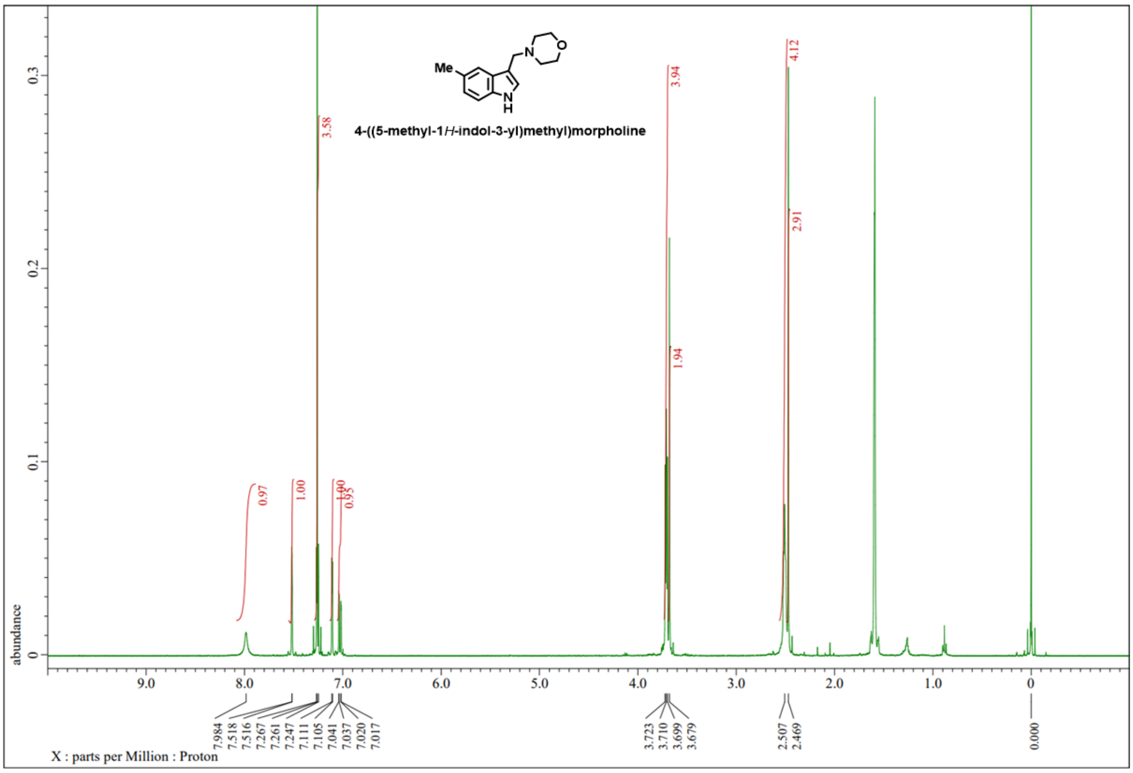

Supplementary Figure 31. Commpound **3f**, ^{1}H NMR (400 MHz, $CDCl_3$).

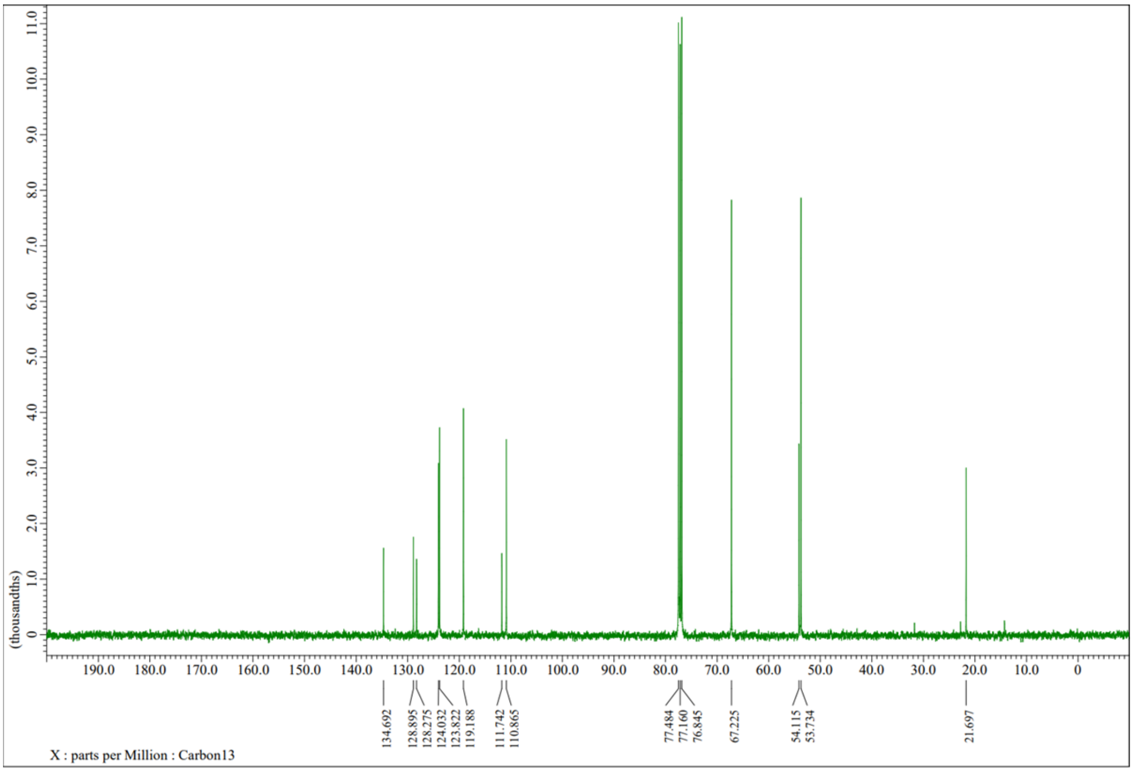

Supplementary Figure 32. Commpound **3f**, ^{13}C NMR (100 MHz, $CDCl_3$).

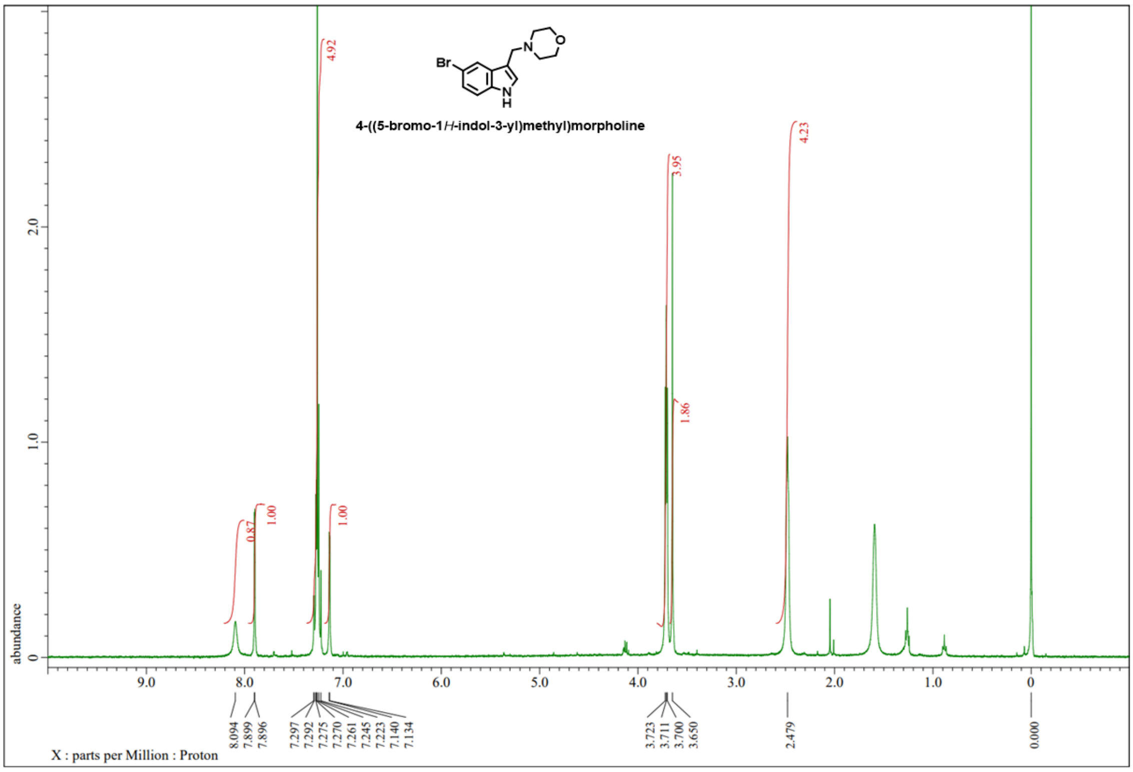

Supplementary Figure 33. Commpound **3g**, ^{1}H NMR (400 MHz, $CDCl_3$).

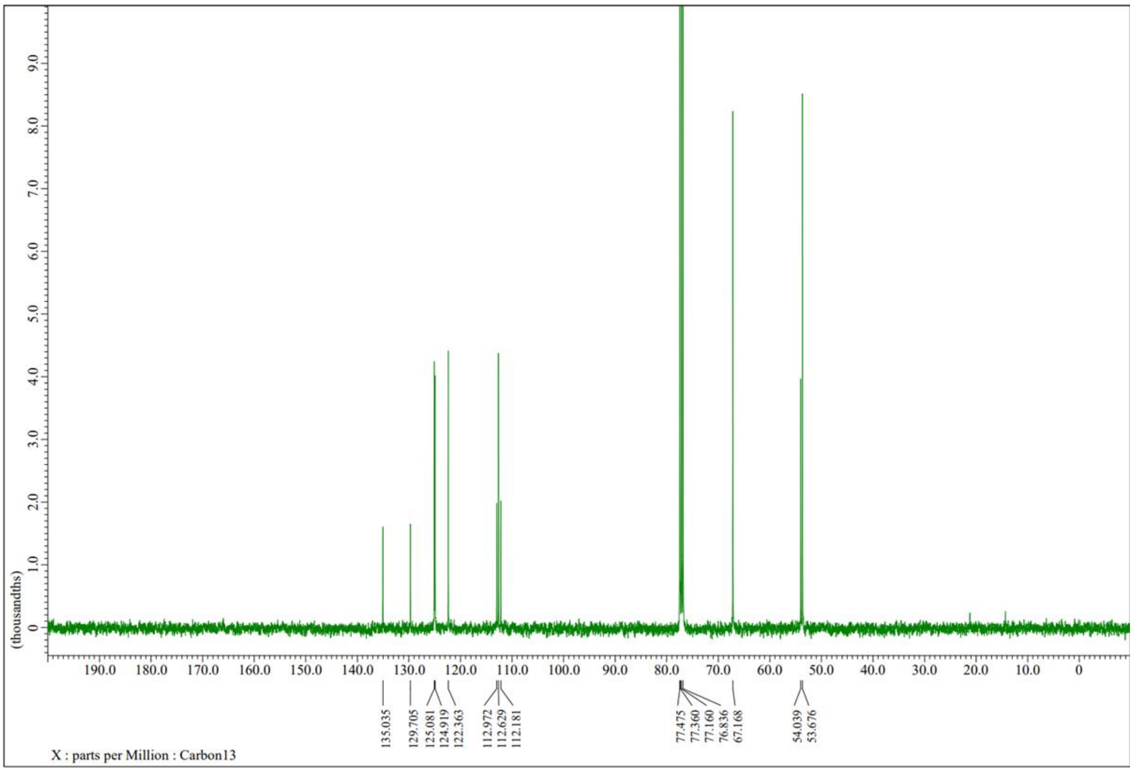

Supplementary Figure 34. Commpound **3g**, ^{13}C NMR (100 MHz, $CDCl_3$).

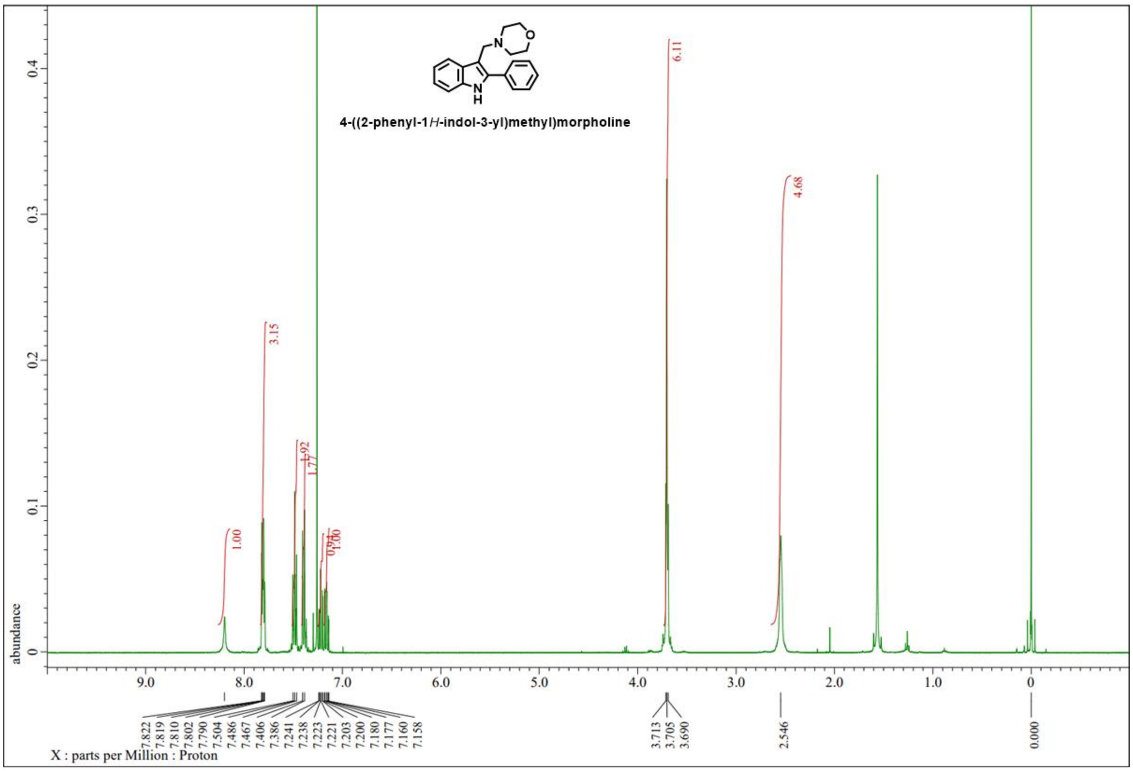

Supplementary Figure 35. Commpound **3h**, ^{1}H NMR (400 MHz, $CDCl_3$).

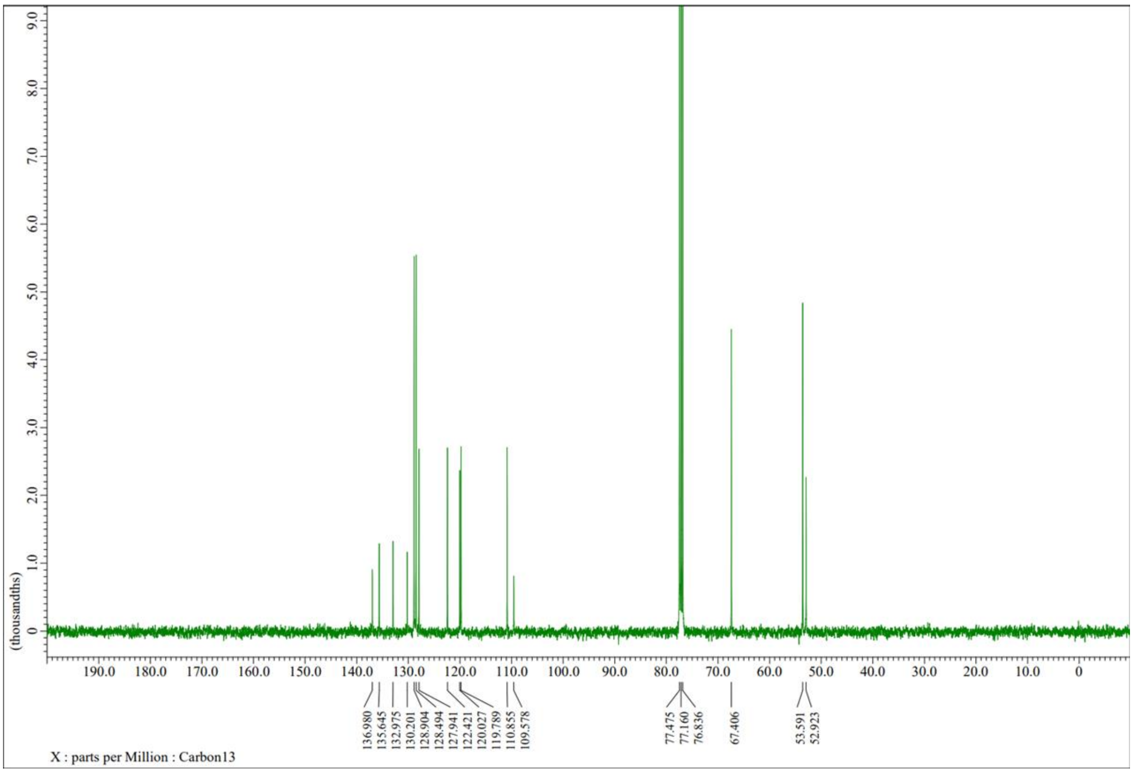

Supplementary Figure 36. Commpound **3h**, ^{13}C NMR (100 MHz, $CDCl_3$).

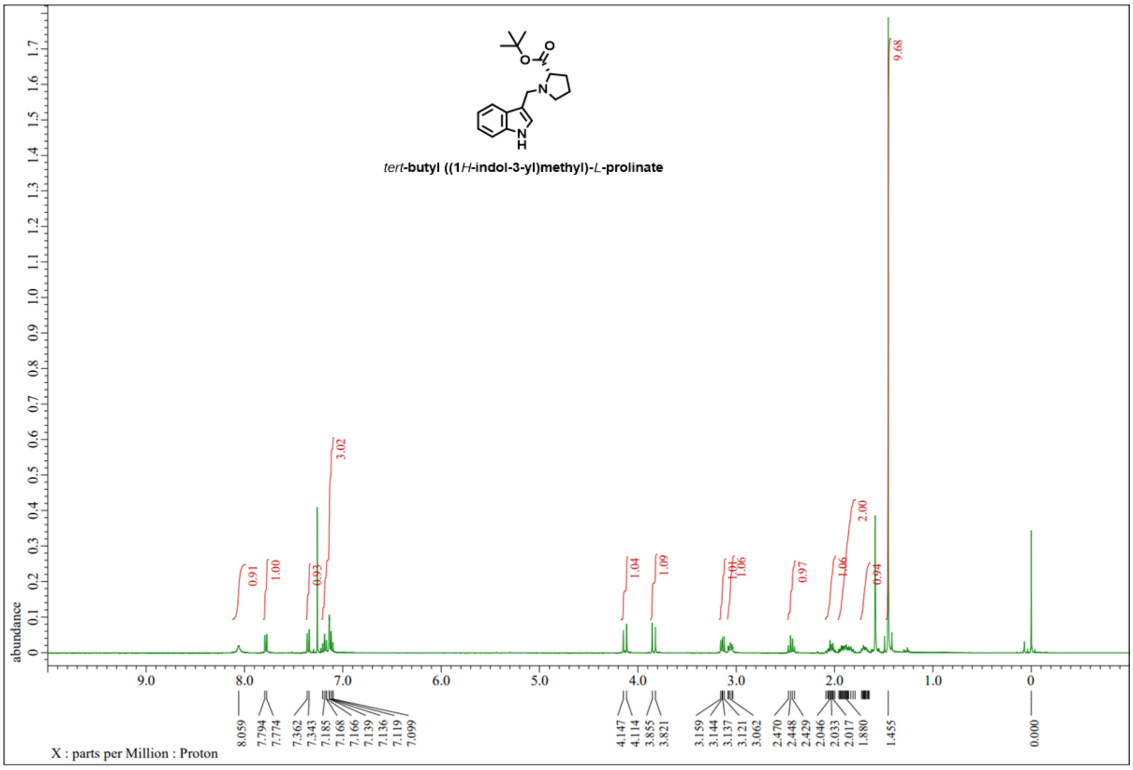

Supplementary Figure 37. Commpound **3i**, ^{1}H NMR (400 MHz, $CDCl_3$).

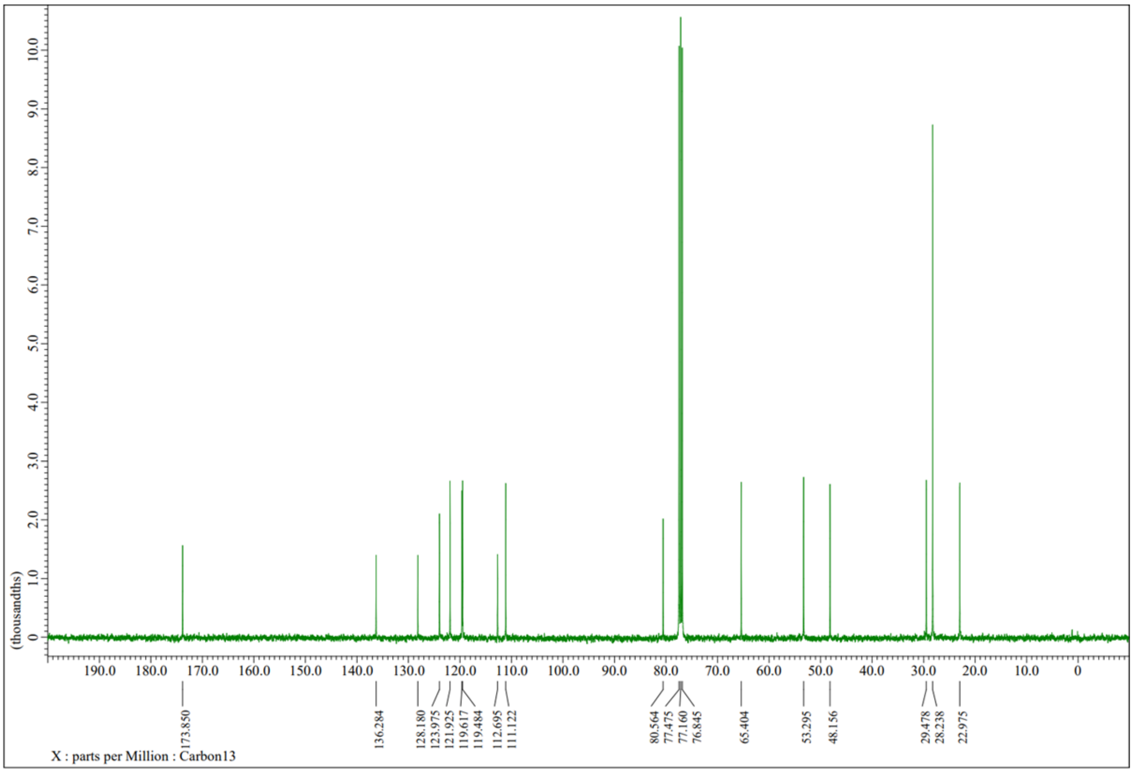

Supplementary Figure 38. Commpound **3i**, ^{13}C NMR (100 MHz, $CDCl_3$).

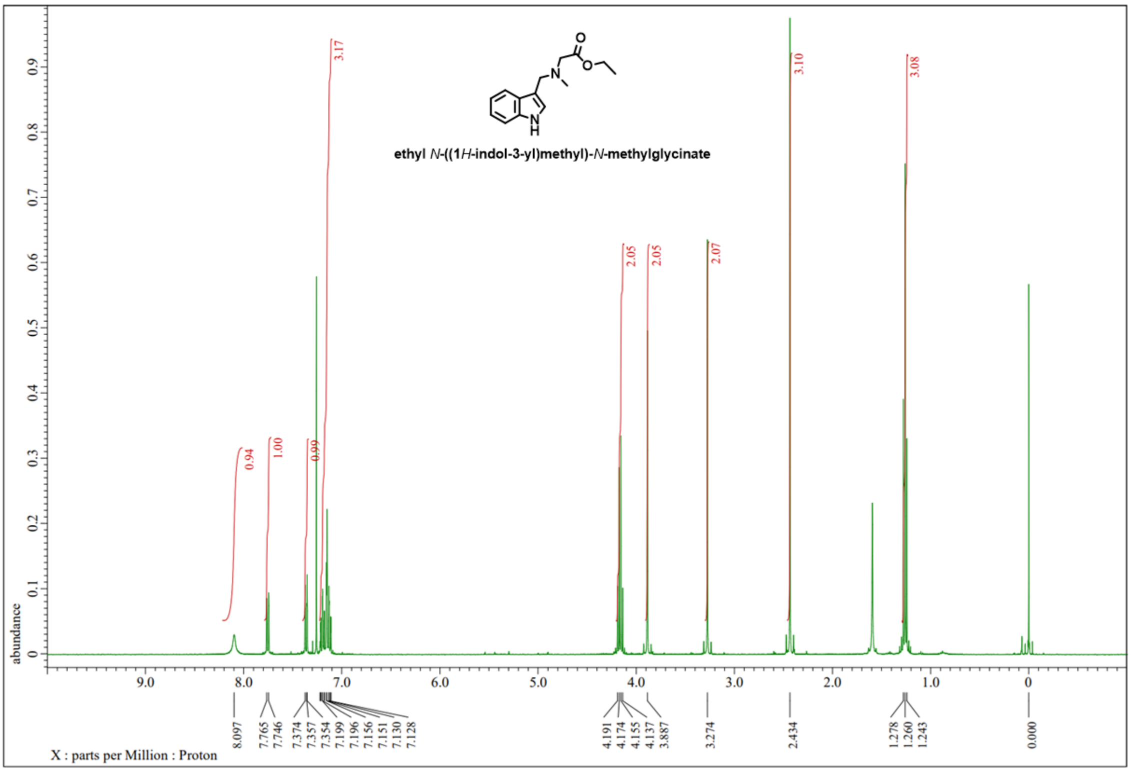

Supplementary Figure 39. Commpound **3j**, ^{1}H NMR (400 MHz, $CDCl_3$).

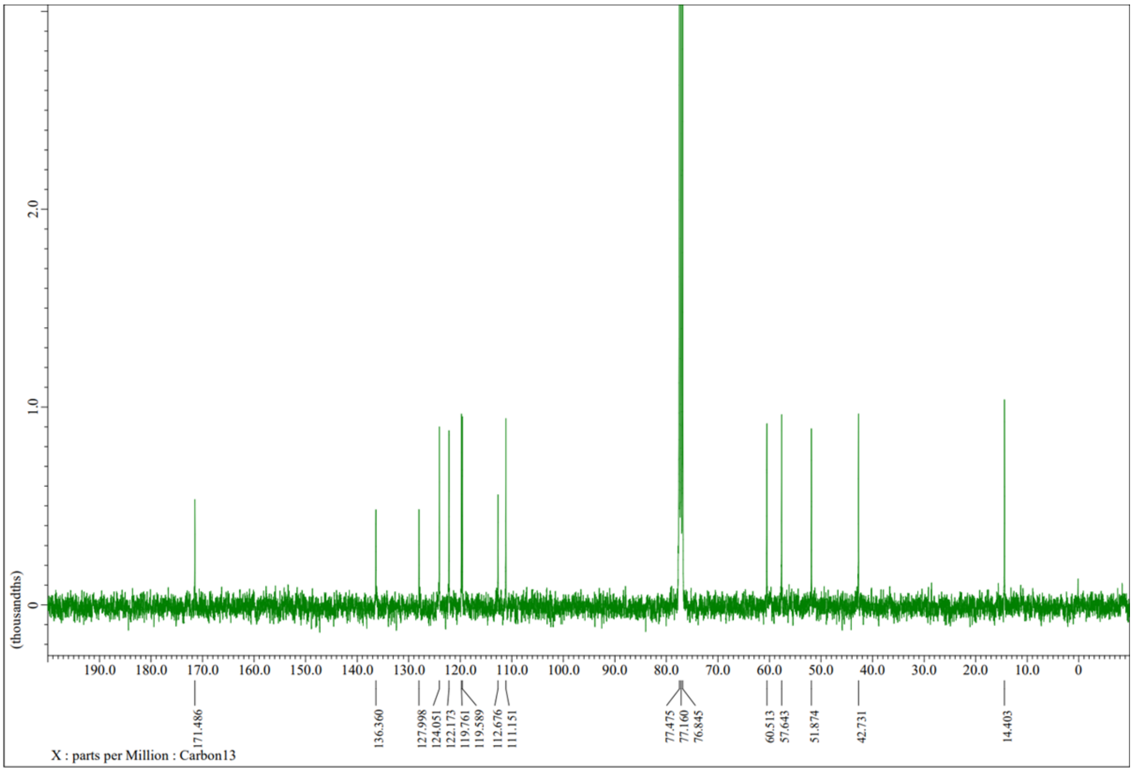

Supplementary Figure 40. Commpound **3j**, ^{13}C NMR (100 MHz, $CDCl_3$).

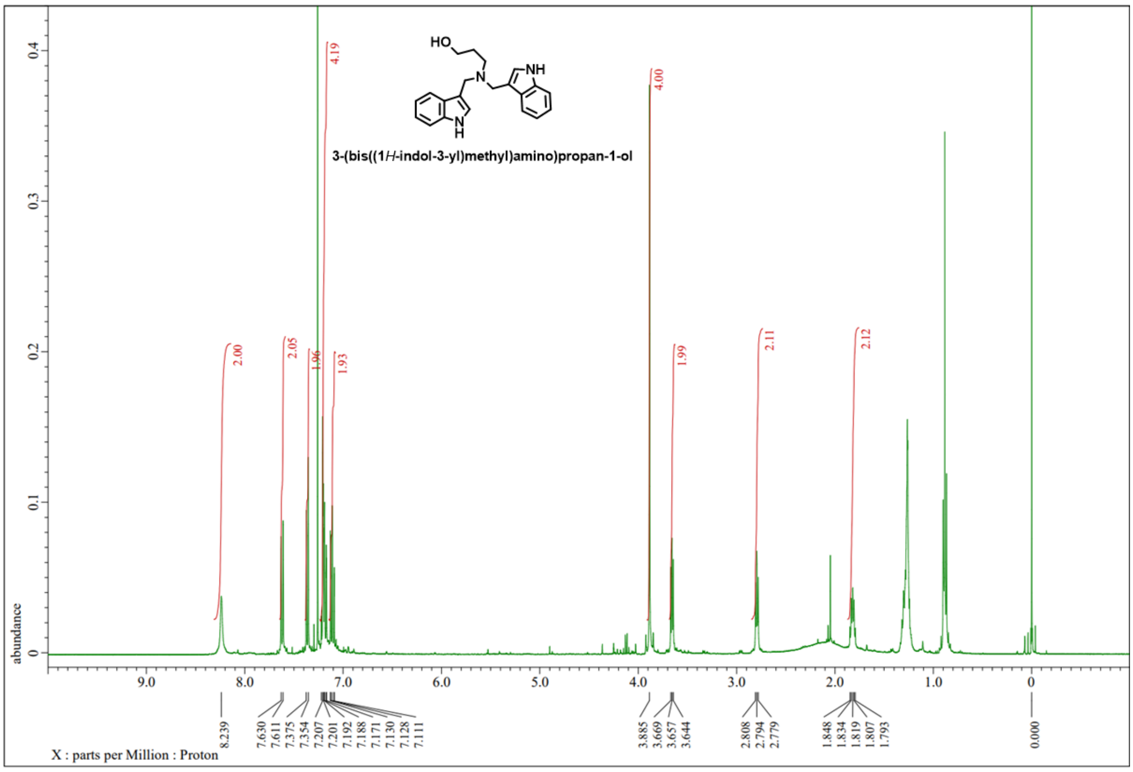

Supplementary Figure 41. Commpound **3k**, ^{1}H NMR (400 MHz, $CDCl_3$).

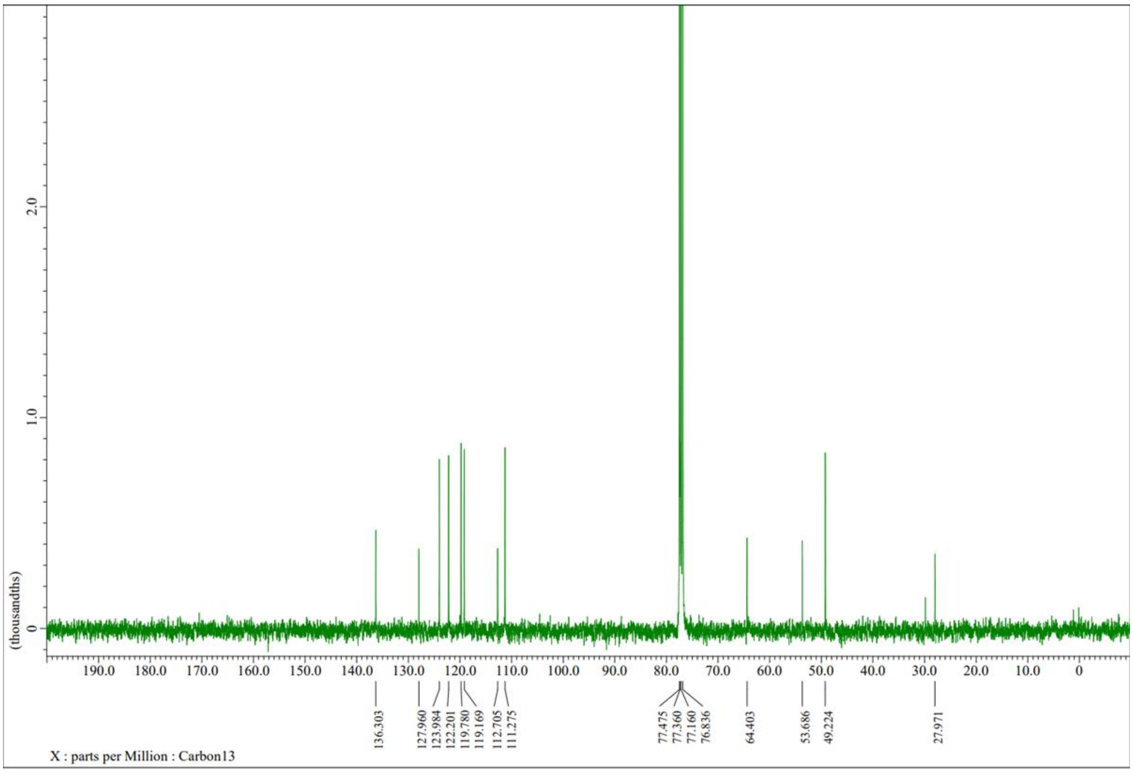

Supplementary Figure 42. Commpound **3k**, ^{13}C NMR (100 MHz, $CDCl_3$).

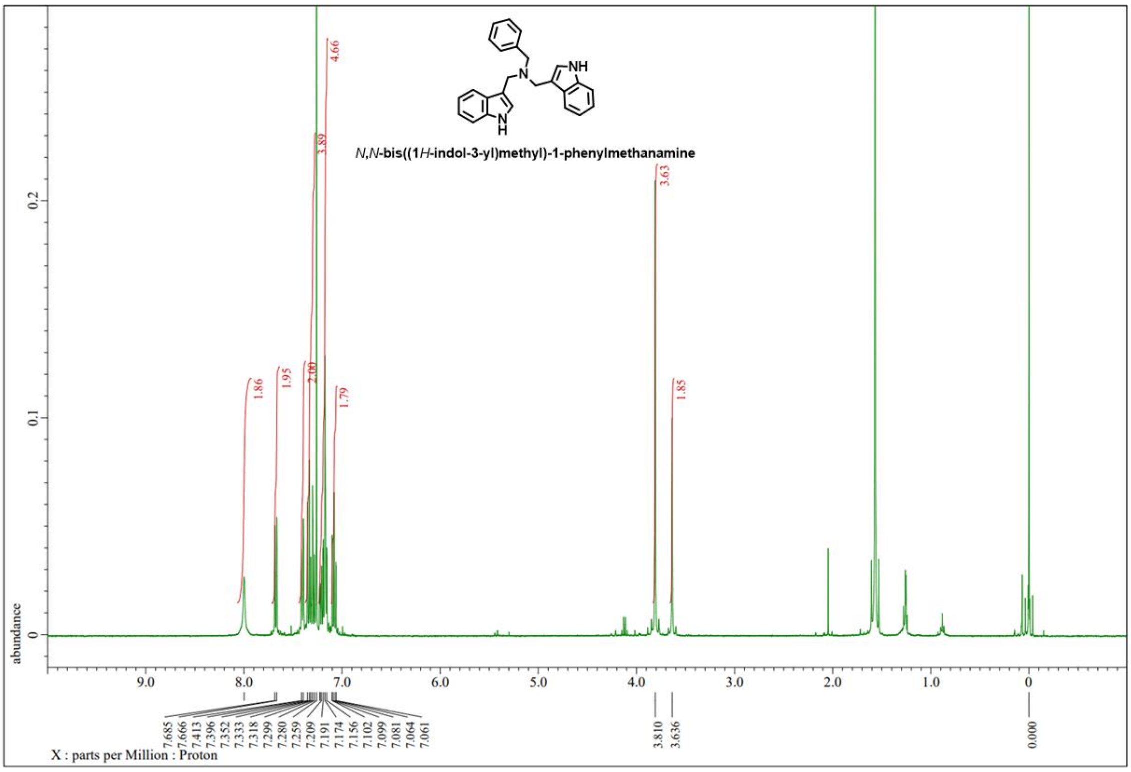

Supplementary Figure 43. Commpound **3l**, ^{1}H NMR (400 MHz, $CDCl_3$).

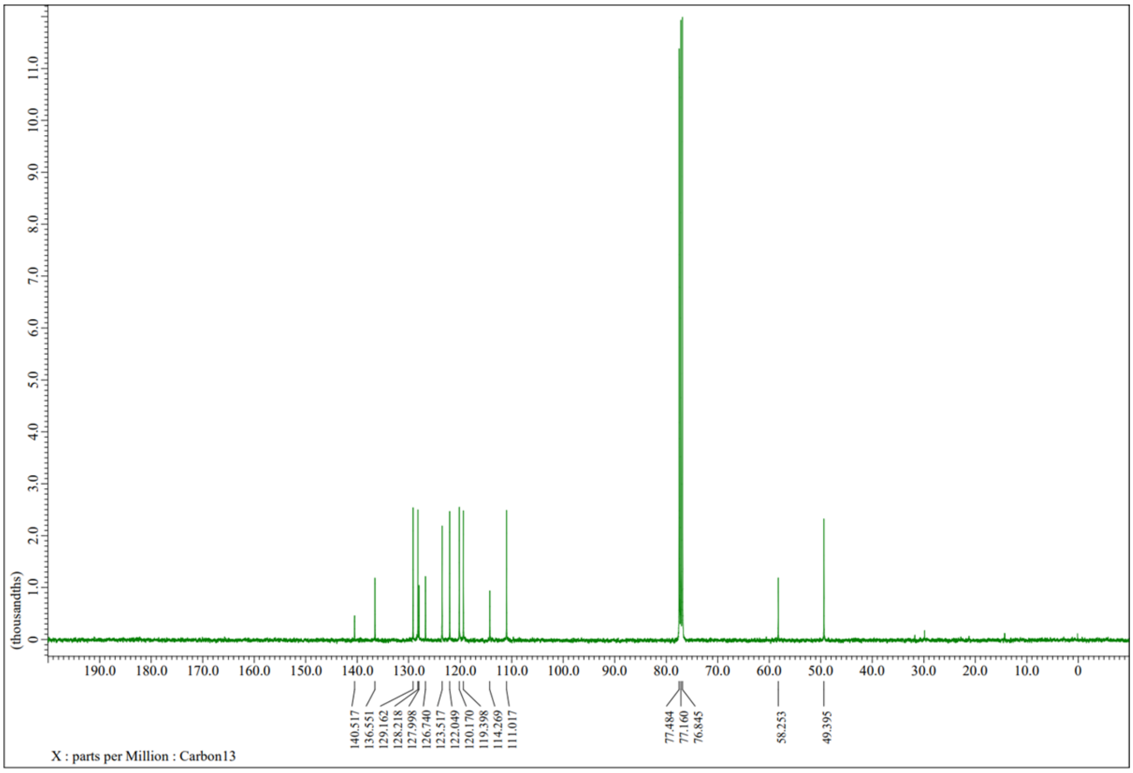

Supplementary Figure 44. Commpound **3l**, ^{13}C NMR (100 MHz, $CDCl_3$).

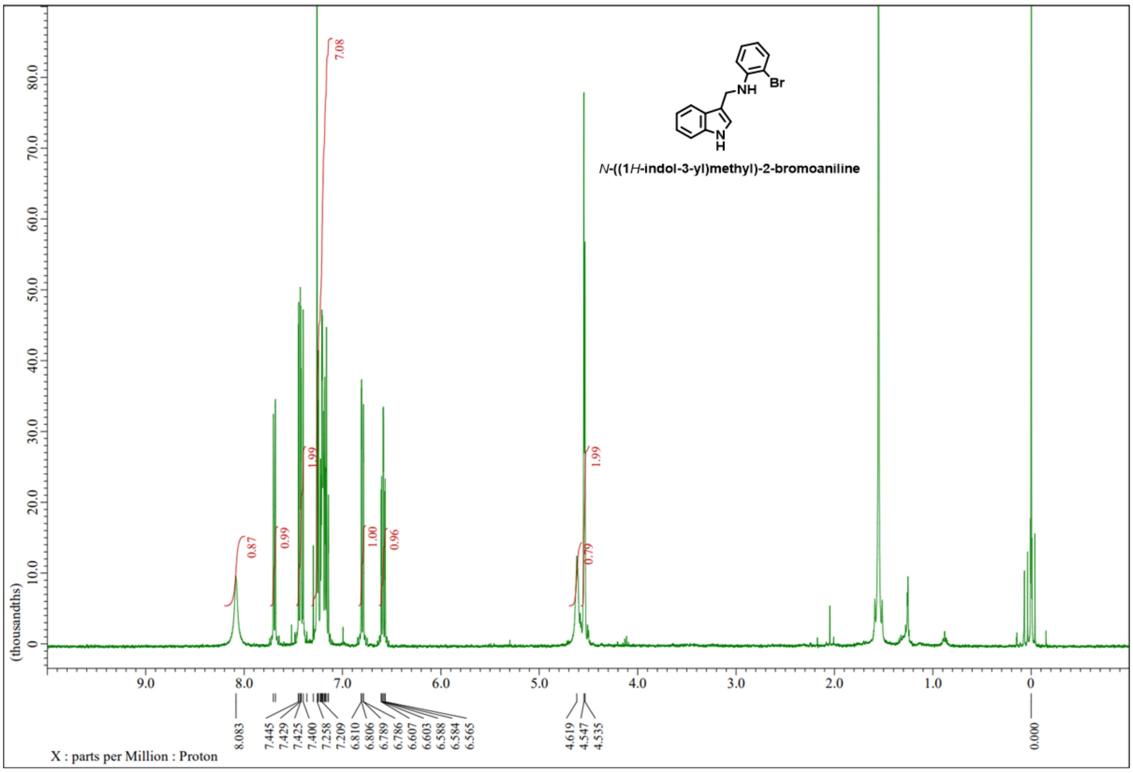

Supplementary Figure 45. Commpound **3m**, ^{1}H NMR (400 MHz, $CDCl_3$).

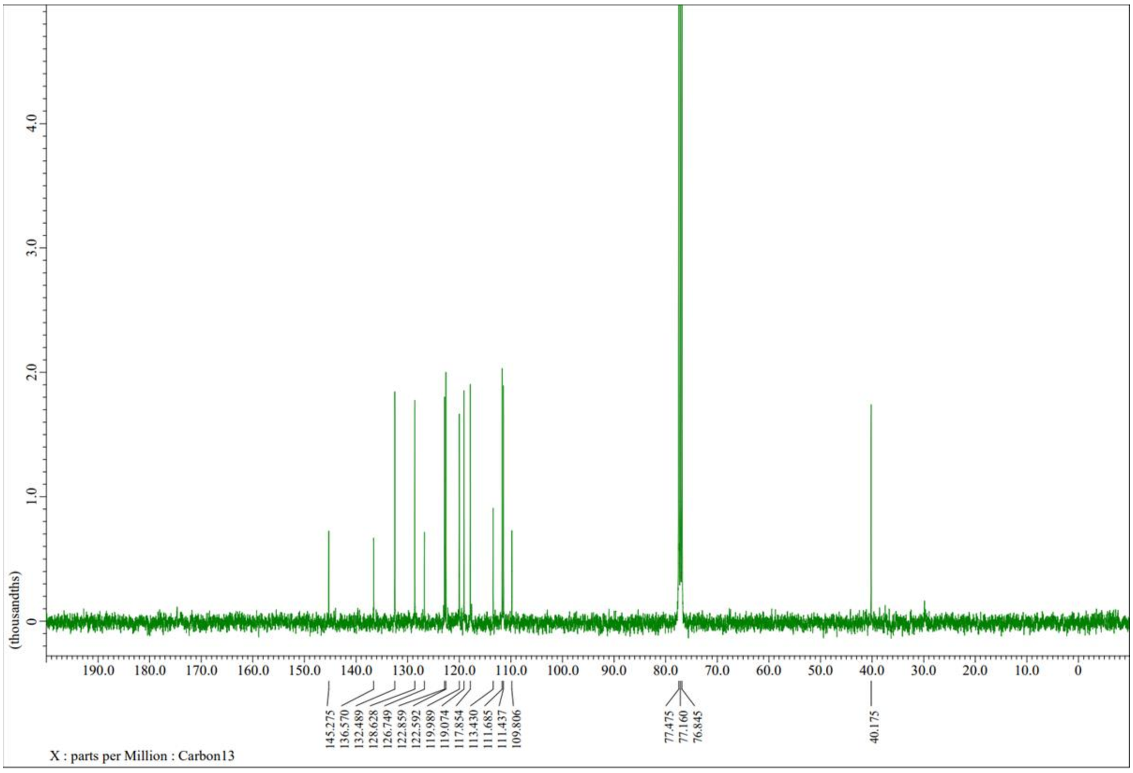

Supplementary Figure 46. Commpound **3m**, ^{13}C NMR (100 MHz, $CDCl_3$).

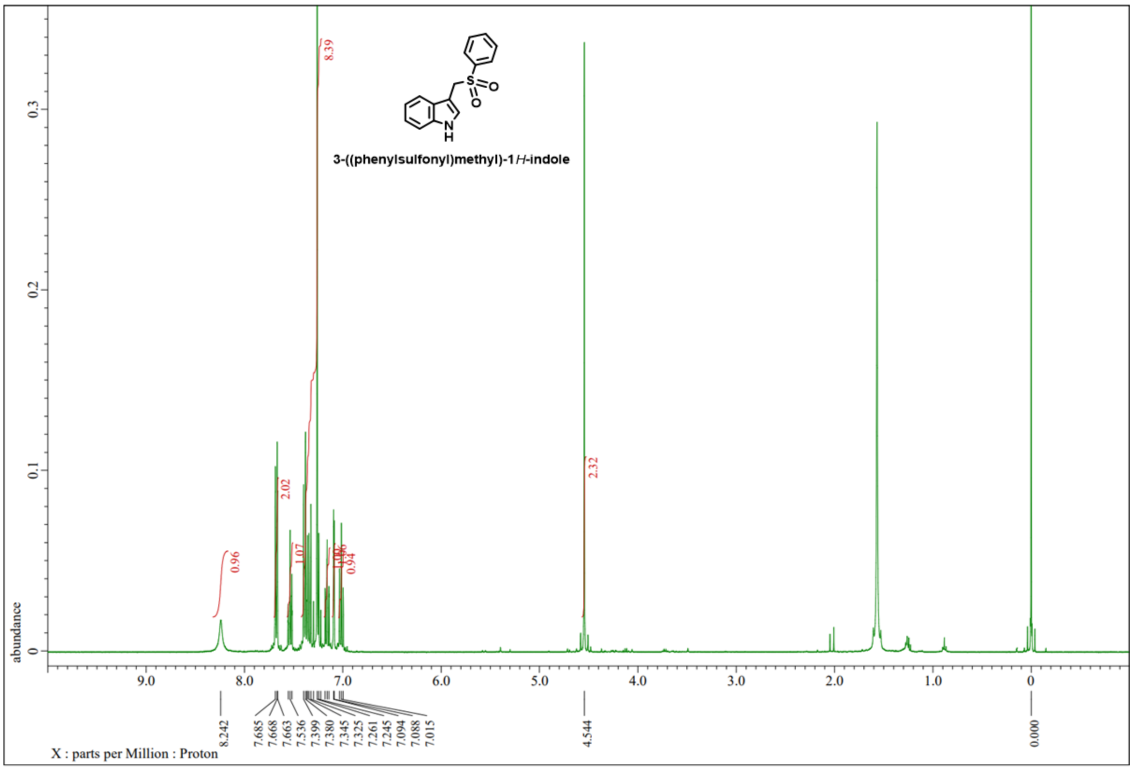

Supplementary Figure 47. Commpound **3n**, ^{1}H NMR (400 MHz, $CDCl_3$).

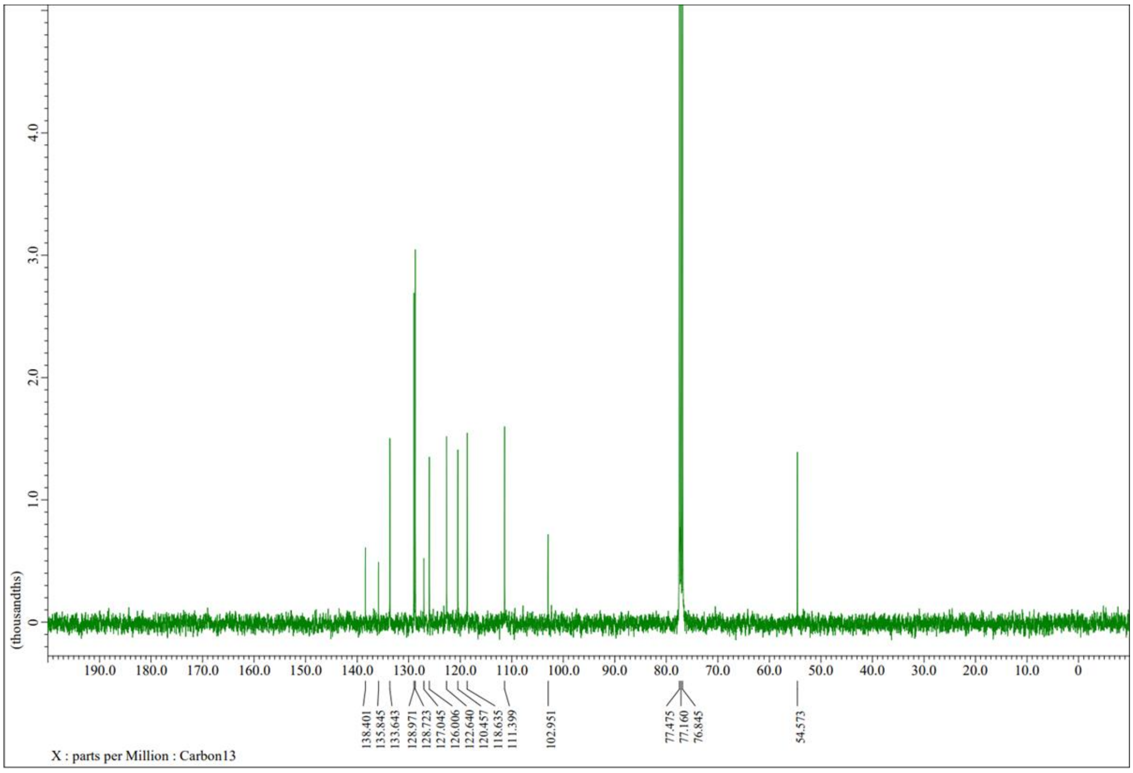

Supplementary Figure 48. Commpound **3n**, ^{13}C NMR (100 MHz, $CDCl_3$).

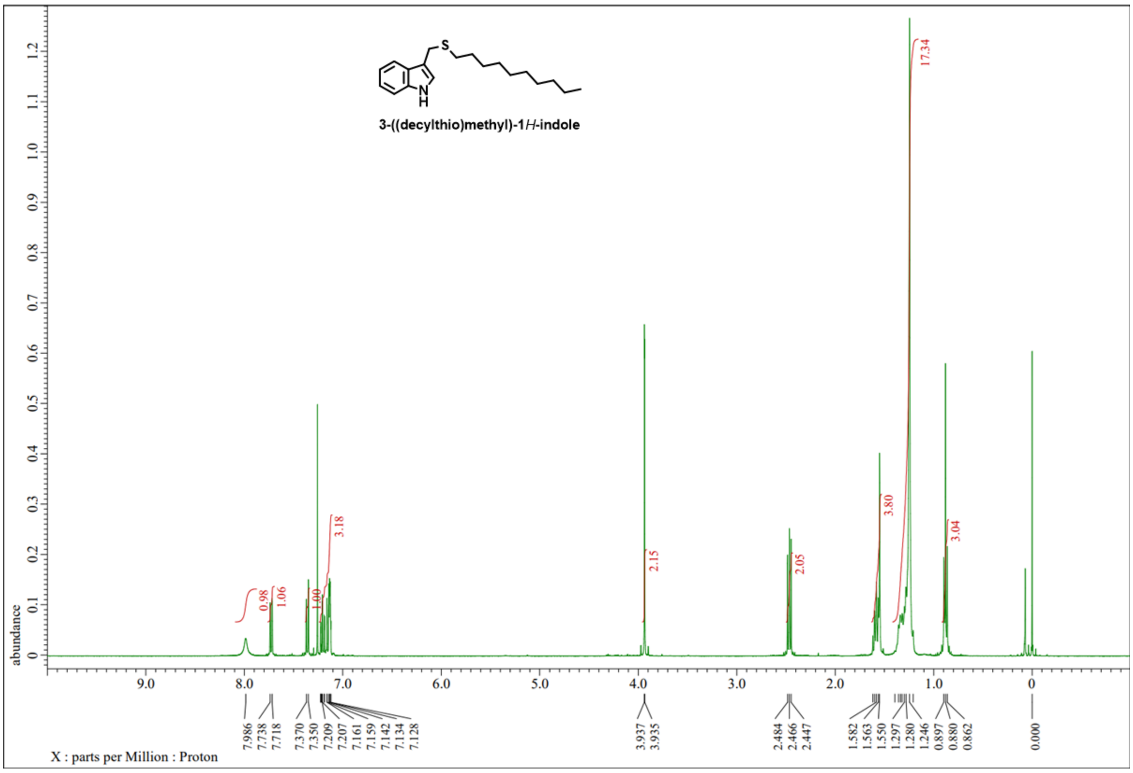

Supplementary Figure 49. Commpound **3o**, ^{1}H NMR (400 MHz, $CDCl_3$).

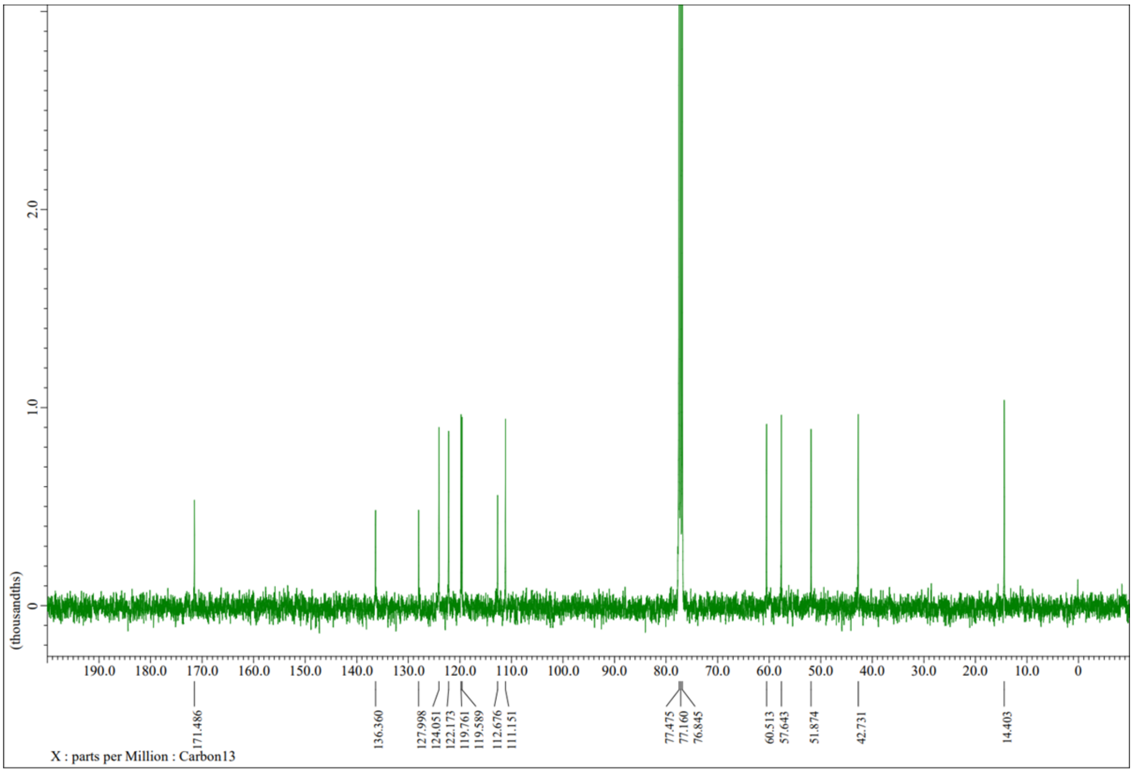

Supplementary Figure 50. Commpound **3o**, ^{13}C NMR (100 MHz, $CDCl_3$).

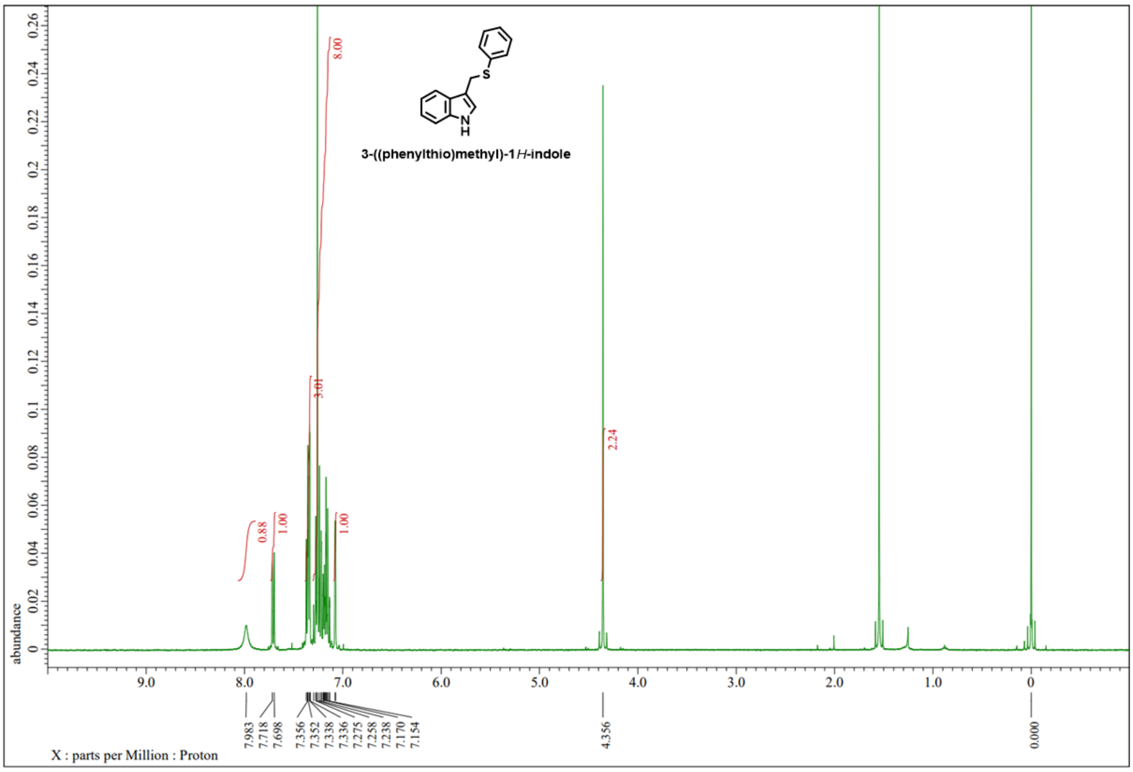

Supplementary Figure 51. Commpound **3p**, ^{1}H NMR (400 MHz, $CDCl_3$).

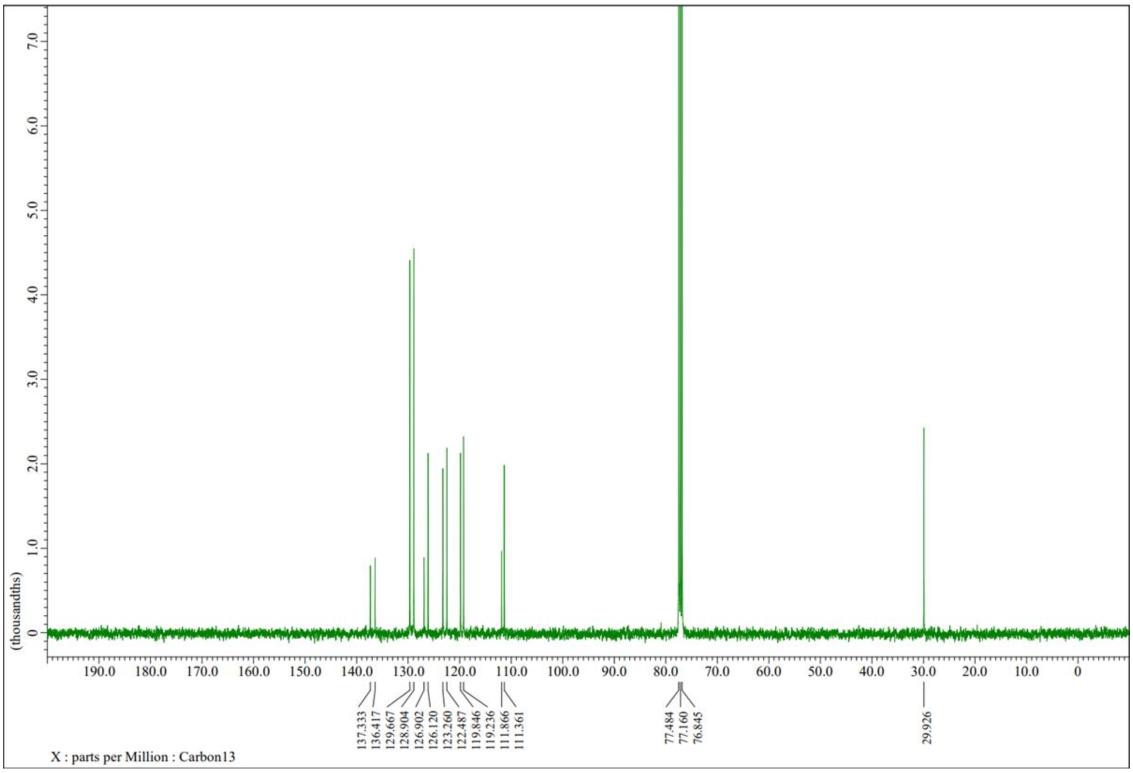

Supplementary Figure 52. Commpound **3p**, ^{13}C NMR (100 MHz, $CDCl_3$).

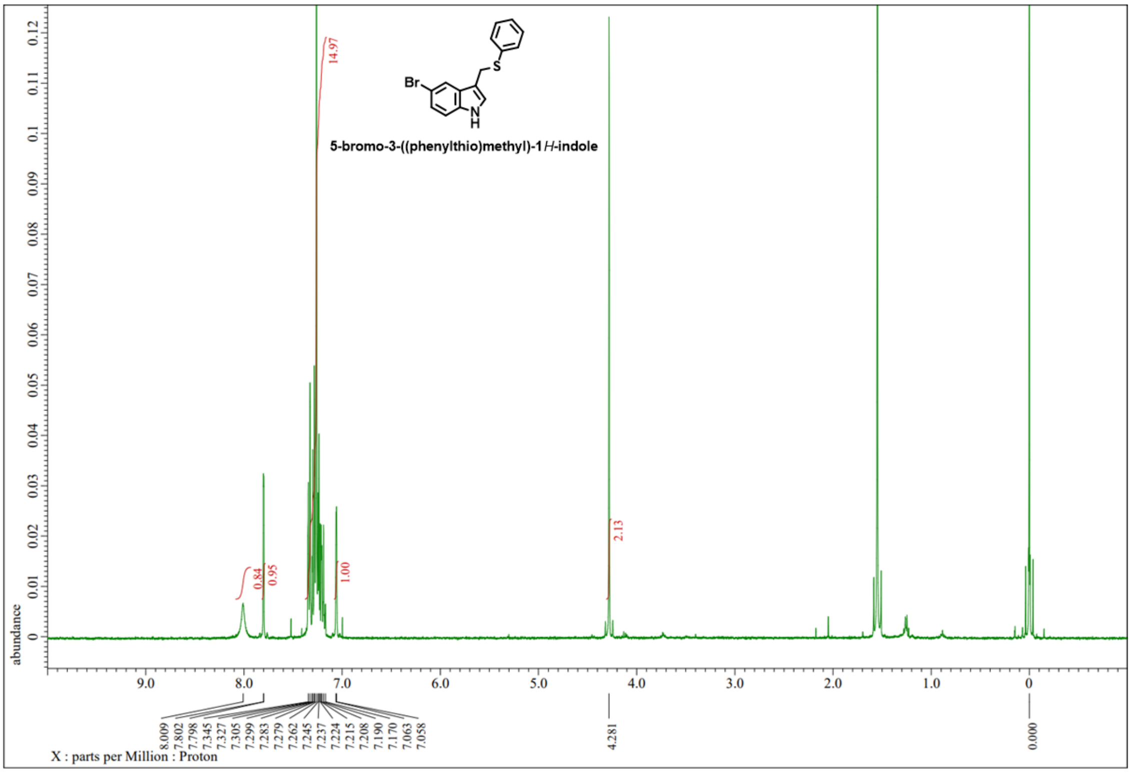

Supplementary Figure 53. Commpound **3q**, ^{1}H NMR (400 MHz, $CDCl_3$).

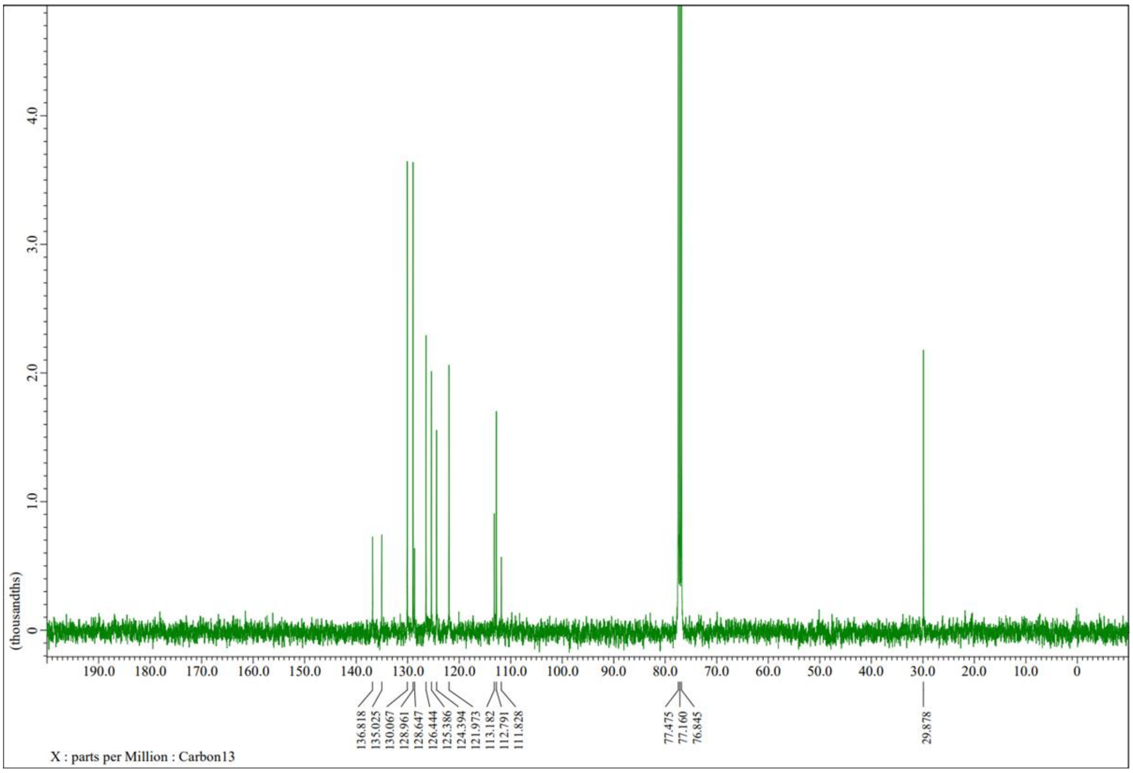

Supplementary Figure 54. Commpound **3q**, ^{13}C NMR (100 MHz, $CDCl_3$).

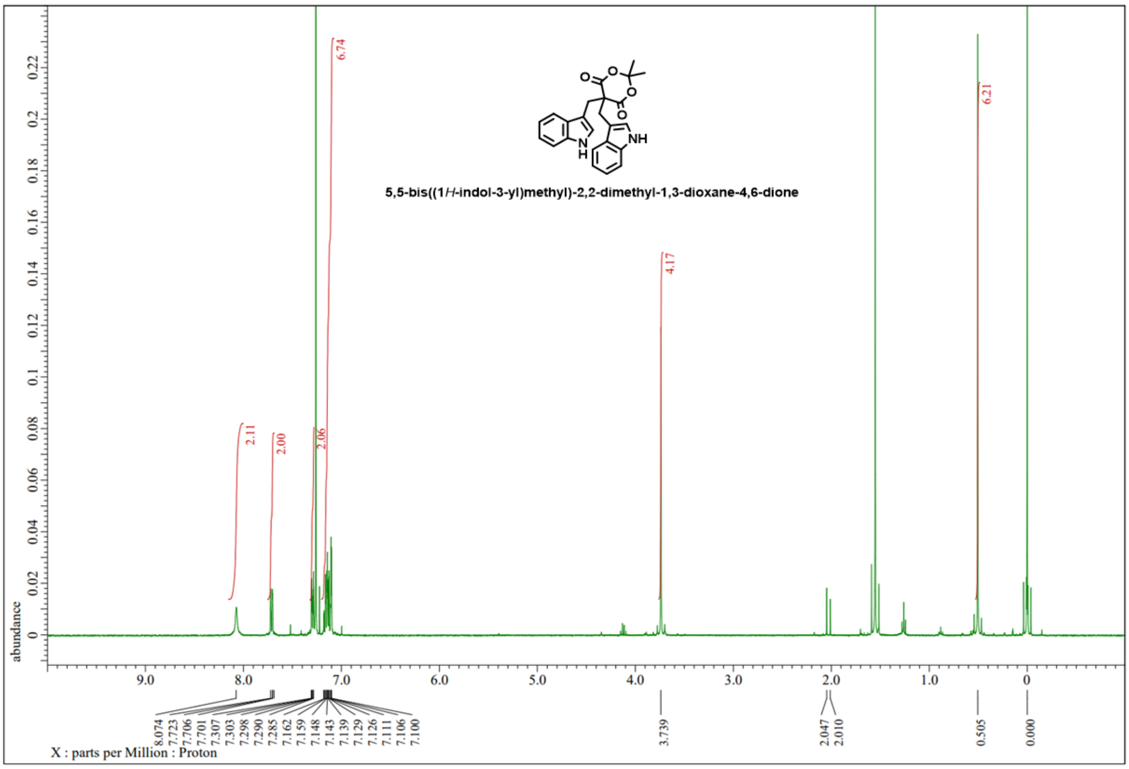

Supplementary Figure 55. Commpound **3r**, ^{1}H NMR (400 MHz, $CDCl_3$).

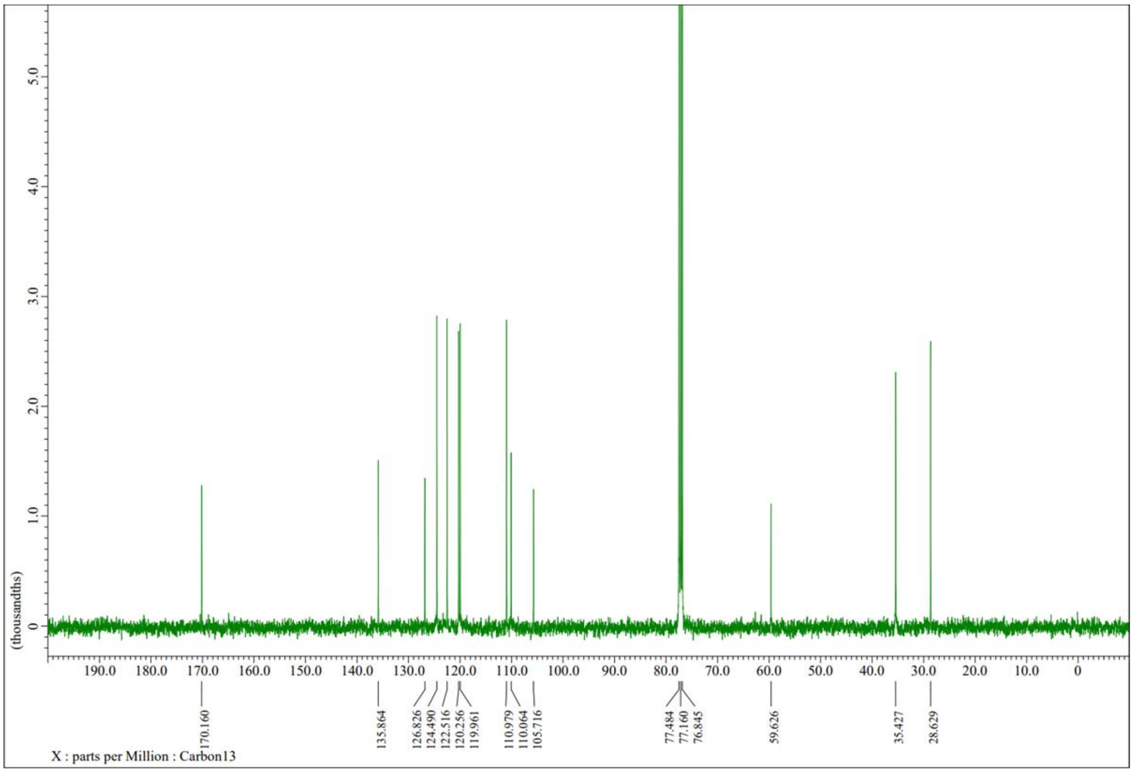

Supplementary Figure 56. Commpound **3r**, ^{13}C NMR (100 MHz, $CDCl_3$).

Supplementary References

[1] B. Y. Eryshev, A. G. Dubinin, V. N. Buyanov, N. N. Suvorov, *Chem. Heterocycl. Compd.* **1974**, *10*, 1313–1315.

[2] M. U. Ahmad, L. M. Libbey, R. A. Scanlan, *Food Addit. Contam.* **1987**, *4*, 45–48.

[3] B. Mekonnen Sanka, D. Mamo Tadesse, E. Teju Bedada, E. T. Mengesha, N. Babu G, *Bioorg. Chem.* **2022**, *119*, 105568.

[4] X. Chen, H. Fan, S. Zhang, C. Yu, W. Wang, *Chem. Eur. J.* **2016**, *22*, 716–723.

[5] M. M. Faul, L. L. Winneroski, C. A. Krumrich, *J. Org. Chem.* **1999**, *64*, 2465–2470.

[6] Frisch, M. J.; Trucks, G. W.; Schlegel, H. B.; Scuseria, G. E.; Robb, M. A.; Cheeseman, J. R.; Scalmani, G.; Barone, V.; Petersson, G. A.; Nakatsuji, H.; Li, X.; Caricato, M.; Marenich, A. V.; Bloino, J.; Janesko, B. G.; Gomperts, R.; Mennucci, B.; Hratchian, H. P.; Ortiz, J. V.; Izmaylov, A. F.; Sonnenberg, J. L.; WilliamsYoung, D.; Ding, F.; Lipparini, F.; Egidi, F.; Goings, J.; Peng, B.; Petrone, A.; Henderson, T.; Ranasinghe, D.; Zakrzewski, V. G.; Gao, J.; Rega, N.; Zheng, G.; Liang, W.; Hada, M.; Ehara, M.; Toyota, K.; Fukuda, R.; Hasegawa,J.; Ishida, M.; Nakajima, T.; Honda, Y.; Kitao, O.; Nakai, H.; Vreven, T.; Throssell, K.; Montgomery, J. A., Jr.; Peralta, J. E.; Ogliaro, F.; Bearpark, M. J.; Heyd, J. J.; Brothers, E. N.; Kudin, K. N.; Staroverov, V. N.; Keith, T. A.; Kobayashi, R.; Normand, J.; Raghavachari, K.; Rendell, A. P.; Burant, J. C.; Iyengar, S. S.; Tomasi, J.; Cossi, M.; Millam, J. M.; Klene, M.; Adamo, C.; Cammi, R.; Ochterski, J. W.; Martin, R. L.; Morokuma, K.; Farkas, O.; Foresman,J. B.; Fox, D. J. *Gaussian 16*, Revision B.01, Gaussian, Inc., Wallingford CT, 2016.

[7] M. K. Akkoc, M. Y. Yuksel, I. Durmaz, R. Ç. Atalay, *Turk. J. Chem.* **2012**, *36*, 515–525.

[8] Y. G. İşgör, Z. Kılıç, S. Ölgen, *Chem. Biol. Drug Des.* **2008**, *72*, 599–604.

[9] M. H. S. A. Hamid, C. L. Allen, G. W. Lamb, A. C. Maxwell, H. C. Maytum, A. J. A. Watson, J. M. J. Williams, *J. Am. Chem. Soc.* **2009**, *131*, 1766–1774.

[10] S. Prabhu, Z. Akbar, F. Harris, K. Karakoula, R. Lea, F. Rowther, T. Warr, T. Snape, *Bioorg. Med. Chem.* **2013**, *21*, 1918–1924.

[11] S. Lu, Y.-S. Zhu, K.-X. Yan, T.-W. Cui, X. Zhu, X.-Q. Hao, M.-P. Song, *Synlett* **2019**, *30*, 1924–1928.

[12] V. F. Patel, G. Pattenden, D. M. Thompson, *J. Chem. Soc., Perkin Trans. 1* **1990**, 2729–2734.
