# Supplementary material for: Verification of preparations of (1H-indol-3-yl)methyl electrophiles and development of their microflow rapid generation and substitution
Source: Commun Chem. 2023 Mar 4;6:47. doi: 10.1038/s42004-023-00837-1 (PMC9985609; doi:10.1038/s42004-023-00837-1)
Supplement: Supplementary file 2 — Description of Additional Supplementary Files [file 42004_2023_837_MOESM2_ESM.pdf]

# Description of Additional Supplementary File

**File name:** Supplementary Data

**Description:** CIF file of X-ray crystallographic data of compound <b>19</b>
